# Aptamer-functionalized triptolide with release controllability as a promising targeted therapy against triple-negative breast cancer

**DOI:** 10.1186/s13046-024-03133-5

**Published:** 2024-07-25

**Authors:** Yao Chen, Jirui Yang, Chuanqi Wang, Tianbao Wang, Yingjie Zeng, Xiao Li, Yi Zuo, Hongyu Chen, Chaozheng Zhang, Yuening Cao, Chen Sun, Maolin Wang, Xiujun Cao, Xian Ge, Yilan Liu, Ge Zhang, Yun Deng, Cheng Peng, Aiping Lu, Jun Lu

**Affiliations:** 1 State Key Laboratory of Southwestern Chinese Medicine Resources, School of Pharmacy, Chengdu University of Traditional Chinese Medicine, Chengdu, 611137 China; 2 Sichuan Clinical Research Center for Cancer, Sichuan Cancer Hospital & Institute, Sichuan Cancer Center, Affiliated Cancer Hospital of University of Electronic Science and Technology of China, Chengdu, 610041 China; 3 Clinical Research Center, The First Affiliated Hospital of Shantou University Medical College, Shantou, 515000 Guangdong Province China; 4 Hematology Department, The General Hospital of the Western Theater Command PLA, Chengdu, 611137 China; 5 Institute for Advancing Translational Medicine in Bone & Joint Diseases, School of Chinese Medicine, Hong Kong Baptist University, Hong Kong SAR, 999077 China

**Keywords:** Triple-negative breast cancer, Aptamer, Triptolide, pH-hypersensitive, Targeted therapeutic agent

## Abstract

Targeted delivery and precise release of toxins is a prospective strategy for the treatment of triple-negative breast cancer (TNBC), yet the flexibility to incorporate both properties simultaneously remains tremendously challenging in the X-drug conjugate fields. As critical components in conjugates, linkers could flourish in achieving optimal functionalities. Here, we pioneered a pH-hypersensitive tumor-targeting aptamer AS1411-triptolide conjugate (AS-TP) to achieve smart release of the toxin and targeted therapy against TNBC. The multifunctional acetal ester linker in the AS-TP site-specifically blocked triptolide toxicity, quantitatively sustained aptamer targeting, and ensured the circulating stability. Furthermore, the aptamer modification endowed triptolide with favorable water solubility and bioavailability and facilitated endocytosis of conjugated triptolide by TNBC cells in a nucleolin-dependent manner. The integrated superiorities of AS-TP promoted the preferential intra-tumor triptolide accumulation in xenografted TNBC mice and triggered the in-situ triptolide release in the weakly acidic tumor microenvironment, manifesting striking anti-TNBC efficacy and virtually eliminated toxic effects beyond clinical drugs. This study illustrated the therapeutic potential of AS-TP against TNBC and proposed a promising concept for the development of nucleic acid-based targeted anticancer drugs.

**Supplementary Information:**

The online version contains supplementary material available at 10.1186/s13046-024-03133-5.

## Introduction

Triple-negative breast cancer (TNBC) is a highly malignant carcinoma with negative expression of estrogen receptor (ER), progesterone receptor (PR), and human epidermal growth factor receptor 2 (HER2), accounting for 15–25% of all breast cancers [1]. Studies have identified TNBC as a rapidly progressive and prone to metastasizing breast cancer subtype that was associated with the greatest recurrence and mortality rates, imposing tremendous treatment challenges [2, 3]. Tragically, due to the insensitivity to endocrine therapy and molecular targeted therapy, systemic treatment regimens for TNBC are constrained to cytotoxic chemotherapy, rendering serious toxicities and causing intrinsic drug resistance in metastatic or recurrent patients [4, 5]. Therefore, it is of paramount significance and urgency to explore potential targets for TNBC and conduct effective targeted therapy.

An emerging and high-potential approach is the application of nucleic acid aptamer (NAA)-based tumor targeting systems, incorporating a physical-chemical response to external stimuli, such as pH, enzymes, light or magnetism, to facilitate the precise release of drugs for the proactive treatment of malignant tumors [6–8]. NAAs, as single-stranded DNA or RNA oligonucleotide fragments, constitute a specific three-dimensional spatial configuration through folding with secondary and tertiary structures, which could bind to different target molecules with high specificity and affinity by van der Waals forces, hydrogen bonds, electrostatic interactions, or base stacking forces, etc. [9, 10]. Due to their superiorities in comparison with antibodies, for instance, high stability, non-immunogenicity, rapid targeted cell penetration, convenience of synthesis and modification, and extensive availability of target molecules, NAAs present a promising application in cancer diagnosis and targeted drug delivery system development [11, 12].

Nucleolin protein (NuP) is a ubiquitous, versatile, and highly conserved protein that binds RNA, which has been linked to numerous biological processes, including as ribosome biosynthesis, cycle cell division and survival, oncogenesis, and tumor development [13, 14]. Unlike its modest expression in normal tissues, NuP is expressed in a wide range of solid tumors, including stomach, colon, and breast malignancies [15, 16]. Significantly, NuP demonstrated distinct expression patterns between normal and cancer cells, with nuclear localization predominantly present in normal cells, while, as a shuttling protein between the cell membrane, nucleus and cytoplasm, NuP is abundantly expressed on the cancer cell membrane [17]. As one of the most thoroughly investigated tumor-targeting aptamers, AS1411, a guanine-rich DNA aptamer, has been demonstrated in phase II trials for the treatment of metastatic renal cell carcinoma to possess outstanding tumor-targeting properties and exceptional safety, binding with high specificity and affinity to the NuP, which is overexpressed on the membrane surface in various tumor cells, and recognized as a potential biomarker for tumor diagnosis and a target for tumor therapy [18, 19]. Therefore, AS1411 represents a prospective tumor-targeting vehicle to deliver cytotoxic drugs for the treatment of malignancies in which NuP is located on the cell surface.

The upregulation of glycolysis in tumor tissues due to hypoxia causes lactic acid production, as well as tumor overexpression of carbonic anhydrase (CA) catalyzes the reaction between CO_2_ and H_2_O, which mediates the increasing proton quantity in the tumor microenvironment [20]. In comparison with normal tissue (pH = 7.4), the pH values of extracellular environment and intracellular matrix of tumor tissues were 6.2–6.8 and 4.5–6.5, respectively [21]. Among the currently FDA-approved antibody drug conjugates (ADCs), Mylotarg and Besponsa, which comprise the pH-sensitive hydrazone linkers derived from amino groups in drugs, target relapsed CD33 + acute myeloid leukemia (AML) and CD22 + B-cell acute lymphoblastic leukemia (B-ALL), respectively [22, 23]. In contrast, the traditional pH-sensitive linkers derived from hydroxyl groups, such as acetals, vinyl ether linkers, ester linkers, etc., suffered from poor responsiveness, challenging synthesis, and narrow adaptability, resulting in sluggish progress in the exploitation of hydroxyl groups as control switches for smart drugs [24, 25]. Therefore, the design and development of linkers derived from hydroxyl groups with an intelligently controllable pattern is of extraordinary significance and potential interest in ADCs, aptamer drug conjugates (ApDCs), peptide drug conjugates (PDCs), and nanomedicines.

Triptolide (TP), considered as one of the most potential natural products, exhibits highly effective and widespread antitumor capabilities, and provides stronger anticancer activity than traditional anticancer drugs such as paclitaxel, doxorubicin and cisplatin, especially against TNBC and pancreatic cancer [26, 27]. However, its narrow therapeutic window and inability to accurately discriminate between normal and tumor cells leads to inevitable side effects to normal tissues [28, 29]. An optimistic situation is that the 14-hydroxyl group is the critical bioactive site and the most handily modified functional group [30, 31].

Consequently, we devised an innovative pH-hypersensitive linker to construct an ApDC (AS1411-triptolide conjugate, AS-TP), which possessed excellent water solubility and an exceptional sensitivity to tumor cell recognition and response. And AS1411 modification significantly improved TP’s specificity and affinity for NuP, facilitated the conjugated TP endocytosis by macropinocytosis in a NuP expression-dependent manner and transported into the lysosome, where it instigated the breakage of the acetal ester linker to trigger the release of the original TP. In the human TNBC xenograft model, AS-TP selectively accumulated in tumor tissues to exert antitumor effects, and eventually suppressed breast cancer growth without apparent toxic effects on normal viscera beyond clinical phase III and first-line drug efficacy.

## Results

### Pan-cancer analysis of NCL and targeting properties of the nucleic acid aptamer AS1411 towards NuP

**Fig. 1:**
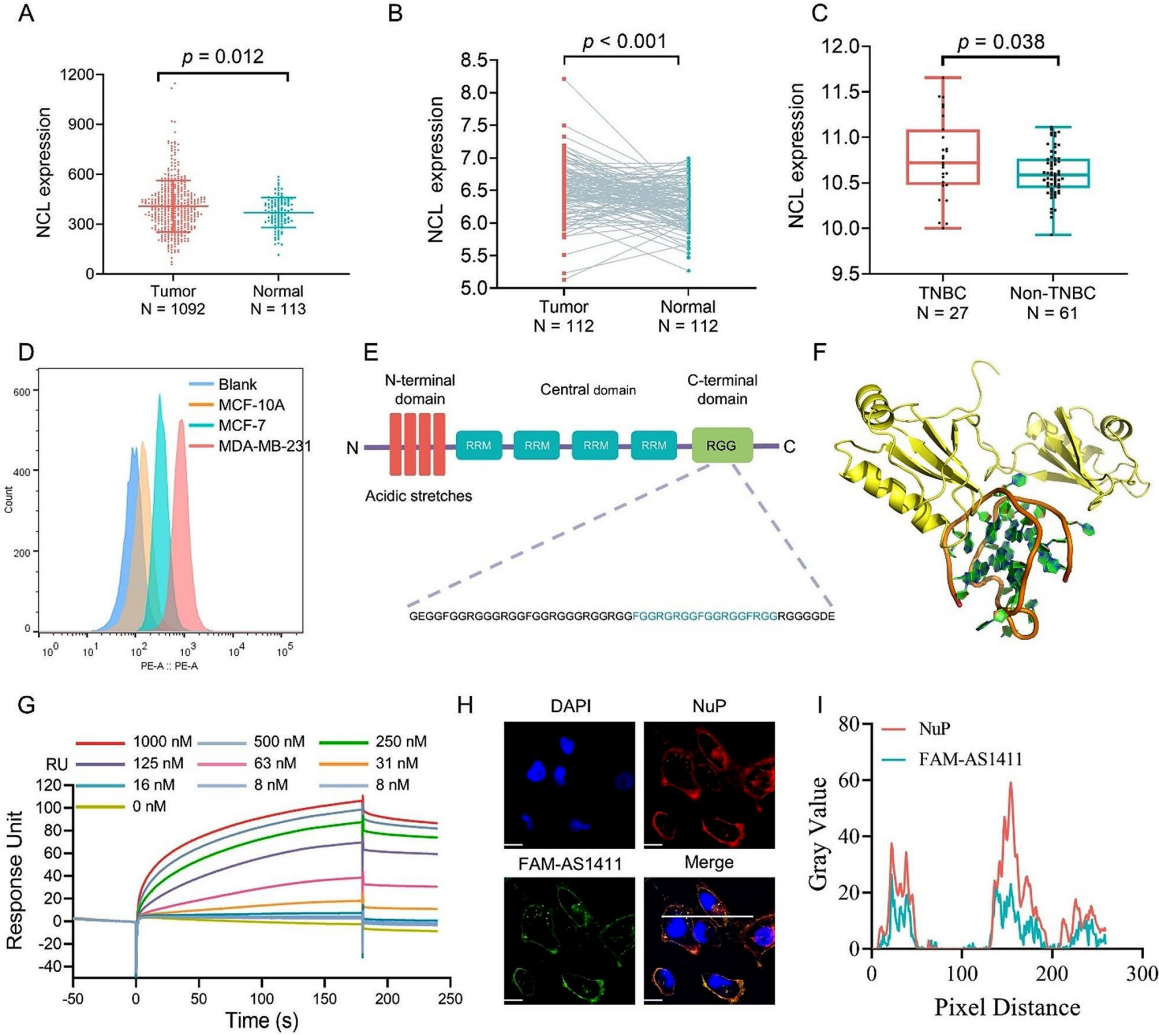
Pan-cancer analysis of NCL and targeting properties of the nucleic acid aptamer AS1411 towards NuP. (**A**) Pan-cancer expression of NCL between BRCA tissues from TCGA database and normal tissues from TCGA and GTEx database. (**B**) Pan-cancer differential expression of NCL in paired tumor and adjacent normal tissues in BRCA from TCGA database. (**C**) Pan-cancer differential expression of NCL between TNBC and non-TNBC tissues from GEO database. (**D**) The expression of NuP on the cell membrane surface of three cell lines: MDA-MB-231, MCF-7 and MCF-10 A. (**E**) Domain structure of NuP (RRM: RNA recognition motif; RGG: glycine–arginine-rich domain). (**F**) Molecular dynamic simulation for AS1411-NuP interaction prediction (golden: RGG-rich domain of NuP; brown: AS1411). (**G**) The affinity of AS1411 to NuP. The curves were adapted with a 1:1 Langmuir fitting model, and the binding kinetic constants were determined from the fitted curves. (**H**, **I**) Co-localization of FAM-AS1411 with NuP on the surface of MDA-MB-231 cell membrane. (NuP: red; FAM-labeled drugs: green; cell nucleus: blue). Bar = 10 μm. Magnification: 600X. Quantification of co-localization was analyzed by Image J

To investigate the aberrant expression of NuP in breast cancer, a harmonised standardised pan-cancer dataset was downloaded from the UCSC (https://xenabrowser.net/) database as follows: TCGA BRCA (T = 1092, *N* = 113), from which the expression of the ENSG00000115053 (NCL) was further extracted from the specimen data, revealing that NCL was dramatically up-regulated in breast cancer (Fig. 1A). For paired tumor and normal tissues in TCGA pan-cancer, NCL was expressed at high levels in BRCA (Fig. 1B). To further explore the clinical properties of NuP, the relationship between tumor typing and gene expression was identified by analysis of the GEO: GSE20713 independent dataset, which uncovered that the expression of the NCL was elevated in TNBC compared to other breast cancers (Fig. 1C). To quantitatively detect only NuP on various cell membranes, we rigorously managed the incubation time and adjusted the cellular immunofluorescence assay. Subsequently, the high level of NuP expression on the surface of the TNBC cell line MDA-MB-231 and the presence of NuP receptors on the membrane surface of the non-TNBC cell line MCF-7 were observed by laser confocal microscopy, whereas the appearance of NuP was almost undetectable on the surface of the normal breast cell line MCF-10A, indicating that aberrant expression of NuP developed in breast cancer cells (Figure S1). Moreover, flow cytometry analysis of receptor expression revealed significantly higher expression levels of NuP in TNBC cell lines MDA-MB-231, MDA-MB-468 and 4T1, compared to non-TNBC cell line MCF-7 and normal breast cell line MCF-10A (Fig. 1D and Figure S2-S3). NuP comprises multiple functional domains, including an amino-terminal charged region, a central region consisting of four RNA-binding domains, and a glycine/arginine-rich (RGG) region at the carboxyl terminus (Fig. 1E) [32]. Previous studies have illustrated that AS1411 could target the NuP and disrupt the combination of NuP with the Kif5a motility complex [33]. The molecular dynamics simulation of the binding properties of NuP and AS1411 indicated that the visual interaction between AS1411 and the RGG structural domain of NuP was present (Fig. 1F). Additionally, surface plasmon resonance (SPR) kinetic experimental data fitting for the interaction between AS1411 and recombinant NuP disclosed that AS1411 bound to recombinant NuP in a 1:1 pattern with an equilibrium dissociation constant of 3.77 nM (Fig. 1G). Further examination of NuP interaction with AS1411 in MDA-MB-231 cells demonstrated that NuP colocalized virtually exclusively with AS1411 in TNBC cells (Fig. 1H, I).

### Preparation and characterization of aptamer-triptolide conjugates

**Fig. 2:**
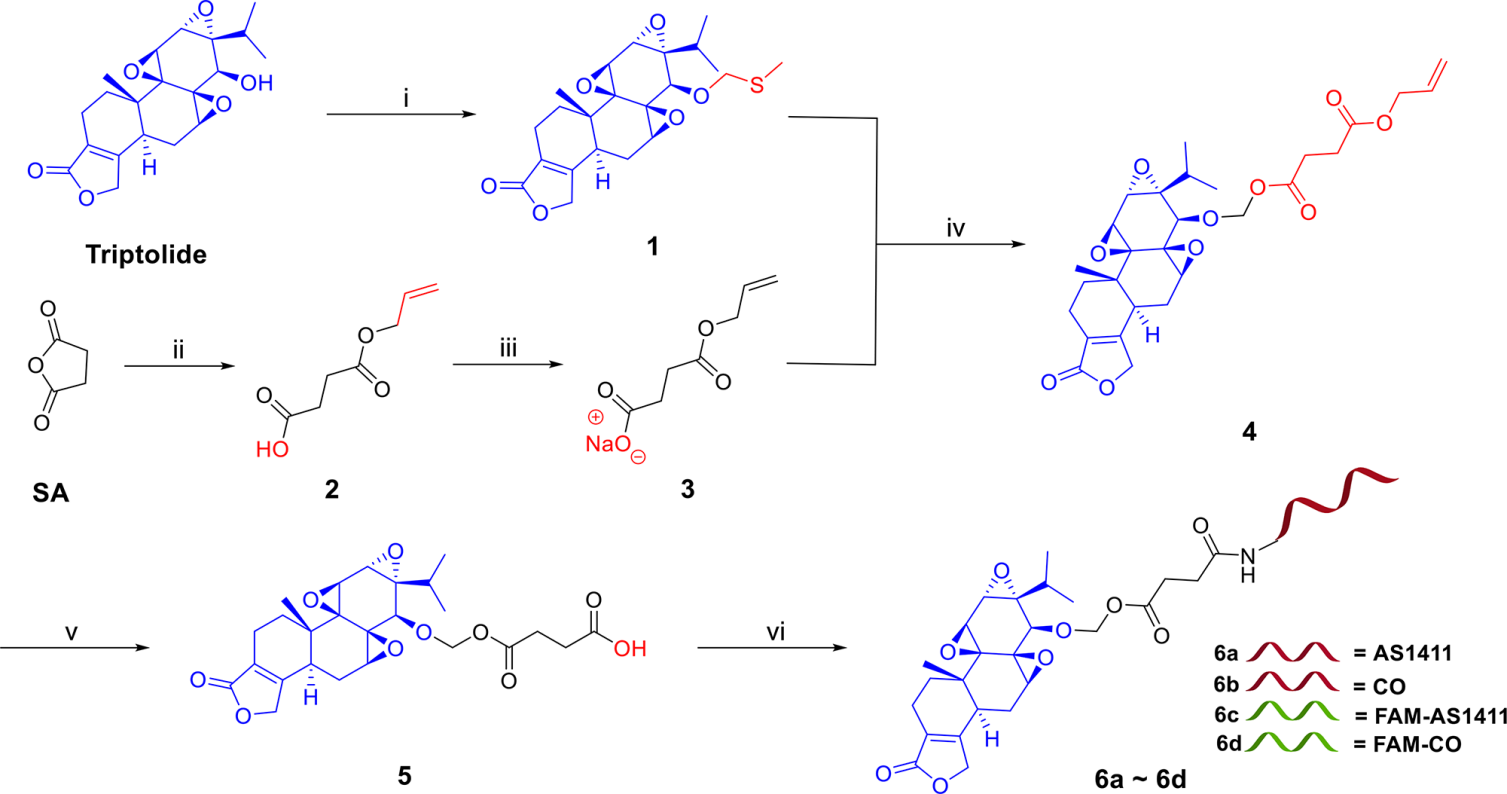
Synthesis of aptamer-triptolide conjugates with the acetal ester linker. Note for reagents and conditions: (i) AcOH, Ac_2_O, DMSO; (ii) allyl alcohol, DMAP, toluene, 40 °C; (iii) NaHCO_3_, acetone; (iv) SO_2_Cl_2_, 3, 15-crown-5, CH_2_Cl_2_; (v) morpholine, Pd(PPh_3_)_4_, THF; (vi) Sulfo-NHS, EDCI, aptamer, dd-H_2_O, DMF, 0.5 M Na_2_CO_3_/NaHCO_3_. DMAP = 4-Dimethylaminopyridine, DMSO = Dimethyl sulfoxide, THF = Tetrahydrofuran, Sulfo-NHS = *N*-hydroxysulfosuccinimide sodium salt, EDCI = 1-Ethyl-3-(3-dimethyllaminopropyl) carbodiimide hydrochloride, DMF = Dimethylformamide, AS1411 = Nucleolin aptamer, CO = Cytosine-rich oligonucleotide, FAM-AS1411 = Fluorescein amidate labeled nucleolin aptamer, FAM-CO = Fluorescein amidate labeled cytosine-rich oligonucleotide

Synthesis of AS-TP commenced with the blockade of the essential bioactive functional group on triptolide (Fig. 2). The 14-hydroxyl group on TP was reacted with DMSO catalyzed by acetic acid to form the semithiocarbital derivative 1. Simultaneously, the preparation of the acid sensitive linker fragment via the esterification reaction between the commercially available succinic anhydride (SA) and allyl alcohol in the presence of DMAP promptly provided the intermediate 2, which was converted to the corresponding sodium carboxylate 3 in a good yield. Without further purification, the carboxyl anion in 3 with the assistance of sulfuryl chloride and 15-crown-5 assaulted the sulfur atom on 1, affording 4 with a novel acid-labile acetal ester. Treating 4 with Pd(PPh_3_)_4_ and morpholine quickly removed the allyl-protecting group and give rise to 5, which was then conjugated with AS1411 in the presence of sulfo-NHS and EDCI to afford AS-TP (6a). CO-TP (6b), FAM-AS-TP (fluorescein amidate-labeled AS-TP, 6c), and FAM-CO-TP (fluorescein amidate-labeled CO-TP, 6d) were prepared as the described method above by utilizing a control DNA aptamer CO (cytosine-rich oligonucleotide), FAM-labeled AS1411 and FAM-labeled CO, respectively, for conjugation at the last step. The structures of key intermediates were confirmed by [1]H-NMR and [13]C-NMR spectroscopy and high-resolution mass spectrometry (HRMS) (Figure S4-S8). Conjugates were characterized by HPLC and mass spectrometry (Figure S9-S12), and the serial number of DNA is same to that in Table S1.

### Release mechanism and superiority of the designed linker

**Fig. 3:**
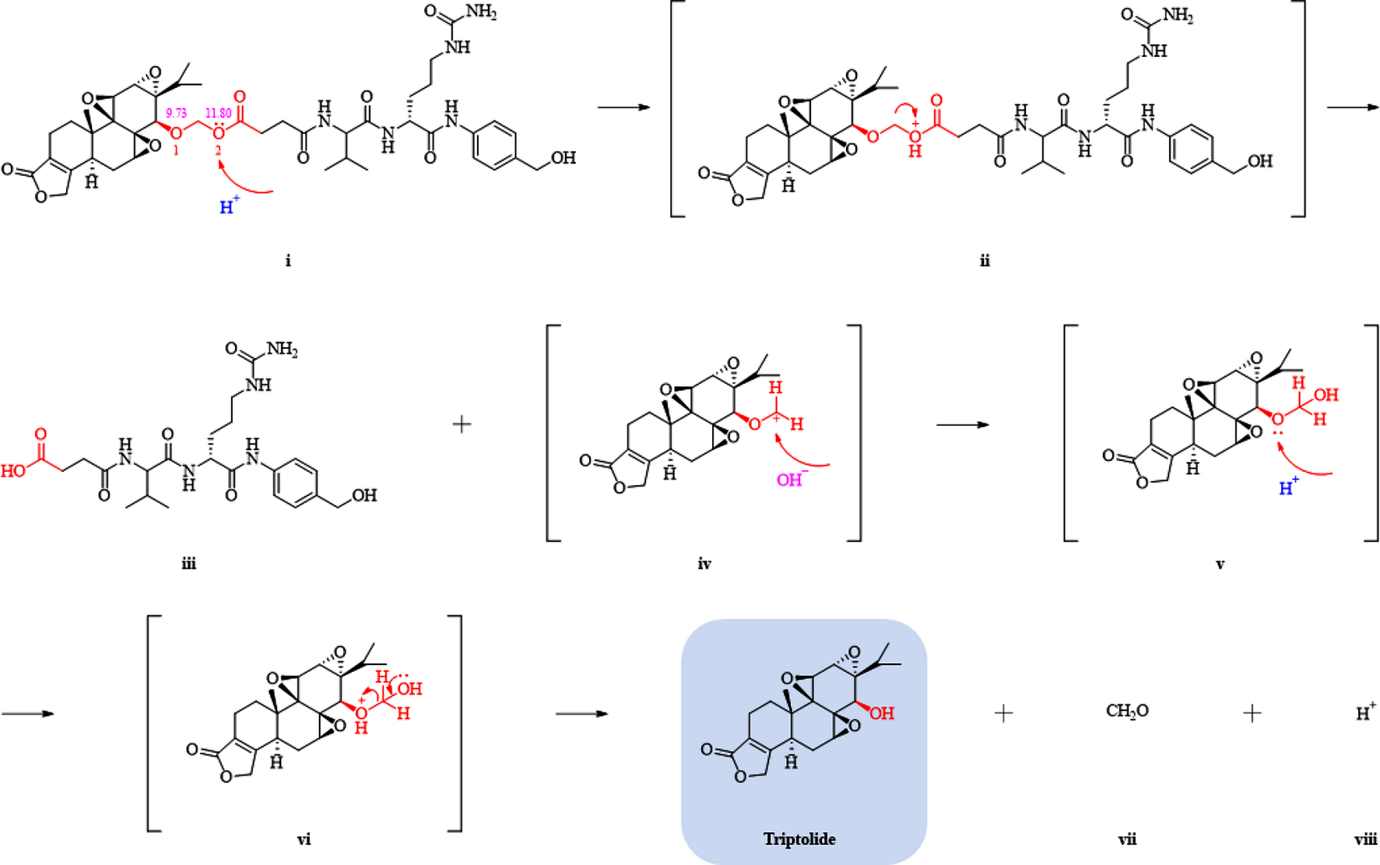
Schematic diagram of fracture mechanism for dipeptide bond-triptolide conjugate under acidic condition

Importantly, for a thorough insight into the properties of the designed linker and expand its application, a dipeptide consisting of valine and citrulline was conjugated to TP by the above linker to form a facile PDC (Figure S13). The structure of the main intermediate was confirmed by [1]H-NMR, [13]C-NMR and high-resolution mass spectrometry (HRMS) (Figure S14-S18). Under weak acidic conditions, the PDC was detected to release TP in a time-dependent manner by real-time LCMS monitor and NMR identification (Figure S19-S20). Consequently, the mechanism of the linker rapture and TP release from conjugates was postulated from the decomposed fragment compounds (Fig. 3). As detected by Marvin Sketch (20.8, ChemAxon, Budapest, Hungary), the acetal ester bond possesses a relatively high electronegativity, with oxygen atoms 1 and 2 exhibiting electronegativities of 9.73 and 11.80, respectively. Due to the intense electronegativity of the acetal ester bond, electrons preferentially migrated towards the ester bond, rendering the hydrogen ion more prone to attacking the electronegative oxygen atom #2 in the acetal ester bond (i) and forming the oxonium salt as a coordination bond (ii). The generated O^+^ featured a robust electron-withdrawing capacity, while in concert with the strong electron-withdrawing potential of the carbonyl group, the C-O bond fractured to afford the corresponding dipeptide-bonded derivatives (iii) and the carbon-positive ion transition state (iv). The extremely unstable carbon positive ion preferred to combine with environmental hydroxide ions to shape the hemiacetal structure, which is, however, wobbly in acidic aqueous solutions, and the oxygen atom at the C14 position of TP constructed a new oxonium salt in association with the hydrogen ion (v). Ultimately, following electron transfer at the distal hydroxyl group, vi was decomposed into TP, formaldehyde (vii), and hydrogen ions (viii).

**Fig. 4:**
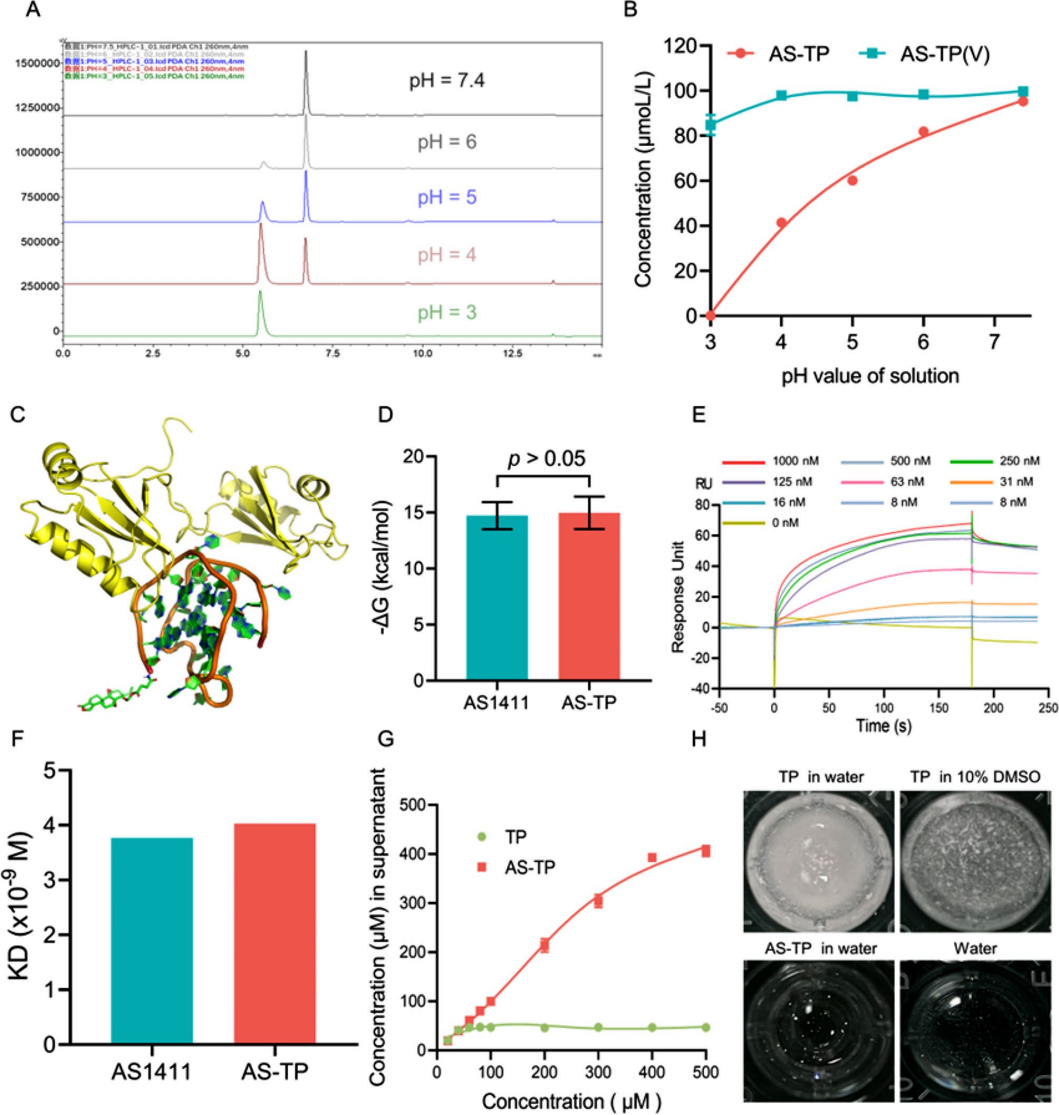
Characterization of physical and chemical properties of AS-TP. (**A**) The cleavage of the acetal ester linker in AS-TP was determined by HPLC at different pH (pH = 3, 4, 5, 6 and 7.4). (**B**) The release of AS-TP under different pH conditions was detected by LC-MS. (**C**) The interaction between AS-TP and NuP after molecular dynamic simulation (golden: RGG-rich domain of NuP; brown: AS1411; green: TP). (**D**) The Gibbs free energy between AS1411 or AS-TP and NuP has been investigated by molecular dynamics simulations. (**E**) The affinity of AS-TP to NuP. (**F**) The binding kinetic constants between AS1411 or AS-TP and NuP were determined from the fitted curves. The curves were adapted with a 1:1 Langmuir fitting model. (**G**) The solubility of TP and AS-TP in water was determined by HPLC. (**H**) The dissolution profiles of 2 µmoL of TP and AS-TP in 200 µL different solutions

To validate the superiority of the innovative acid-sensitive linker, the AS-TP(V) (6e) comprising the conventional acid-labile vinyl ester linker derived from the hydroxyl in TP was also synthesized, and [1]H-NMR and [13]C-NMR spectra of intermediates is shown in the supplementary materials (Figure S21-S24). Conjugates were characterized by HPLC and mass spectrometry (Figure S25). Subsequently, the release of TP from AS-TP and AS-TP(V) at different pH conditions was determined by HPLC. With the increase of acidic intensity in PBS, the absorption peak area of AS-TP gradually diminished, while that of AS1411 showed an increase (Fig. 4A). For AS-TP(V), the appearance of the AS1411 absorption peak was only monitored at pH = 3 (Figure S26), additionally, the data statistics discovered that the release rate of AS-TP was 57% at pH = 4 where the tumor microenvironment was comparable, although no fracture product peaks of AS-TP(V) were being observed (Fig. 4B). The comparative results concisely demonstrated that the acetal ester linker guaranteed the dissociation of AS-TP in acidic environment of the lysosome (pH = 4.5–5.5) to release the intact TP and possessed a remarkable acid sensitivity over the vinyl ether linker.

### NuP targeting, water solubility and serum stability of AS-TP

To plainly identify whether TP conjugation impacted the binding affinity of AS1411 to its target NuP, molecular dynamic simulation and SPR were employed to investigate the affinity after modification of AS1411 to NuP. In the simulation model, we observed that AS-TP interacted with the RNA-binding domain, and in quantitative terms, the binding free energies calculated on docking of AS1411 and AS-TP with NuP were not statistically significantly different (Fig. 4C, D). Moreover, AS-TP revealed high binding affinity to human recombinant NuP over a broad concentration range by SPR, exhibiting rapid association and slow dissociation with KD values of 4.03 nM, which indicated that no remarkable variation in the affinity for AS-TP and AS1411 to NuP was observed (Fig. 4E, F). In contrast, the negative controls CO-TP exhibited a minimal degree of association capacity with NuP, moreover, the absence of affinity between TP and NuP implied that TP possessed no targeting to NuP (Figure S27). These results indicated that AS1411 apparently associates with NuP, and TP modification has no implications on the binding affinity of AS1411 to NuP, moreover, AS1411 could confer to a great extent TP targeting property to NuP. The water solubility determined for the TP was 47 µM, while AS-TP was virtually water-soluble, obviously displaying a solubility of more than 400 µM and the performance is significantly better than free TP under similar conditions (Fig. 4G, H). ​Considering that adequate serum tolerance is a prerequisite and critical factor for successful in vivo application of these ApDCs, the stability of AS-TP was evaluated next. The HPLC analysis of AS-TP incubated in 50% PBS-buffered human serum at 37 °C revealed that AS-TP exhibited excellent stability even after 24 h incubation and consistently present as an intact conjugate, indicating that the acetal ester linker could ensure the stability of AS-TP in serum without decomposition or release (Figure S28).

### Cytotoxicity and tumor targeting of AS-TP against TNBC

**Fig. 5:**
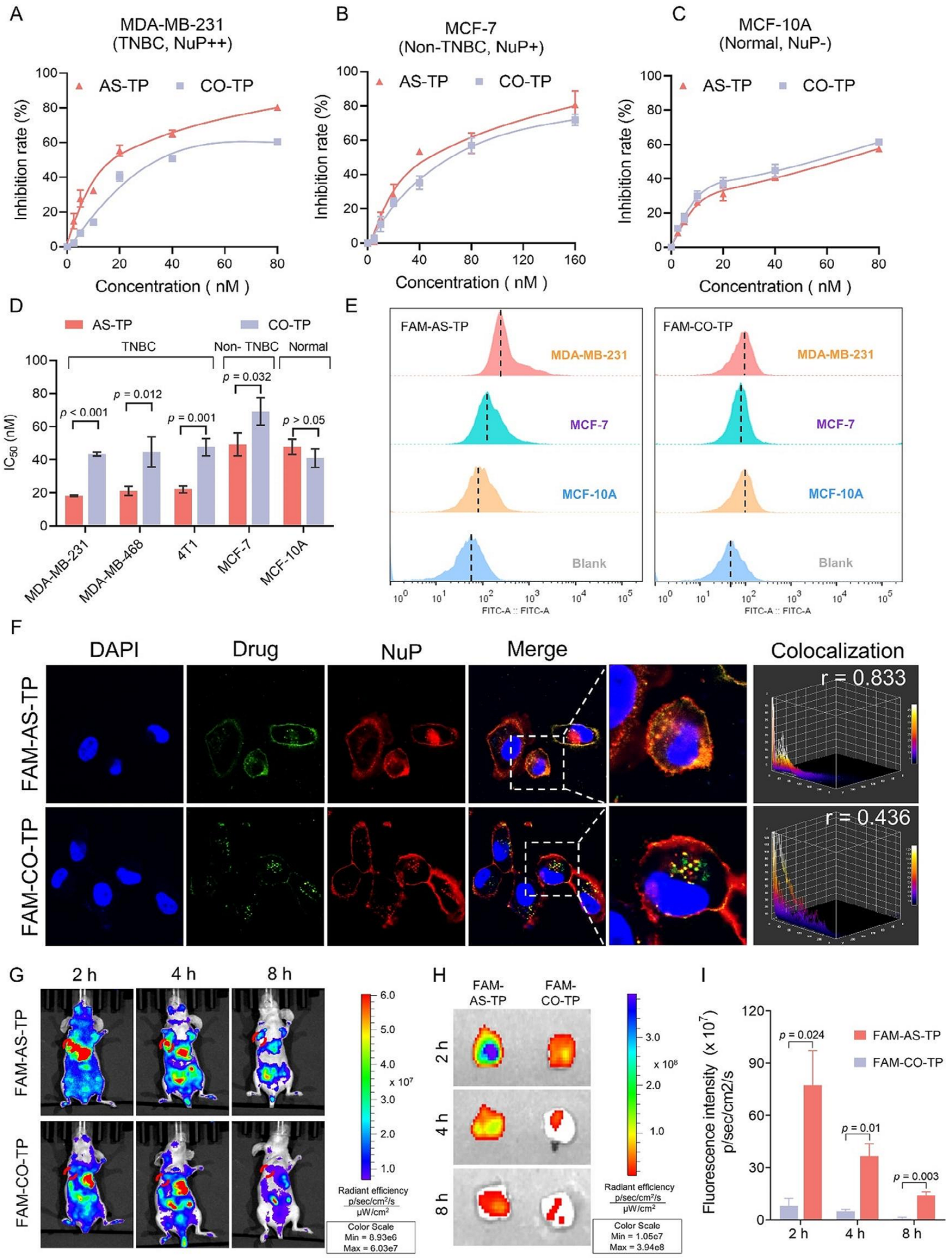
Cytotoxicity and tumor targeting of AS-TP. (**A**-**C**) MTT proliferation assay was used to determine the effect of the AS-TP and CO-TP on the growth of cell lines with different NuP expression. (**D**) IC_50_ values of AS-TP and CO-TP on TNBC cell lines MDA-MB-231, MDA-MB-468, 4T1, and non-TNBC cell line MCF-7, and normal cell line MCF-10 A. (**E**) The uptake of FAM-AS-TP and FAM-CO-TP in MDA-MB-231, MCF-7 and MCF-10 A cell lines by flow cytometry assessment. (**F**) Co-localization of FAM-AS-TP and FAM-CO-TP with NuP on the surface of MDA-MB-231 cell membrane. (NuP: red; FAM-labeled drugs: green; cell nucleus: blue). Bar = 10 μm. Magnification: 600X. Quantification of co-localization was analyzed by Image J. (**G**) In vivo the dynamic distribution of FAM-CO-TP and FAM-AS-TP in the MDA-MB-231 mice tumor model after different periods of tail vein injection. (**H**) The distribution of FAM-CO-TP and FAM-AS-TP in tumor tissues after different times of intravenous injection by biophotonic imaging. (**I**) Semiquantitative analysis of fluorescence intensity in tumor tissues in vivo at different administration times

To investigate whether the cytotoxic effect of AS-TP was dependent on NuP expression, the MTT method was employed to assess the impact of AS-TP and CO-TP on various cell lines. The results depicted in the Fig. 5A-C and Figure S29 indicate that the cytotoxicity of AS-TP and CO-TP is concentration-dependent across different cell lines. Specifically, the IC_50_ values of AS-TP for MDA-MB-231, MDA-MB-468, and 4T1 cells were 18.31 ± 0.42 nM, 21.23 ± 1.61 nM, and 22.09 ± 1.21 nM, respectively, whereas the IC_50_ values of CO-TP were 43.52 ± 1.12 nM, 44.80 ± 9.10 nM, and 47.58 ± 5.10 nM, respectively. These findings demonstrated the superior cytotoxic effect of AS-TP compared to CO-TP on triple-negative breast cancer cells (Fig. 5D). Moreover, AS-TP exhibited slightly enhanced anti-tumor activity compared to CO-TP in the non-triple-negative breast cancer cell line MCF-7(*p* = 0.032), while both agents demonstrated comparable cytotoxicity in the normal breast cancer cell line MCF-10 A (*p* > 0.05) (Fig. 5D). The uptake efficiency of AS-TP and CO-TP in various cell lines was determined by measuring the fluorescence intensities. Notably, FAM-AS-TP exhibited higher internalization in TNBC cells MDA-MB-468 and 4T1 compared to FAM-labeled CO-TP (*p* < 0.001) (Figure S30, S31). Besides, as compared with fluorescence intensities in MCF-7 cells, MDA-MB-231 cells pointed the maximum level to a median of 296.3 ± 35.5. Surprisingly, the comparison revealed that MCF-10 A cells barely uptake FAM-AS-TP during this timeframe (Fig. 5E and Figure S32). In contrast, the uptake of FAM-CO-TP did not vary dramatically in all three cell lines (*p* > 0.05), implicating that FAM-AS-TP could be specifically recognized and internalized by NuP-overexpressed cells with a positive correlation to NuP expression, out of which MDA-MB-231 dominated decidedly specific recognition and uptake of FAM-AS-TP (Fig. 5E and Figure S32). To examine whether AS1411 modification mediated the TP uptake and cytotoxicity, further competitive uptake inhibition assays revealed that AS1411 lessened the uptake of FAM-AS-TP by MDA-MB-231 in the presence of non-toxic concentrations of AS1411 (Figure S33). Meanwhile, AS1411 notably diminished the lethal effect of AS-TP on TNBC cells (Figure S34). Furthermore, we performed confocal imaging to validate the colocalization of FAM-labeled AS-TP with NuP in MDA-MB-231 cells, a representative TNBC cell line with high expression of NuP. Immunofluorescence staining revealed that FAM-AS-TP displayed intense colocalization with NuP with the Pearson correlation coefficients *r* = 0.83, rendering bright yellow fluorescence (Fig. 5F). Nevertheless, in comparison, FAM-CO-TP embodied no noticeable colocalization with NuP, with the r value below 0.5 (Fig. 5F). Additionally, laser confocal displayed that FAM-AS-TP and NuP presented favorable codetermination effect in the TNBC cell lines MDA-MB-468 and 4T1 as well (Figure S35). To investigate the in vivo tumor targeting of AS-TP, the xenograft mice model of human malignant TNBC was established. Satisfactorily, FAM-AS-TP was further abundantly distributed in tumor locations, whose fluorescence intensity was 7.9 folds greater than that of FAM-CO-TP at the same moment, and the fluorescence intensity of the FAM-AS-TP group at the tumor location remained remarkably higher than that of the FAM-CO-TP group with the increasing of time (*p* < 0.01) (Fig. 5G and S36). Subsequently, for more accurate validation of the tumor targeting of AS-TP in vivo, we performed ex vivo imaging of the tumor. Encouragingly, FAM-AS-TP exhibited notable tumor tissue retention, with approximately 11 times as much fluorescence intensity as FAM-CO-TP in tumor tissue. In addition, an intense fluorescence of FAM-AS-TP was still observed in tumor tissues, while the fluorescence of FAM-CO-TP gradually disappeared after 8 h administration (Fig. 5H, I). The above outcomes supported that FAM-AS-TP displayed distinct tumor specificity and mighty targeting ability, achieving efficient accumulation and prolonged retention in tumor regions.

### Absorption process and endocytosis pathway of AS-TP by TNBC cells

**Fig. 6:**
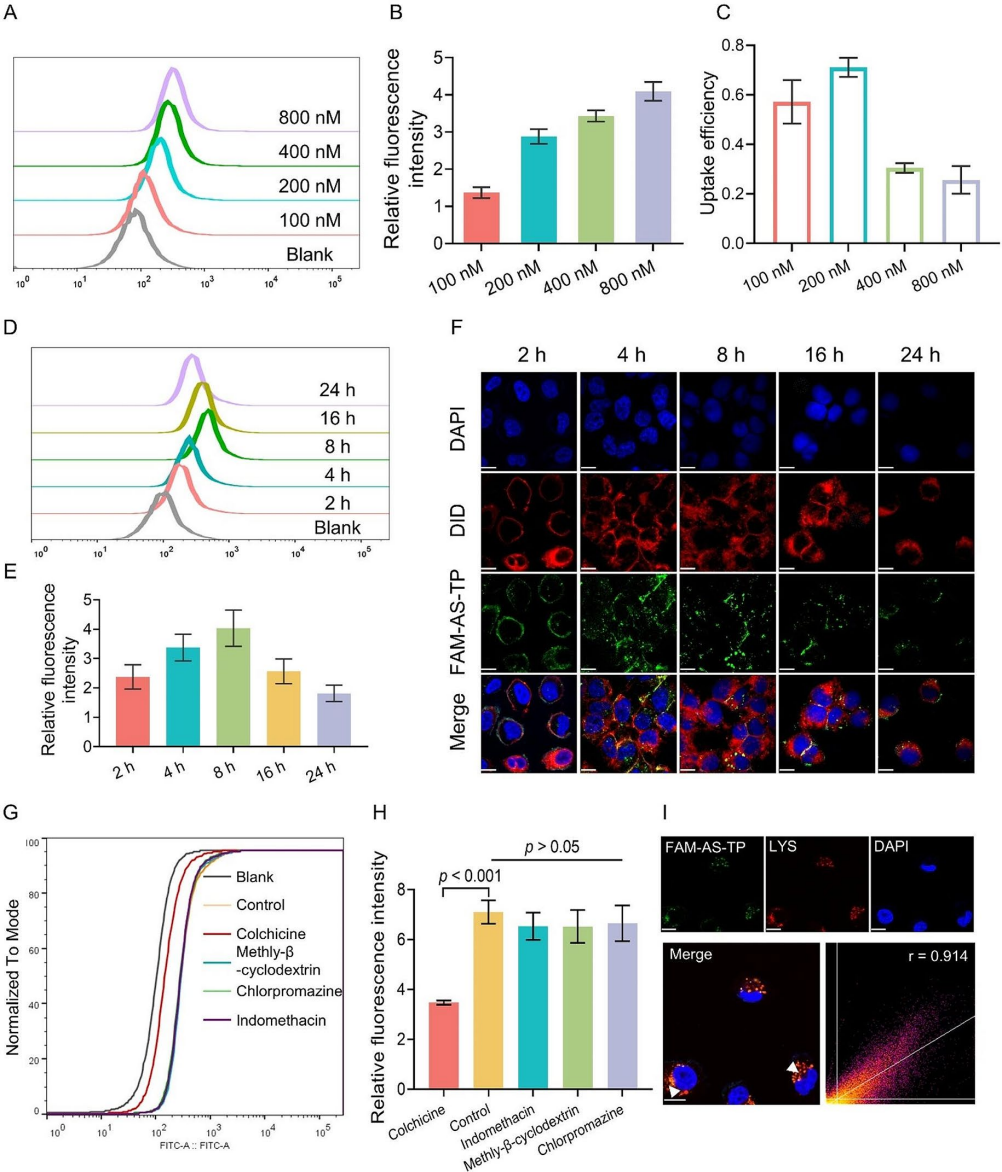
Absorption process and endocytosis pathway of AS-TP by TNBC cells. (**A**-**C**) The uptake and uptake efficiency of MDA-MB-231 cells to different concentrations of FAM-AS-TP. (**D**, **E**) The uptake and uptake efficiency of FAM-AS-TP by MDA-MB-231 cells at different coincubation time-points. (**F**) The uptake process of FAM-AS-TP by MDA-MB-231 cells. (Membrane: red; FAM-AS-TP: green; cell nucleus: blue). Bar = 10 μm. Magnification: 600X. **G**-**H**) The uptake of FAM-AS-TP by MDA-MB-231 cells after preincubation with different endocytic pathway inhibitors for 2 h. (**I**) Representative images of laser confocal microscopy showing co-localization (white arrows) of FAM-AS, FAM-AS-TP and FAM-CO-TP (green) with lysosomal marker (red). Cell nuclei were double-stained with DAPI (blue) and the co-localization coefficients of drugs and lysosomes were analyzed by Image J. Bar = 10 μm. Magnification: 600X

The research indicated that NuP exerted a critical role in the uptake of AS-TP by TNBC cells. Subsequently, the analysis of fluorescence intensity was conducted to determine the influence of administration concentration and time on the AS-TP uptake by MDA-MB-231 cells. The uptake of FAM-AS-TP in MDA-MB-231 cells presented a prominent concentration-dependent increase, with the optimal uptake efficiency at a concentration of 200 nM (Fig. 6A-C). Meanwhile, the uptake of FAM-AS-TP proceeded to increase upon the administration time by MDA-MB-231 cells with the maximum intake occurring at 8 h incubation time, whereas the intracellular fluorescence intensity commenced to decline after 8 h (Fig. 6D, E). Additionally, the internalization process analyzed by confocal microscopy revealed that FAM-AS-TP entered the MDA-MB-231 cells (4 h) and induced the distortion of the membrane (8 h), which indicated the beginning of apoptosis with eventual attachment to the nuclear membrane (Fig. 6F). To thoroughly explore the internalization mechanism of AS-TP, MDA-MB-231 cells were pretreated with different endocytic pathway inhibitors for 2 h and co-incubated for 4 h with FAM-AS-TP. The uptake of FAM-AS-TP was detected to be dramatically suppressed by colchicine (a micropinocytosis pathway inhibitor), at a level of approximately two-fifths compared to the control group (Fig. 6G, H). Whereas the pretreatment with chlorpromazine (clathrin-mediated endocytosis inhibitor), indomethacin (caveolin-mediated endocytosis inhibitor), or methyl-β-cyclodextrin (cholesterol-mediated endocytosis inhibitor) resulted in no statistically significant diminution in uptake (*p* > 0.05 vs. control), indicating that FAM-AS-TP was primarily taken up by MDA-MB-231 cells via micropinocytosis. Furthermore, the confocal microscopy analysis revealed a reduction in FAM-AS-TP uptake by colchicine-pretreated MDA-MB-231 cells and independence from the other three inhibitors, consistent with the above assay results (Figure S37). Cellular internalization of AS1411 targeting has been reported to eventually accumulate in lysosomes[9a]. Herein, the lysosomal fluorescent probe was co-incubated with MDA-MB-231 cells, and we determined the organelle distribution of the compounds by measuring the colocalization of FAM-AS-TP with lysosomes. Following micropinocytosis, FAM-AS-TP predominantly colocalized with lysosomes in MDA-MB-231 cells, according to a Pearson correlation coefficient of 0.914 (Fig. 6I). Collectively, it was demonstrated that MDA-MB-231 cells underwent endocytosis of FAM-AS-TP via the micropinocytosis pathway and transported it into lysosomes, where the acidic environment facilitated the rupture of acid-sensitive linker in FAM-AS-TP and efficient release of TP.

### Anti-TNBC efficacy of AS-TP in vivo

**Fig. 7:**
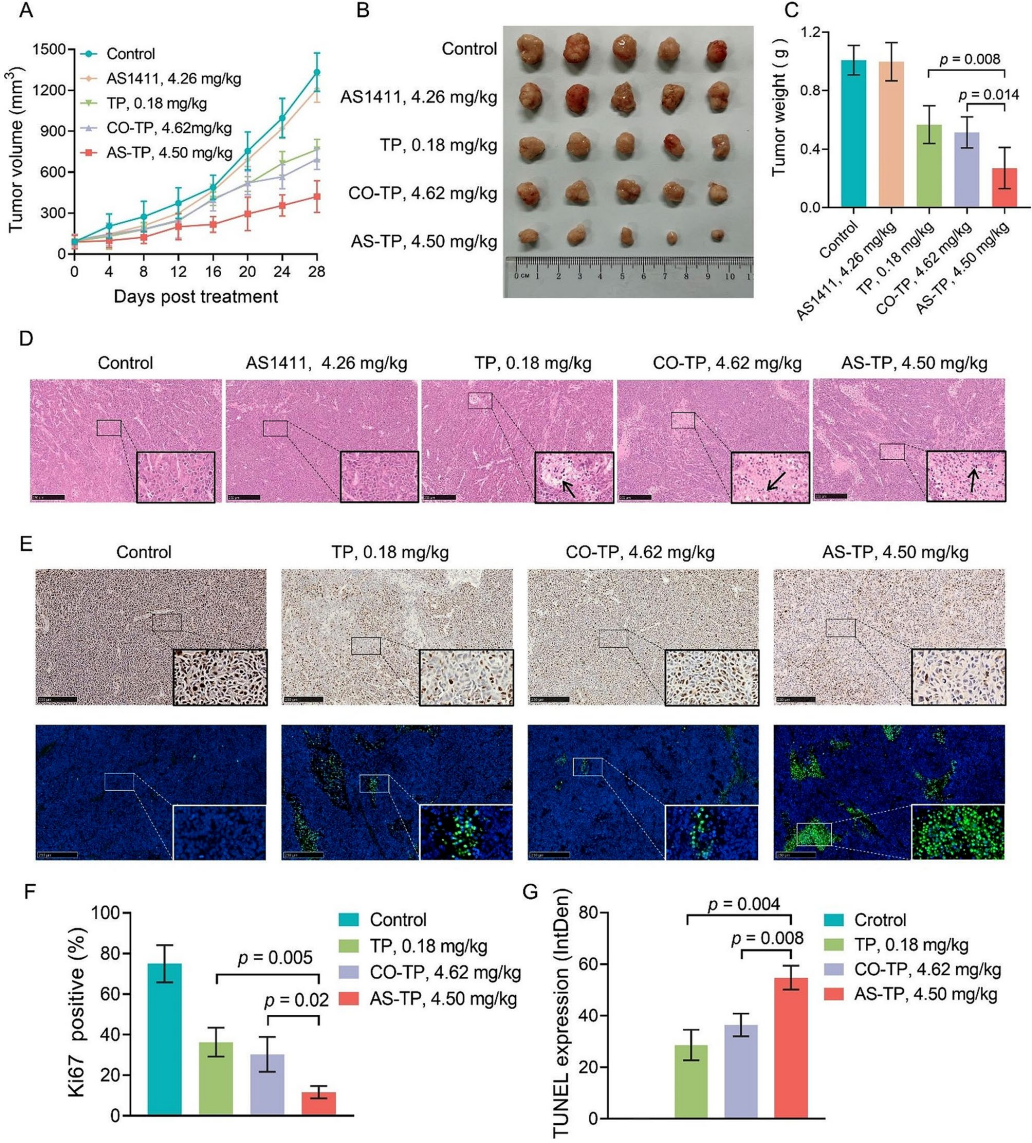
Anti-TNBC efficacy of AS-TP in vivo. (**A**) The changes in tumor volume in xenograft nude mice during treatment after intravenous injection of different drugs. (**B**) Macroscopic observation of the xenograft tumor morphology after 28 days of treatment. (**C**) Tumor weights of xenograft nude mice after the prescribed time of different treatments. (**D**) H&E staining micrograph analysis of tumor tissue sections in each treatment group. (**E**) Immunofluorescent analysis of Ki67 and TUNEL staining in tumor from mice with different treatment (DAPI: blue, TUNEL: green). (**F**) Quantification of Ki67-positive cells in tumor tissues. (**G**) TUNEL positive percentage of tumor tissue measured in tumor tissues. *n* = 5

Proceeding from the superior in vitro antitumor efficacy and in vivo tumor targeting of AS-TP, we next investigated the therapeutic efficacy and safety of AS-TP in the MDA-MB-231 nude mice xenograft model compared with the original TP and CO-TP. Firstly, the tumor-bearing mice were administered with free TP, CO-TP, AS1411, and AS-TP at identical molar concentrations and saline, respectively. After 28 days of treatment, AS-TP, TP, and CO-TP all exhibited remarkable antitumor efficacy (Fig. 7A-C). Concerning tumor therapy, AS-TP-treated mice presented the slowest tumor growth rate, the minimum tumor volume and the lightest tumor weight. The tumor growth inhibition in the AS-TP group was obviously stronger than that in the CO-TP and TP groups, whereas no visible anticancer activity was detected in the AS1411 group (as compared to the control, *p* > 0.05) (Fig. 7A-C). Subsequently, H&E staining was performed to further investigate the tumor suppression efficiency of AS-TP, which revealed different degrees of pathological necrosis such as cell membrane rupture and nuclear lysis were observed in tumor tissues from CO-TP-treated, TP-treated and AS-TP-treated xenografted mice, and the most pronounced pathological damage was found in the internal tumor of the AS-TP-treated group (Fig. 7D). Moreover, the Ki67 staining results additionally displayed the least number of yellow particles in the AS-TP-treated group, whose positive rate was less than 15%, indicating that the tumors in this group proliferated the most slowly and distorted the least malignantly (Fig. 7E and F). Furthermore, the fluorescence intensity of TP-treated, CO-TP-treated and AS-TP-treated groups were gradually strengthened according to the TUNEL staining assay, indicating that the apoptosis rate of tumor cells was progressively elevated, up to 55% in AS-TP-treated group (Fig. 7E, G).

### Antitumor mechanism of AS-TP against MDA-MB-231 cells

**Fig. 8:**
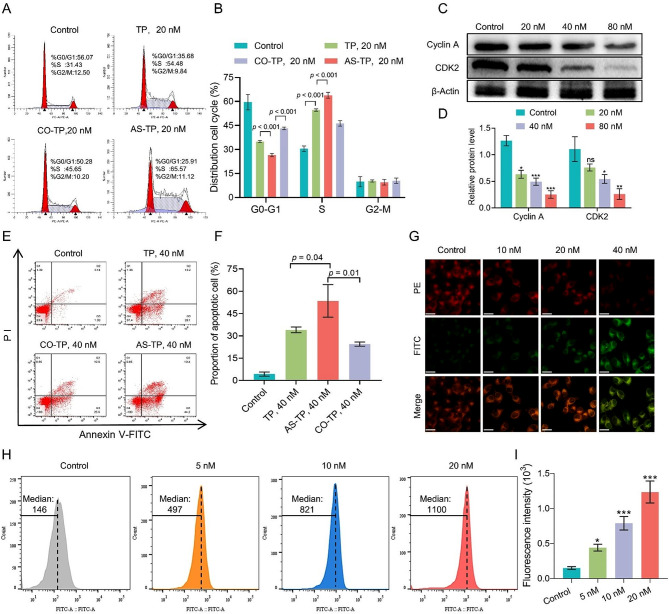
Antitumor mechanism of AS-TP in vitro. (**A**, **B**) Cycle distribution of MDA-MB-231 cells after treatment with TP, CO-TP and AS-TP. (**C**, **D**) The levels of S-phase arrest-related proteins (Cyclin A and CDK2) in MDA-MB-231 cells after the treatment with AS-TP. All protein levels were quantified by standardizing β-Actin. (**E**, **F**) The effects of TP, CO-TP and AS-TP on apoptosis of MDA-MB-231 cells. (**G**) The variation of mitochondrial membrane potential after treatment of MDA-MB-231 cells with different concentrations of AS-TP (PE: depolarization; FITC: polarization. Bar = 10 μm. Magnification: 400X). (**H**, **I**) The accumulation of ROS after 24 h treatment of MDA-MB-231 cells with different concentrations of AS-TP. *n* = 3. Compared to the control group, *p < 0.05, **p < 0.01, ***p < 0.001, ns *p* > 0.05

To explore the potential mechanism of AS-TP against TNBC, we firstly investigated the impacts on the distribution of MDA-MB-231 cell cycle by conjugates and TP with flow cytometry. As shown in Fig. 8A and B, AS-TP and CO-TP arrested cells in S-phase by preventing cellular S-phase DNA replication, in accordance with the cycle arresting effect of TP on MDA-MB-231 cells. Moreover, with the same administration concentration conditions, the capability of AS-TP to induce cycle arrest was dramatically more robust than that of the TP and CO-TP. Meanwhile, Western blot analysis demonstrated that AS-TP downregulated the expression of cyclin A and CDK2, which indicated that AS-TP suppressed tumor proliferation by modulating the expression in MDA-MB-231 cells of cyclin and cyclin-dependent kinase during DNA replication (Fig. 8C, D). Then Annexin V-FITC/PI double staining verified the anti-tumor mechanism of AS-TP after internalization into cells, revealing that conjugates as well as TP induced apoptosis and dramatically augmented the percentage of early apoptotic cells (Fig. 8E, F). The above experimental results confirmed that AS-TP and CO-TP possessed the identical anti-TNBC mechanism as TP. Depolarization of mitochondrial membrane potential and increased generation of intracellular reactive oxygen species (ROS) are hallmarks of early apoptosis [34]. The laser confocal revealed that when AS-TP concentrations increased, the intracellular red fluorescence intensity was weakened, whereas the intensity of green fluorescence was augmented (Fig. 8G). Furthermore, at a dose of 40 nM, AS-TP was shown to enhance the fraction of mitochondrial membrane potential depolarization in TNBC cells by up to 68% (Figure S38). Moreover, after cells were exposed to 20 nM AS-TP, the DCF fluorescence intensity elevated significantly, and the intracellular ROS production improved 3.7 folds as compared to the control cells (Fig. 8H). Cytochrome C (Cyto C) as an apoptotic pathway marker is released from the mitochondria into the cytoplasm to activate downstream apoptotic signals [35]. Briefly, AS-TP stimulated the mitochondrial apoptotic pathway, resulting in promotion of increased expression of Cyto C, enhancement of pro-apoptotic protein Bax, inhibition of anti-apoptotic protein Bcl-2 expression, and reduction of downstream related proteins PARP, Bid and Caspase-3/8/9 expression (Figure S39).

### Pharmacokinetic profile and biological safety of AS-TP

**Fig. 9:**
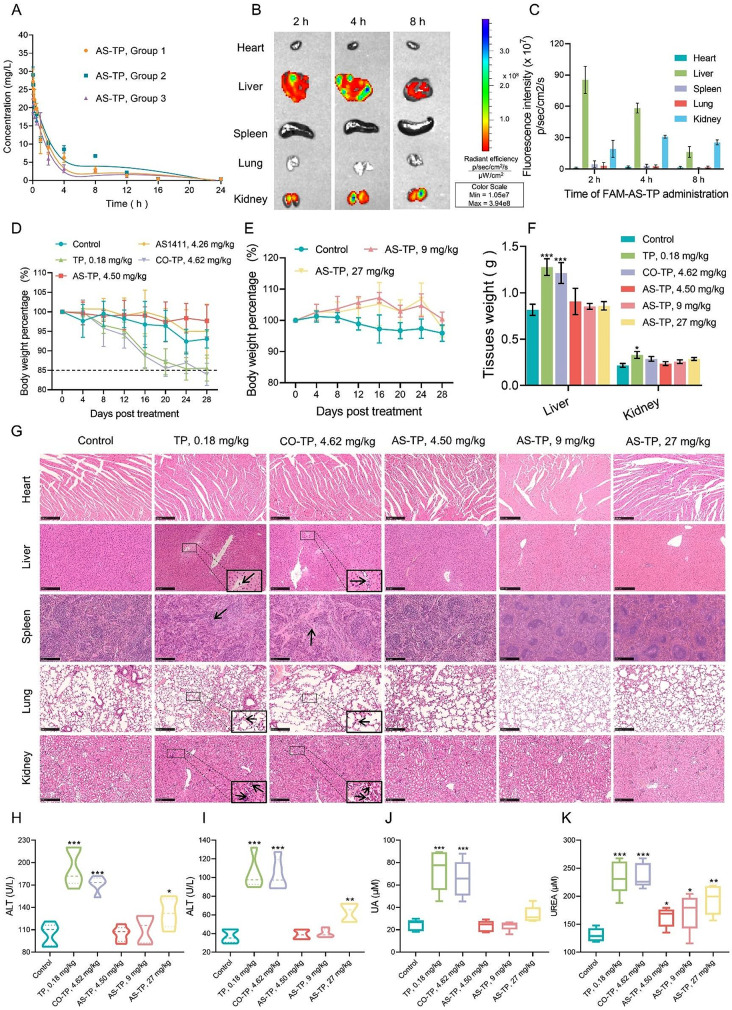
Pharmacokinetic profile and biological safety of AS-TP. (**A**) The pharmacokinetic of AS-TP in serum curve in mice. After receiving an intravenous injection of AS-TP at a concentration of 4.5 mg/kg, mice were given blood samples at 11 different times (3 min, 5 min, 15 min, 30 min, 1 h, 2 h, 4 h, 8 h, 12 h, 16 h, and 24 h) so that the contents of AS-TP could be measured. *n* = 3. (**B**) Using biophotonic imaging, the distribution of FAM-AS-TP in the heart, liver, spleen, lung, and kidney at various intravenous injection timings. (**C**) Semiquantitative examination of the main viscera’s fluorescence intensity at various administration timings. *n* = 3. (**D**) The percentage change in mice’s body weight at the same concentrations of TP, AS411, AS-TP, and CO-TP. *n* = 5. (**E**) The mice’s body weight curves after receiving 9 mg/kg and 27 mg/kg of AS-TP. (**F**) The mice’s heart, liver, spleen, lung, and kidney weights after their treatments were finished. (**G**) Histological evaluations of the tissues in the various treatment groups using H&E staining. Arrows in the lung: alveolar congestion; arrows in the kidney: glomerular atrophy, degradation of glomerular structure; arrows in the liver: inflammatory cell infiltration, hepatocyte structural damage; arrows in the spleen: inflammatory cell infiltration, no germinal center. Scale bars, 100 μm. (**H**-**K**) Determination of AST, ALT, CREA, and UA levels in mouse serum. *n* = 5. Compared to the control group, *p < 0.05, **p < 0.01, ***p < 0.001, ns *p* > 0.05

AS-TP was injected into mice via tail vein at a dose of 2.5 mg/kg to obtain pharmacokinetic parameters. As illustrated in Fig. 9A and Table S2, the elimination half-life (T1/2β) of AS-TP after single-dose administration was approximately 7.32 h, which resulted in a 29-fold improvement in TP (T1/2β = 0.25 h) recorded in previous studies [36]. Additionally, the area under the concentration-time curve (AUC) of AS-TP was evaluated to be 84.87 mg/L*h with a clearance of 0.17 L/h/kg, which suggested that AS-TP exhibited better circulating capacity and bioavailability than TP in vivo. Moreover, distribution imaging tests in vivo revealed that AS-TP was massively distributed in the liver and kidneys, and the FAM fluorescence of AS-TP gradually vanished in the liver and became predominantly concentrated in the kidneys as the duration of dosing increased (Fig. 9B, C). To investigate the potential toxicity of AS-TP, Balb/c mice were treated with AS-TP (4.50 mg/kg), CO–TP (4.62 mg/kg), TP (0.18 mg/kg), and AS1411 (4.26 mg/kg) for 7 times on 4 days/dose. AS-TP and AS1411 exerted an absence of systemic toxicity, in contrast to CO-TP and TP, which presented severe toxicity, with body weight percentages of mice below 85% (Fig. 9D). To further investigate the safety of AS-TP, the administered dose of AS-TP was increased to 9 mg/kg and 27 mg/kg, respectively, demonstrating that AS-TP remained non-decreasing in body weight in mice (Fig. 9E). Subsequently, the mice were dissected to determine their tissue weights, and revealed that CO-TP and TP led to enlargement of the liver and kidneys, whereas AS-TP maintained no noticeable histotoxicity at multidose (Fig. 9F). Afterwards, HE staining showed that both TP and CO-TP induced lesions in the liver, kidneys, spleen and lungs of mice, including necrosis of liver tissue, glomerular atrophy, thickening of alveolar walls and structural reorganization of the spleen, whereas no obvious organ damage occurred in AS-TP at multiple doses (Fig. 9G). Moreover, in vivo biochemical levels confirmed that compared with the control group, CO-TP, TP and AS-TP at high doses contributed to an elevation of serum alanine aminotransferase and glutamic oxalate aminotransferase, while TP and CO-TP also brought about an increase in the levels of uric acid and urea, indicators of renal function (Fig. 9H-K). Overall, TP and CO-TP in general affected the indicators of hepatic and renal function to a larger extent. The foregoing demonstrated that TP and CO-TP possessed potential systemic toxicity and particularly induced hepatic and renal tissue damage, whereas AS-TP could be well tolerated in mice.

### 10 the superiority of AS-TP against TNBC vs. clinical therapeutic medicines

**Fig. 10:**
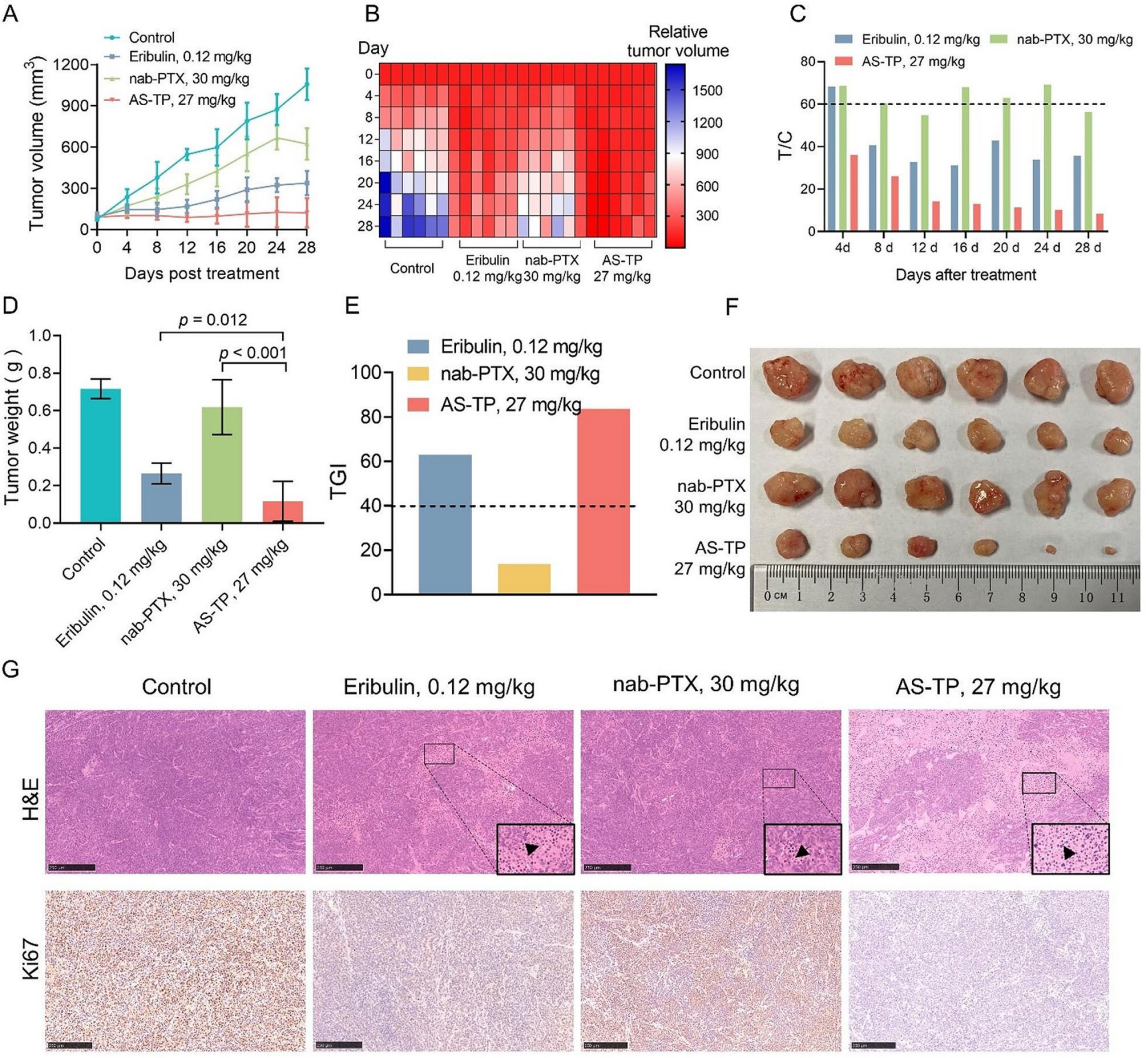
Anticancer efficacy of AS-TP in comparison to therapeutic medicines in vivo. (**A**) Tumor volume analysis of MDA-MB-231 tumor-bearing nude mice after administration of saline, nab-PTX, Eribulin and AS-TP.(**B**-**C**) The relative tumor volume (**B**) and relative tumor growth rate T/C (**C**) of MDA-MB-231 xenografts during treatment. (**D**) MDA-MB-231 xenograft tumor weight at day 32 post-treatment. (**E**) The tumor growth inhibition rate was calculated based on the tumor weight. (**F**) Representative images of tumor tissues from different experimental groups. (**G**) H&E staining and immunohistochemistry analysis of Ki67 in subcutaneous tumors. Scale bars, 100 μm. *n* = 6

To date, chemotherapy is the main option for the treatment of TNBC. Boldly, based on the favorable therapeutic indexes for AS-TP above, we adopted the clinical drug albumin paclitaxel (nab-PTX) and the clinical phase III drug Eribulin as the comparison groups, while set up a high dose group exceeding the pre-dose, for the purpose of examining the safety of AS-TP in TNBC and advancing the preclinical studies. As illustrated in Fig. 10A, B, with no growth tendency of the tumor tissue in the AS-TP treatment group, the tumor volume was about 100 mm [3] with the relative tumor volume consistently under 100%. At the end of treatment in the AS-TP group, where tumor volume could not be measured in 2 mice, the cure rate was 40%, with a T/C% index of 8.4% and TGI index of 83.7% (Fig. 10C-F). In comparison, the tumor volume expanded slowly under Eribulin treatment, and upon completion of the treatment, its tumor volume was approximately 339 mm [3], with a T/C% index of 35.7% and a TGI index of 63.0% (Fig. 10A-F). However, the tumor volume under nab-PTX treatment was about 623 mm [3], with a T/C% index of 56.4% and a TGI index of only 13.7%. Furthermore, pathologic evaluations revealed that nab-PTX and Eribulin could damage the structure of tumor tissue and lessen the expression of Ki67 in TNBC (Fig. 10G). Interestingly, pathological analysis revealed that AS-TP produced significant necrotic tumor cell death and total suppression of cell proliferation, resulting in subsequent tumor ablation (Fig. 10G). And routine blood test results showed that the proportions of leukocytes, lymphocytes, and neutrophils increased in the Eribulin-treated mouse (Table S3). Collectively, all the above data could evidence that AS-TP provided better antitumor efficacy and safety profiles compared to clinical agents employed for the TNBC treatment.

## Discussion

Molecular targeted therapy has advanced significantly with the progress in molecular biotechnology and the deepening understanding of cancer pathogenesis [37]. Despite this, ADC drugs have not yet altered the landscape of targeted therapy for TNBC. The crucial factor for cytotoxin targeted delivery therapy remains the specific expression of receptors in tumor tissue [38, 39]. The NuP encoded by NCL has recently been identified as a candidate receptor for targeted therapy in tumors [40]. This study initially assessed the expression of NCL in breast cancer and found high levels, particularly in TNBC. Given its role as a potential receptor for new targeted therapies, its presence on the cell membrane is crucial. Subsequently, we investigated the subcellular localization of the NuP, which was found to be abnormally expressed on the cell membranes of TNBC cells. The first nucleic acid aptamer used in clinical trials, AS1411, has progressed to phase II trials [41]. Although it targets the overexpressed NuP on tumor cell membranes to exert an anti-tumor effect, the trial was halted due to lack of efficacy. Nevertheless, our research indicates that AS1411 can effectively target and bind to the RGG domain of the NuP with high affinity, offering promise for targeting TNBC.

Considering the specific affinity to NuP overexpressed on the aggressive malignancy of TNBC, we exploited NuP-targeting aptamer AS1411 to connect with the extremely cytotoxic TP with a novel acid-hypersensitive linker to fabricate ApDC, which could achieve selective transport of cytotoxic TP to target cells and release the intact TP efficiently under the acidic tumor microenvironment. Based on the structure-activity relationship of TP, the innovatively designed acetal ester linker, compared with the traditional acid-labile vinyl ester linker, ensured the remarkable stability of the conjugate in the blood circulation system and the dexterous sensitivity to the acidic environment of tumor tissue, implying that the cytotoxicity of TP was confined in normal cells, whereas the antitumor activity was recovered in tumor cells.

Binding of AS1411 to TP does not alter its targeting to NuP. Conversely, the cellular uptake of AS-TP was observed in an administration concentration as well as NuP-expression dependent manner in tumor cells, whereas the uptake of CO-TP by tumor cell lines expressing various levels of NuP on the cell membrane tended to no obvious distinction. Regardless of absolute cytotoxicity in proliferation inhibition experiments, similar trends were observed in different cell lines. In comparison to CO-TP, AS-TP demonstrated a stronger ability to kill triple-negative breast cancer cells with NuP overexpression, as observed across various TNBC cell lines. However, it also exhibited quite cytotoxicity to cells with NuP low expression compared to CO-TP, consistent with the cellular uptake of both conjugates. In addition, AS1411 inhibits the uptake of AS-TP by TNBC cells and reverses the killing effect of AS-TP on TNBC cells, which could account for the multiple endocytic pathways for both types of cells for small molecule drugs entering intracellularly in large quantities. Yet TNBC cells primarily engaged in binding to AS1411 through NuP on the membrane to promote the micropinocytosis of conjugated TP, while other cells could not perform this manner. A remarkably impressive achievement was that AS-TP perfectly exploited the oncology targeting of AS1411, in contrast to CO-TP, whereby the features predicated on AS1411 could facilitate the apparent enrichment of its modified conjugate in tumor tissues instead of indistinguishable distribution in various viscera. The above data indicated that the interaction between NuP and AS1411 performed a critical role in the access of the conjugated TP to tumor cells.

In line with in vitro research, AS-TP demonstrated an improved pharmacologic profile characterized by stronger tumor cell accumulation and superior intra-tumoral pharmacodynamic, resulting in higher antitumor potency. Strong anticancer efficacy was demonstrated by AS-TP in typical NuP-over expressing cancers, such as TNBC, which has been shown to be difficult for ADCs to treat. TP and CO-TP, nevertheless, have not demonstrated effectiveness in xenografts with overexpressed NuP. A new microtubule dynamics inhibitor, Eribulin mesylate, has a remarkable anti-tumor impact in patients with TNBC [42]. Chemotherapeutic agents like paclitaxel are still the gold standard for TNBC, which is highly deadly [43]. AS-TP displayed excellent anticancer efficacy in xenograft animals containing typical TNBC cells, as evidenced by TGI and cure rate. On the other hand, nab-PTX did not work in the identical models. An initial investigation into maximum tolerated dosage treatment revealed that Eribulin had a less effective therapeutic impact than AS-TP, which translated into an increase in AS-TP’s antitumor effectiveness over Eribulin. A broad strategy for improving treatment results in TNBC may be receptor-mediated AS-TP targeted therapy based on NuP-mediated.

Poor water solubility and low bioavailability of TP restrict its clinical application. Although structurally modified TP derivatives, for example PG490, Minnelide and LLDT-8, are currently in various stages of clinical trials, they remain an ineffective and inadequate breakthrough of the above-mentioned bottlenecks [44]. In the toxicity research, AS-TP displayed less organ harm than TP, despite that both had identical anti-tumor mechanisms. As a result of the extended period following ingestion, AS-TP gradually moved from the liver to the kidney for processing. The decreased toxicity seen in the metabolic organ in the toxicity investigation is probably due to the better stability and lower exposure levels of AS-TP itself. At the same time, the half-life of the AS1411-modified compound was significantly longer in normal mice when compared to TP, which helped to increase TP’s bioavailability and reduce the mice’s toxicity from TP.

AS1411, as a G- rich nucleic acid in phase II clinical trial, is now abundantly applied as a targeting vehicle or ligand for the functional delivery of drugs in the field of nanomedicine[9b]. However, these AS1411-modified nanodrugs generally suffered from inhomogeneity of particles, lipid/polymer material instability, immunogenicity, and potential toxicity [45]. On the contrary, we designed and prepared AS-TP as a single macromolecule, quantitatively combining TP with AS1411 through a novel acid-sensitive linker, without any excipients or other components, to demonstrate the respective excellent properties of TP and AS1411 in vivo. As a promising class of nucleic acid drugs and an alternative to ADCs, this work established an innovative technological platform for the development of ApDCs, exploited new applications for non-druggable toxic small molecules, and realized the evolution of precision medicine for tumors from concept to reality.

### Electronic supplementary material

Below is the link to the electronic supplementary material.

Supplementary Material 1

Supplementary Material 2

## Data Availability

Data will be made available on request.

## Abbreviations

TNBC: Triple-negative breast
ER: Estrogen receptor
PR: Progesterone
HER2: Human epidermal growth factor
NAA: Nucleic acid aptamer
NuP: Nucleolin protein
CA: Carbonic anhydrase, ADC, antibody drug conjugate
AML: Acute myeloid leukemia
B-ALL: B-cell acute lymphoblastic leukemia
ApDC: Aptamer drug conjugate
TP: Triptolide
AS-TP: AS1411-triptolide conjugate
RGG: Glycine/arginine-rich
SPR: Surface plasmon resonance
SA: Succinic anhydride
CO: Cytosine-rich oligonucleotide
FAM: Fluorescein amidate
Cyto C: Cytochrome C
