# Supplementary material for: Aptamer-functionalized triptolide with release controllability as a promising targeted therapy against triple-negative breast cancer
Source: J Exp Clin Cancer Res. 2024 Jul 25;43:207. doi: 10.1186/s13046-024-03133-5 (PMC11270970; doi:10.1186/s13046-024-03133-5)
Supplement: Supplementary file 1 — Supplementary Material 1 [file 13046_2024_3133_MOESM1_ESM.docx]

**Supporting Information**

**Aptamer-functionalized triptolide with release controllability as** **a promising** **targeted therapy against triple-negative breast cancer**

Yao Chen^a,1^, Jirui Yang^a,1^, Chuanqi Wang^a,1^, Tianbao Wang^b,1^, Yingjie Zeng ^a,1^, Xiao Li^a^, Yi Zuo^a^, Hongyu Chen^a^, Chaozheng Zhang^a^, Yuening Cao^a^, Chen Sun^a^, Maolin Wang^c^, Xiujun Cao^a^, Xian Ge^a^, Yilan Liu^d^, Ge Zhang^e^, Yun Deng^a,*^, Cheng Peng^a,*^, Aiping Lu^e,*^ and Jun Lu^a,e,*^

1. State Key Laboratory of Southwestern Chinese Medicine Resources, School of Pharmacy, Chengdu University of Traditional Chinese Medicine, Chengdu, 611137, China;
2. Sichuan Clinical Research Center for Cancer, Sichuan Cancer Hospital & Institute, Sichuan Cancer Center, Affiliated Cancer Hospital of University of Electronic Science and Technology of China, Chengdu, 610041, China;
3. Clinical Research Center, The First Affiliated Hospital of Shantou University Medical College, Shantou, Guangdong Province, 515000, China;
4. Hematology Department, The General Hospital of the Western Theater Command PLA, Chengdu, 611137, China;
5. Institute for Advancing Translational Medicine in Bone & Joint Diseases, School of Chinese Medicine, Hong Kong Baptist University, 999077, Hong Kong SAR.

^1^ These authors contributed equally to this work.

* Corresponding addressed to: Jun Lu, ljaaa111@163.com; Aiping Lu, aipinglu@hkbu.edu.hk; Cheng Peng, pengchengchengdu@126.com; Yun Deng, dengyun2000@hotmail.com.


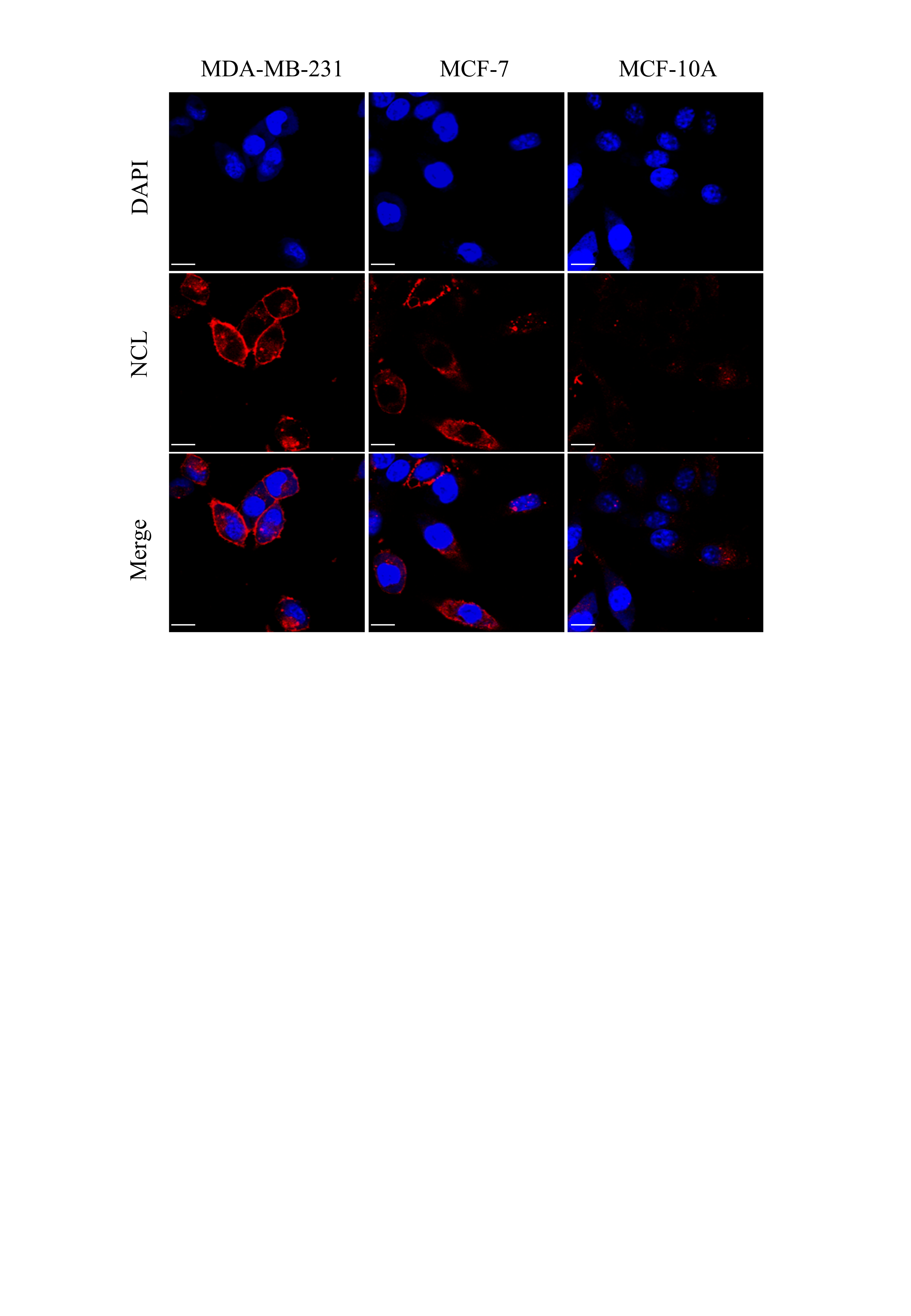


**Figure S**1. NuP expression on cell membranes of different cell lines. The laser confocal microscopy detection of NuP expression on the cell membrane surface of MDA-MB-231, MCF-7, and MCF-10A cells (NuP: red; nucleus: blue. Bar = 10 μm. Magnification: 400X).


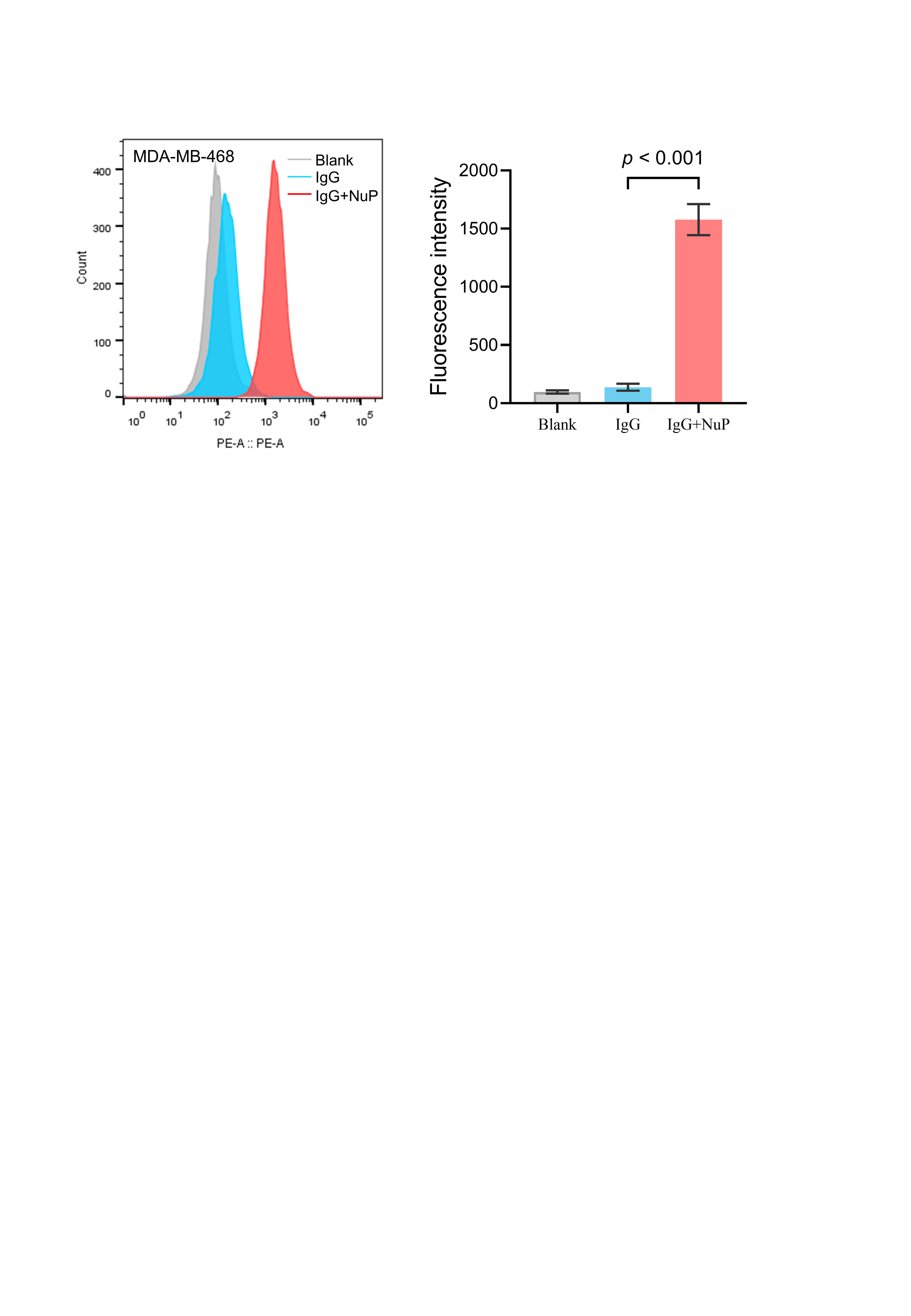


**Figure S2**. The expression of NuP on the surface of triple negative breast cancer cell line MDA-MB-468 cell membrane was measured by flow cytometry.


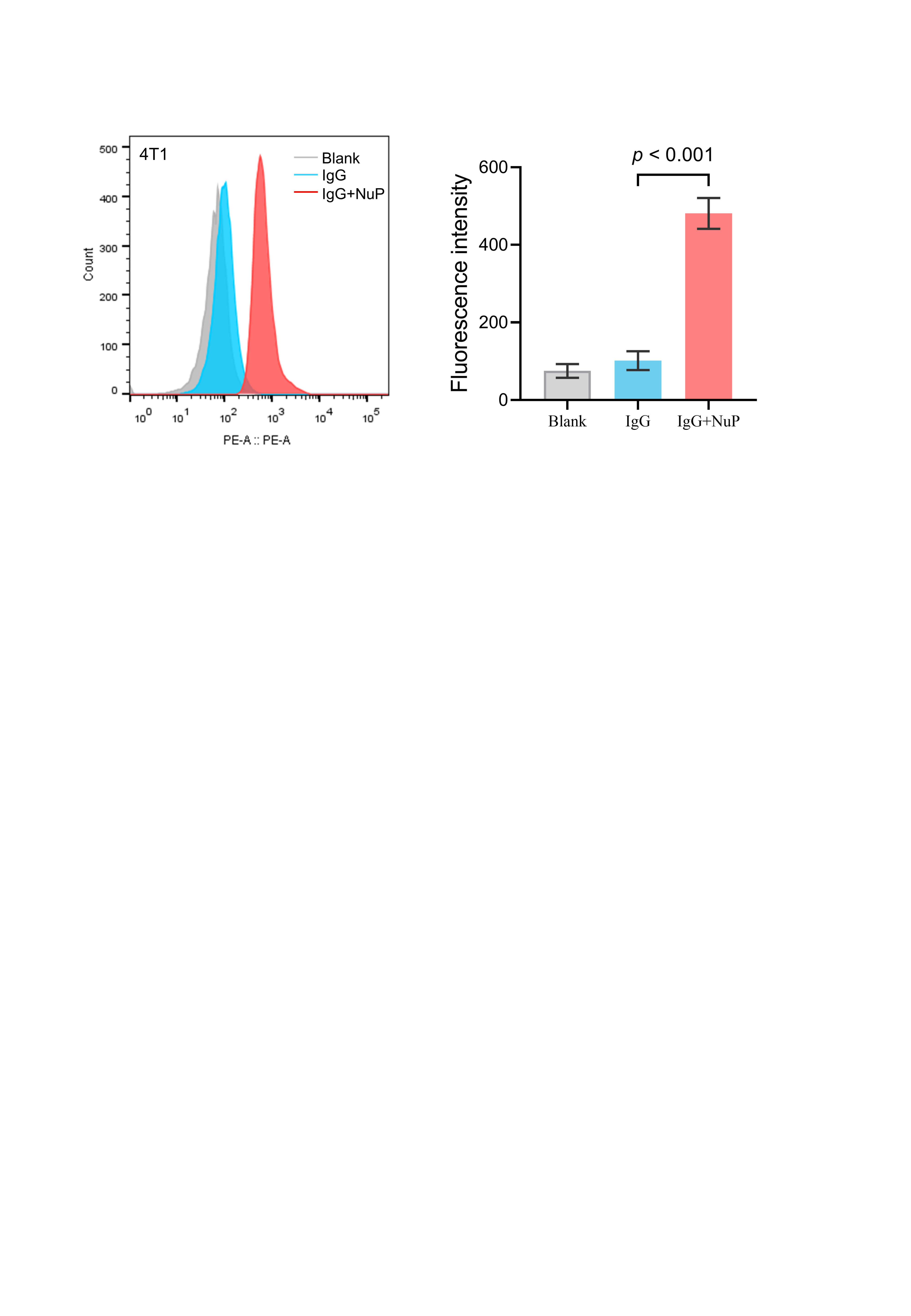


**Figure S3.** The expression of NuP on the surface of triple negative breast cancer cell line 4T1 cell membrane was measured by flow cytometry.

A


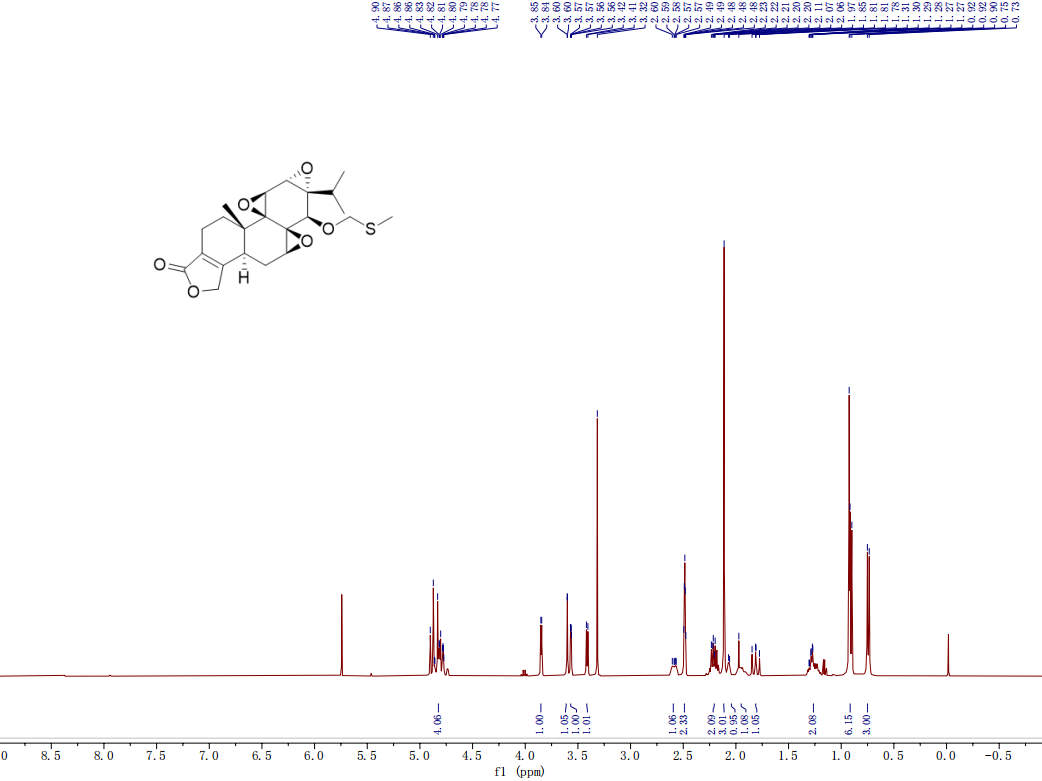


B


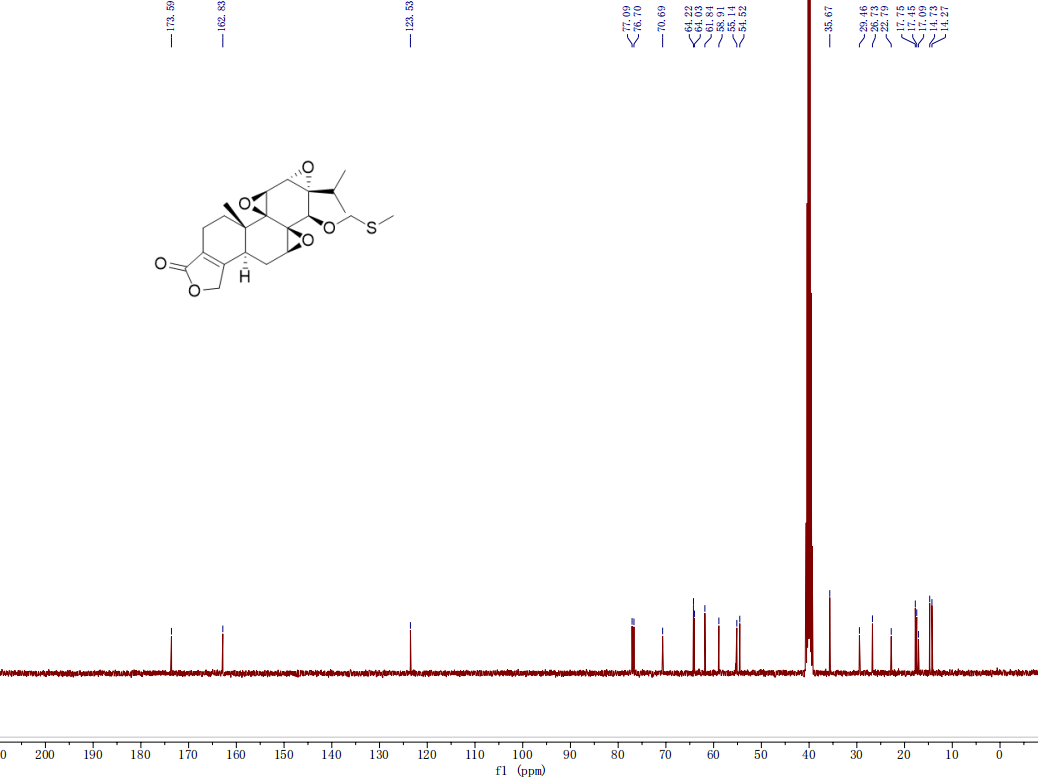


**Figure S4.** ^1^H and ^13^C-NMR spectra of compound 1. Yellow solid, 87.4% yield. A) ^1^H NMR (400 MHz, *d*_6_-DMSO) δ 4.95 – 4.69 (m, 4H), 3.85 (d, *J* = 3.2 Hz, 1H), 3.60 (d, *J* = 0.9 Hz, 1H), 3.57 (dd, *J* = 3.2, 0.9 Hz, 1H), 3.41 (d, *J* = 5.6 Hz, 1H), 2.63 – 2.55 (m, 1H), 2.29 – 2.12 (m, 2H), 2.11 (s, 3H), 2.06 (d, *J* = 4.1 Hz, 1H), 1.97 (s, 1H), 1.81 (dd, *J* = 15.0, 13.3 Hz, 1H), 1.27 (dt, *J* = 6.9, 3.5 Hz, H), 0.95 – 0.88 (m, 6H), 0.74 (d, *J* = 6.9 Hz, 3H). B) ^13^C NMR (100 MHz, *d*_6_-DMSO) δ 173.59, 162.83, 123.53, 77.09, 76.70, 70.69, 64.22, 64.03, 61.84, 58.91, 55.14, 54.52, 35.67, 29.46, 26.73, 22.79, 17.75, 17.45, 17.09, 14.73, 14.27.

A


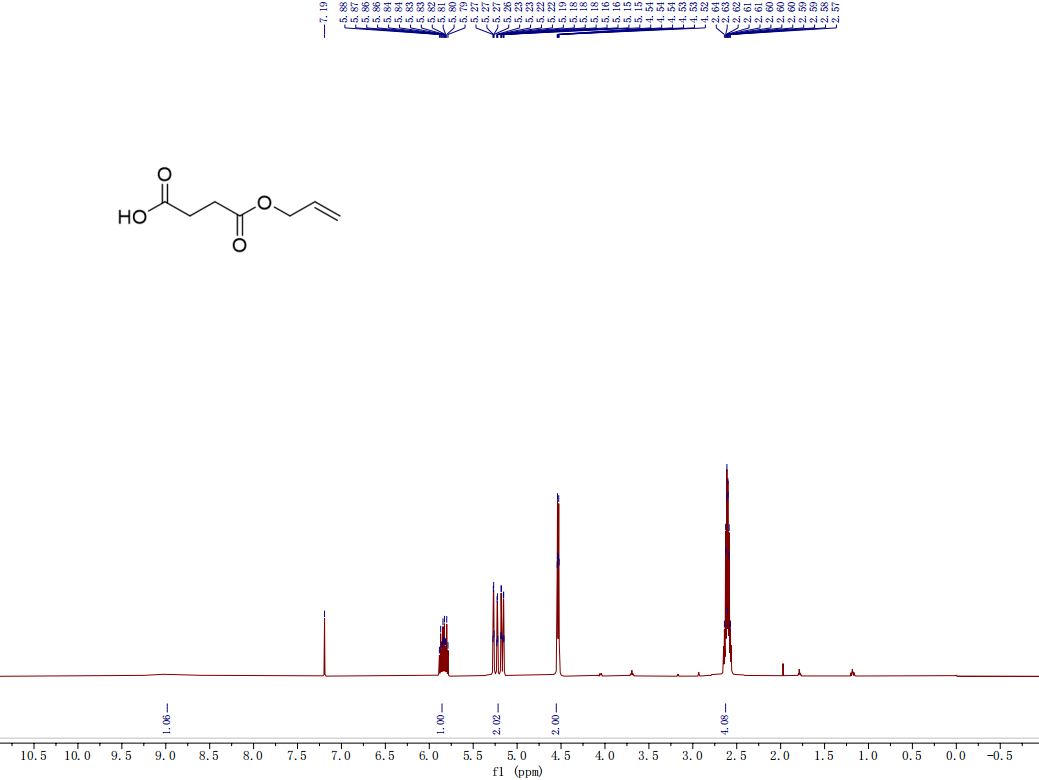


B


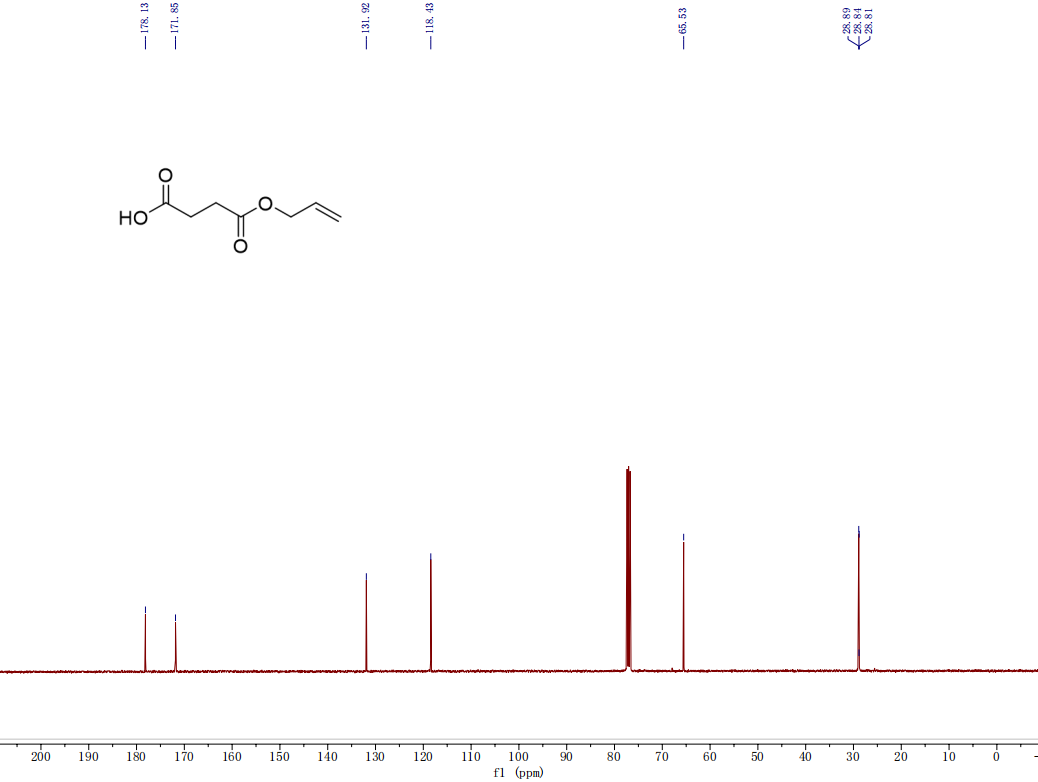


**Figure S5.** ^1^H and ^13^C-NMR spectra of compound 2. White solid, 88.6% yield. A) ^1^H NMR (400 MHz, CDCl_3_) δ 8.90 (s, 1H), 5.90 – 5.78 (m, 1H), 5.30 – 5.13 (m, 2H), 4.53 (d, *J* = 5.7 Hz, 2H), 2.61 (td, *J* = 5.9, 4.8 Hz, 4H). B) ^13^C NMR (100 MHz, CDCl_3_) δ 178.13, 171.85, 131.92, 118.43, 65.53, 28.89, 28.81.

A


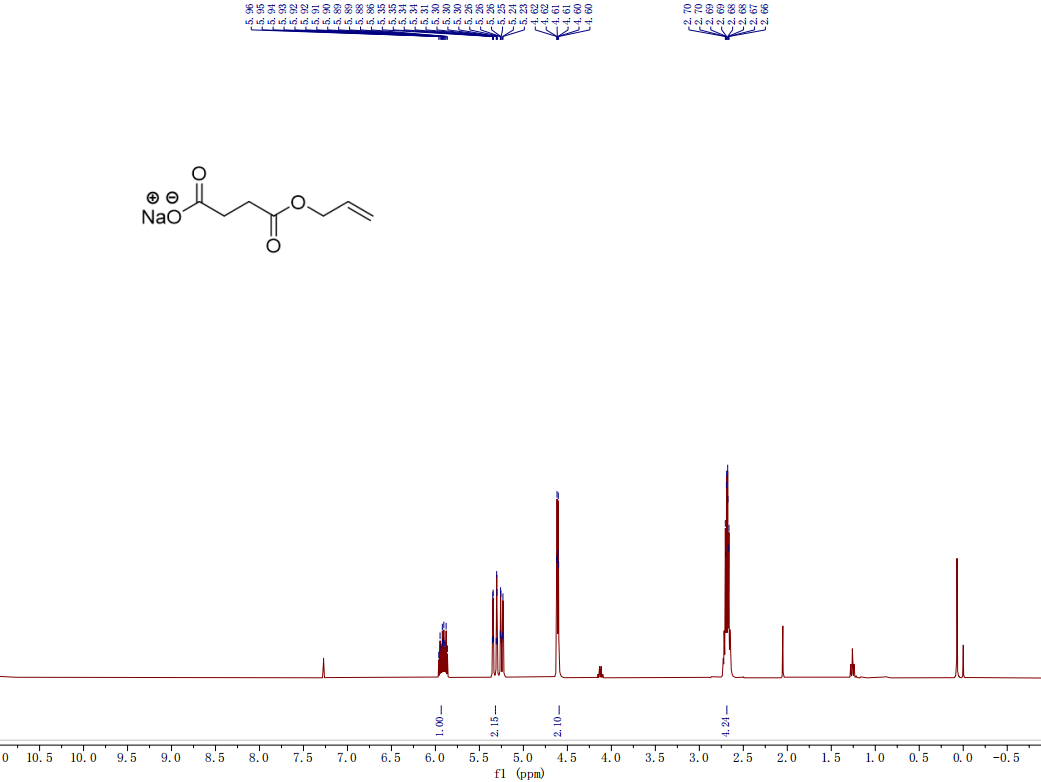


B


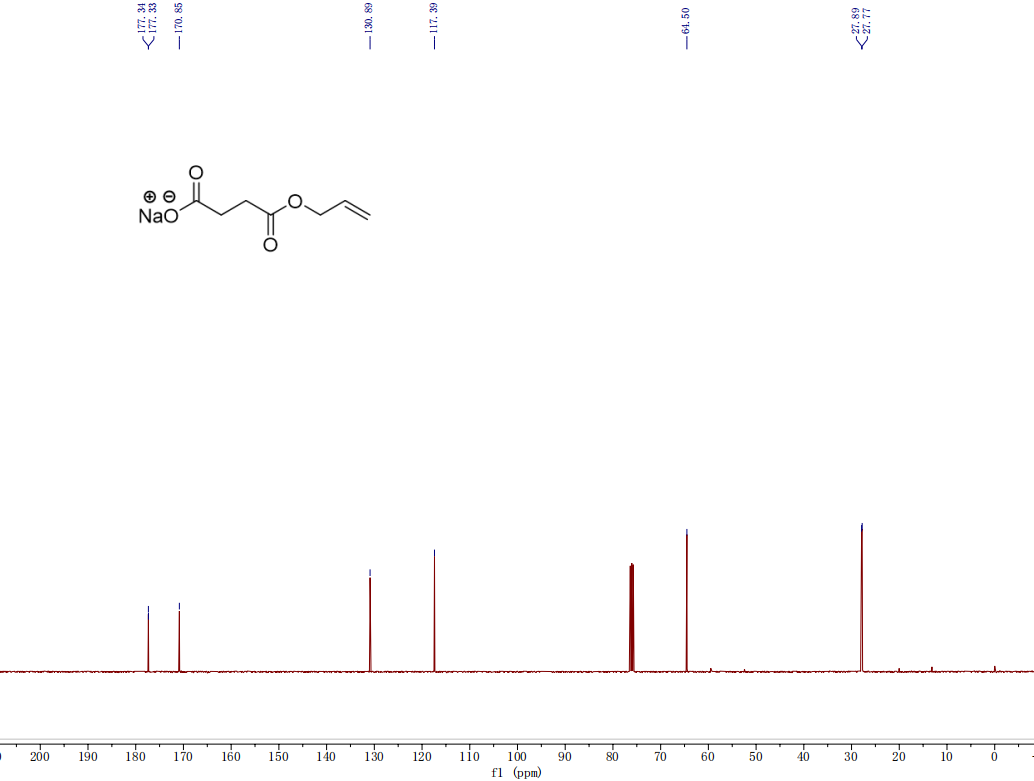


**Figure S6.** ^1^H and ^13^C-NMR spectra of compound 3. White solid, 92.1% yield. A) ^1^H NMR (400 MHz, CDCl_3_) δ 5.98 – 5.84 (m, 1H), 5.33 – 5.22 (m, 2H), 4.61 (dt, *J* = 5.7, 1.4 Hz, 2H), 2.75 – 2.60 (m, 4H). B) ^13^C NMR (100 MHz, CDCl_3_) δ 177.34 (d, *J* = 1.2 Hz), 170.85, 130.89, 117.39, 64.50, 27.77.

A


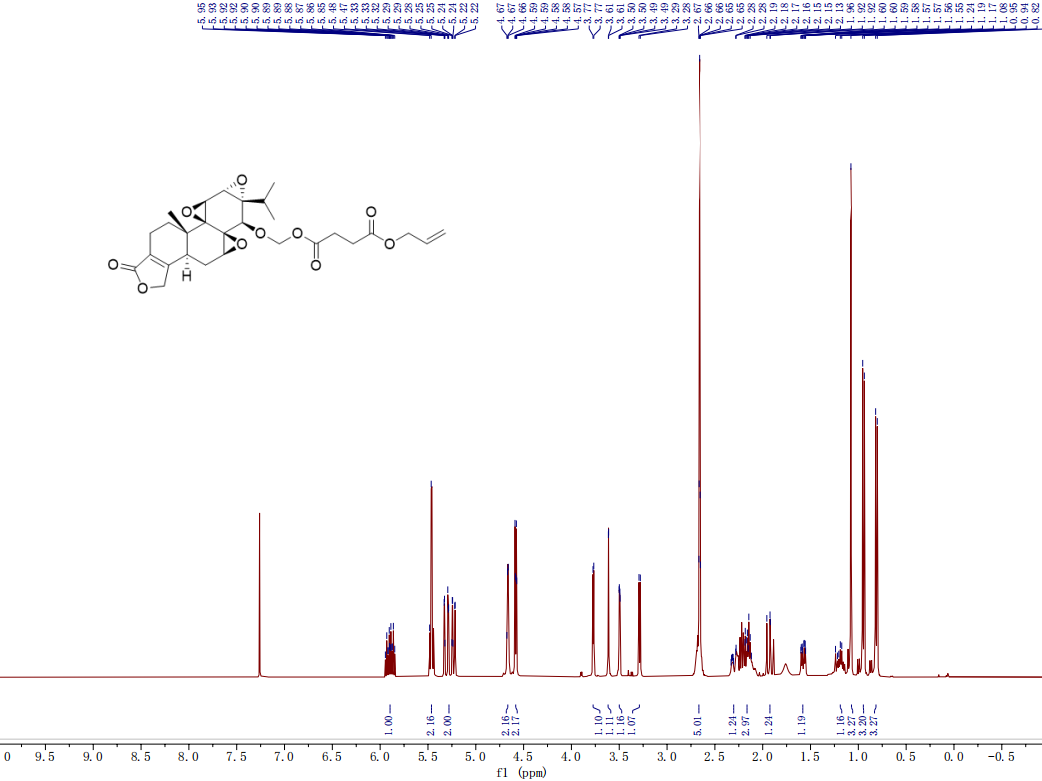


B


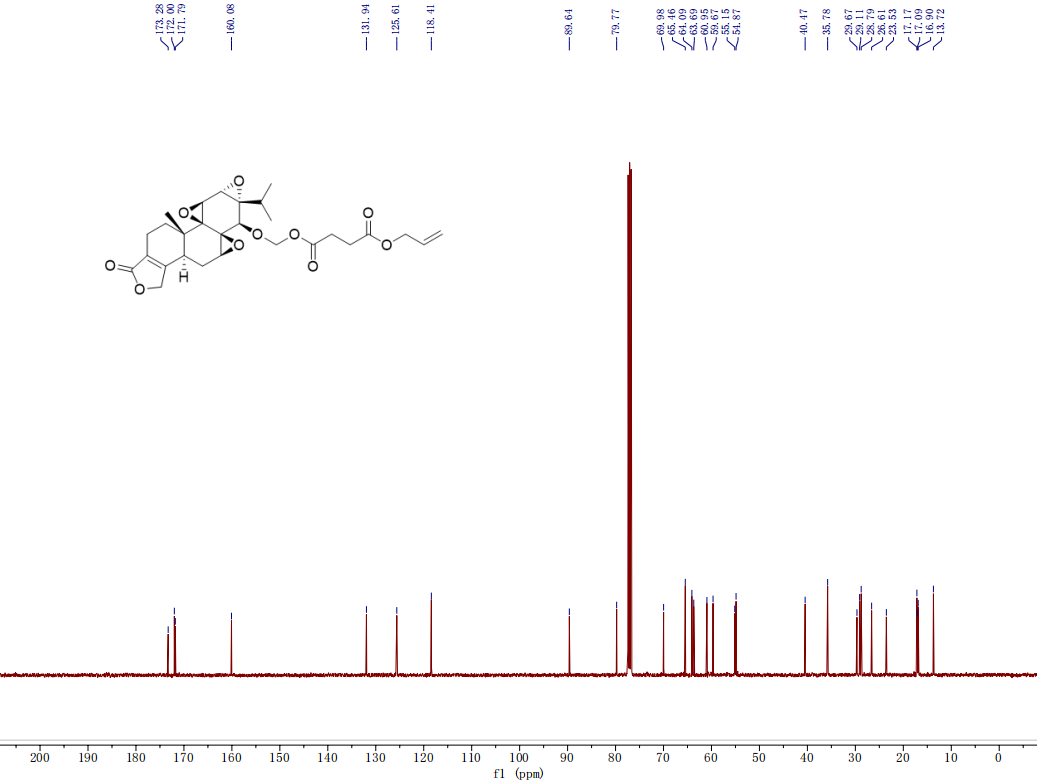


**Figure S7.** ^1^H and ^13^C-NMR spectra of compound 4. Yellow solid, 77.4% yield. A) ^1^H NMR (400 MHz, CDCl_3_) δ 5.90 (ddt, *J* = 17.2, 10.4, 5.7 Hz, 1H), 5.51 – 5.42 (m, 2H), 5.36 – 5.17 (m, 2H), 4.66 (d, *J* = 2.6 Hz, 2H), 4.58 (dt, *J* = 5.7, 1.4 Hz, 2H), 3.77 (d, *J* = 3.2 Hz, 1H), 3.61 (d, *J* = 0.9 Hz, 1H), 3.50 (dd, *J* = 3.1, 0.9 Hz, 1H), 3.29 (d, *J* = 5.6 Hz, 1H), 2.73 – 2.60 (m, 5H), 2.32 (dd, *J* = 4.2, 2.1 Hz, 1H), 2.25 – 2.07 (m, 3H), 1.98 – 1.86 (m, 1H), 1.58 (ddd, *J* = 12.5, 5.5, 1.6 Hz, 1H), 1.28 – 1.09 (m, 1H), 1.08 (s, 3H), 0.95 (d, *J* = 6.9 Hz, 3H), 0.81 (d, *J* = 6.9 Hz, 3H). B) ^13^C NMR (100 MHz, CDCl_3_) δ 173.28, 172.00, 171.79, 160.08, 131.94, 125.61, 118.41, 89.64, 79.77, 69.98, 65.46, 64.09, 63.69, 60.95, 59.67, 55.15, 54.87, 40.47, 35.78, 29.67, 29.11, 28.79, 26.61, 23.66, 17.17, 17.09, 16.90, 13.72.

A


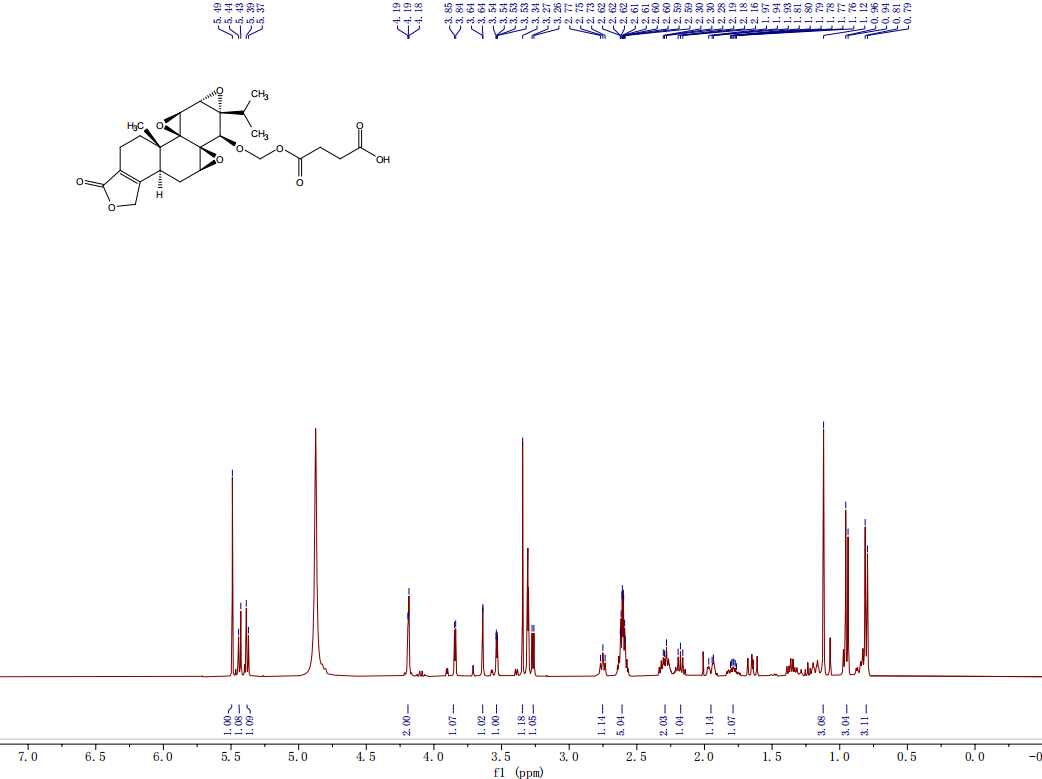


B


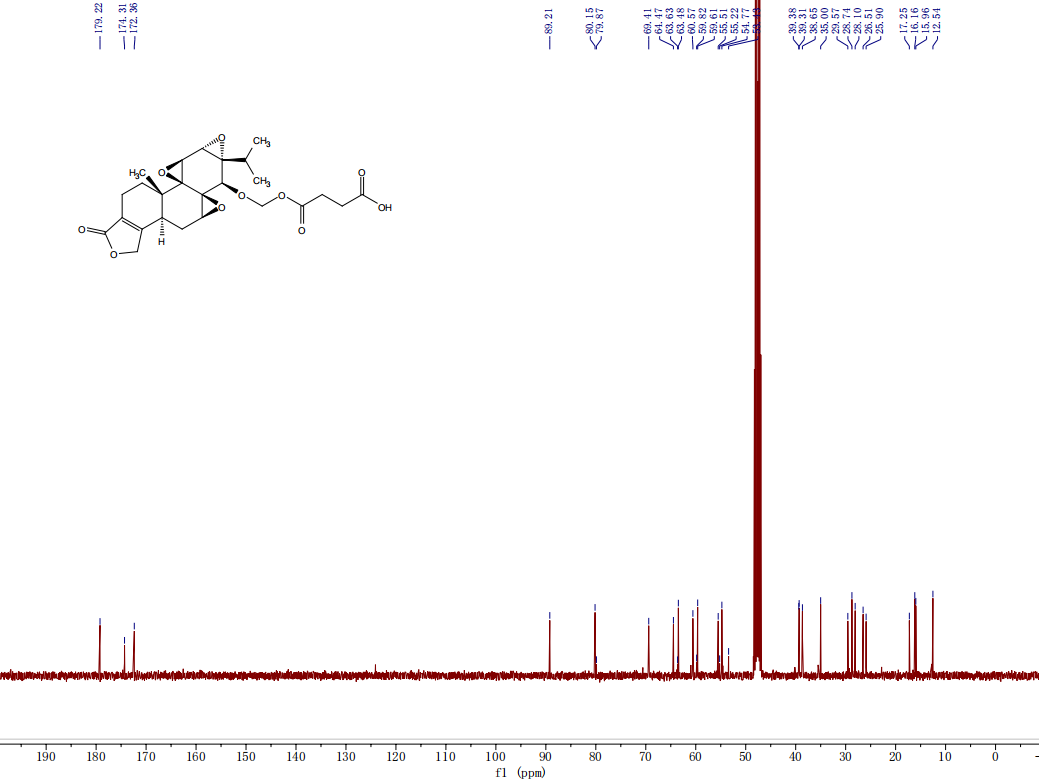


**Figure S8.** ^1^H and ^13^C-NMR spectra of compound 5. Yellow solid, 54.5% yield. A) ^1^H NMR (400 MHz, *d*_4_-MeOD) δ 5.49 (s, 1H), 5.44 (d, *J* = 6.5 Hz, 1H), 5.38 (d, *J* = 6.4 Hz, 1H), 4.24 – 4.14 (m, 2H), 3.84 (d, *J* = 3.2 Hz, 1H), 3.64 (d, *J* = 0.9 Hz, 1H), 3.54 (dd, *J* = 3.3, 1.0 Hz, 1H), 3.34 (s, 1H), 3.27 (d, *J* = 5.5 Hz, 1H), 2.75 (t, *J* = 6.8 Hz, 1H), 2.60 (tq, *J* = 4.6, 2.1 Hz, 5H), 2.36 – 2.22 (m, 2H), 2.25 – 2.10 (m, 1H), 1.95 (d, *J* = 13.7 Hz, 1H), 1.79 (dq, *J* = 7.8, 3.5 Hz, 1H), 1.12 (s, 3H), 0.95 (d, *J* = 6.9 Hz, 3H), 0.80 (d, *J* = 6.9 Hz, 3H). B) ^13^C NMR (100 MHz, *d*_4_-MeOD) δ 179.22, 174.31, 172.36, 89.21, 80.15, 79.87, 69.41, 64.47, 63.63, 63.48, 60.57, 59.82, 59.61, 55.51, 55.22, 54.77, 53.43, 39.38, 39.31, 38.65, 35.00, 29.57, 28.74, 28.10, 26.51, 25.90, 17.25, 16.16, 15.96, 12.54.

A


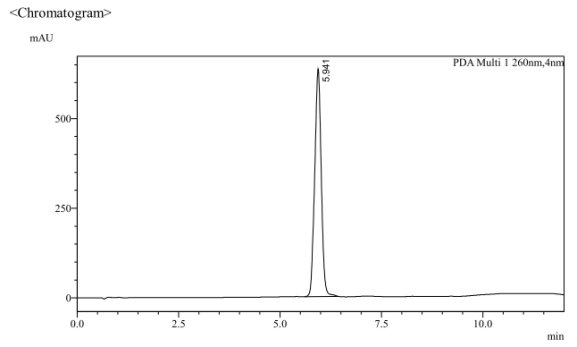


B


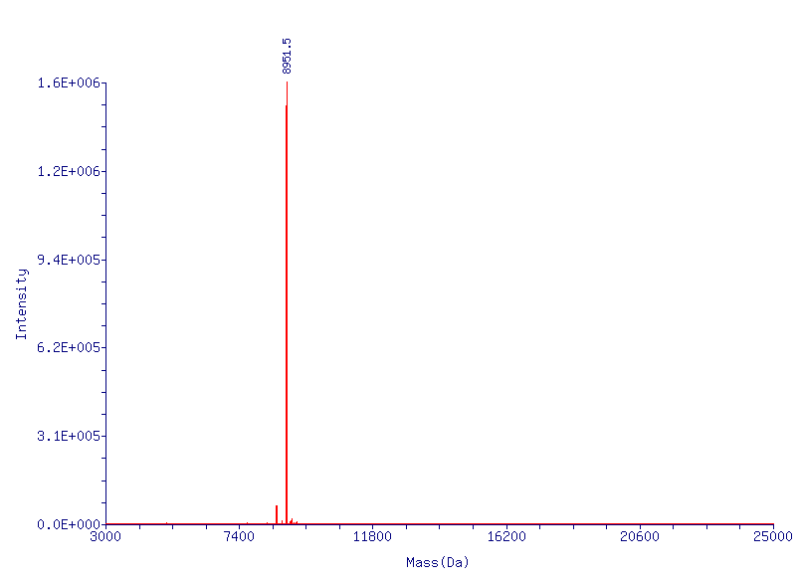


**Figure S9.** HPLC chromatogram and MS spectrum of 6a. A) The HPLC chromatogram of the AS-TP conjugate. B) MS (ESI): [M+H]+: calculated 8951.6, found 8951.5.

A


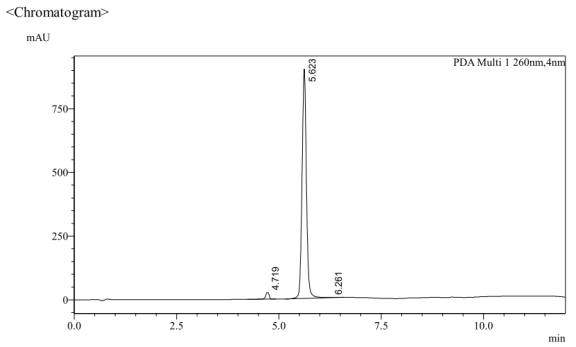


B


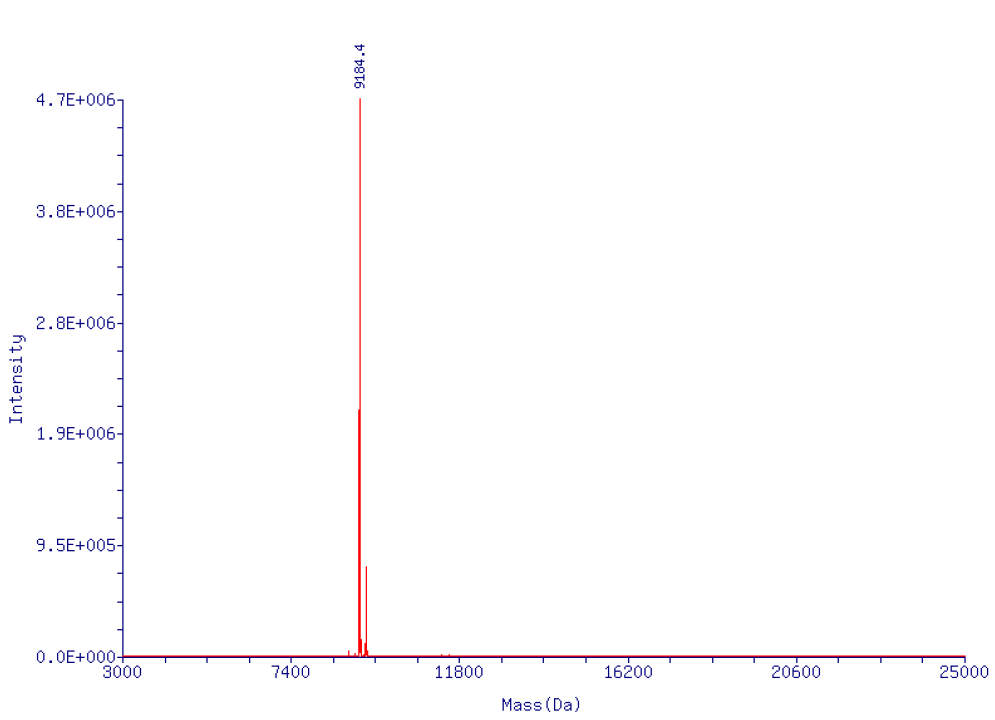


**Figure S10.** HPLC chromatogram and MS spectrum of 6b. A) The HPLC chromatogram of the CO-TP conjugate. B) MS (ESI): [M+H]+: calculated 9184.5, found 9184.4.

A


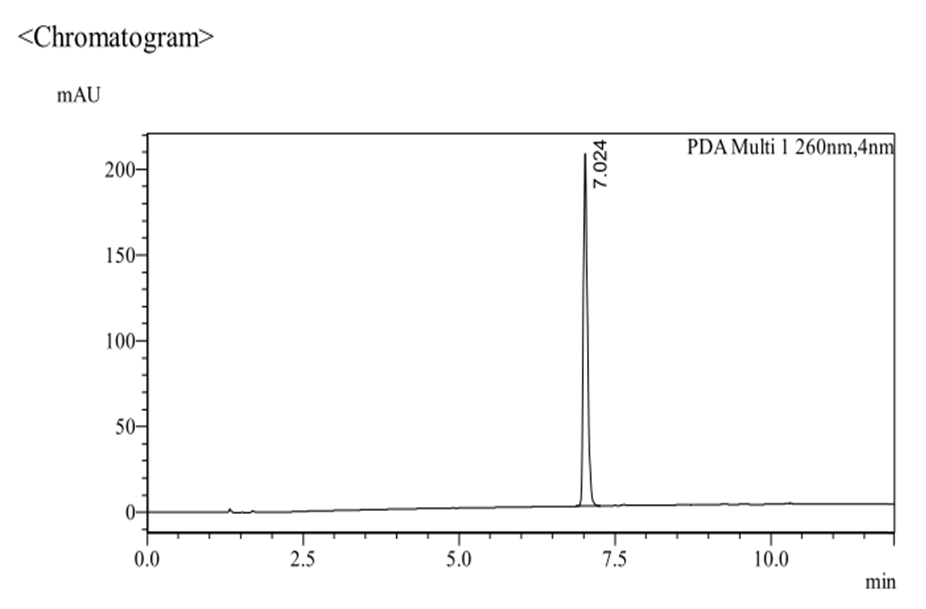


B


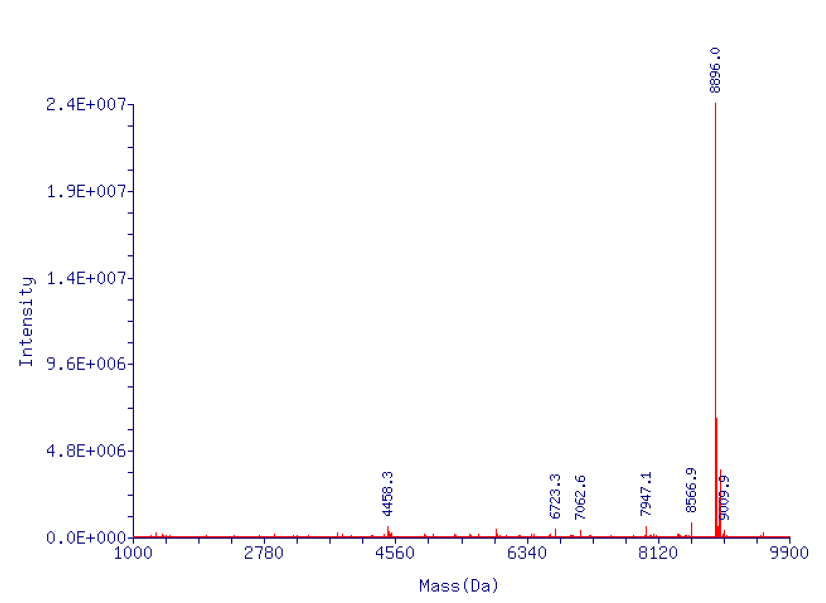


**Figure S11.** HPLC chromatogram and MS spectrum of 6c. A) The HPLC chromatogram of the FAM-AS-TP conjugate. B) MS (ESI): [M+H]+: calculated 8896.2, found 8896.0.

A


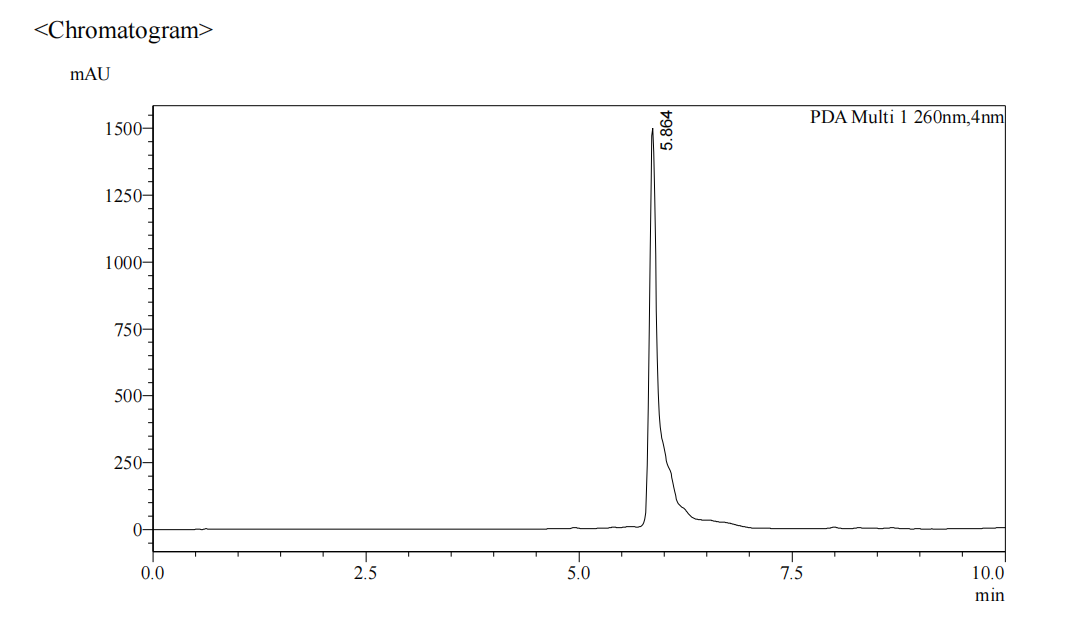


B


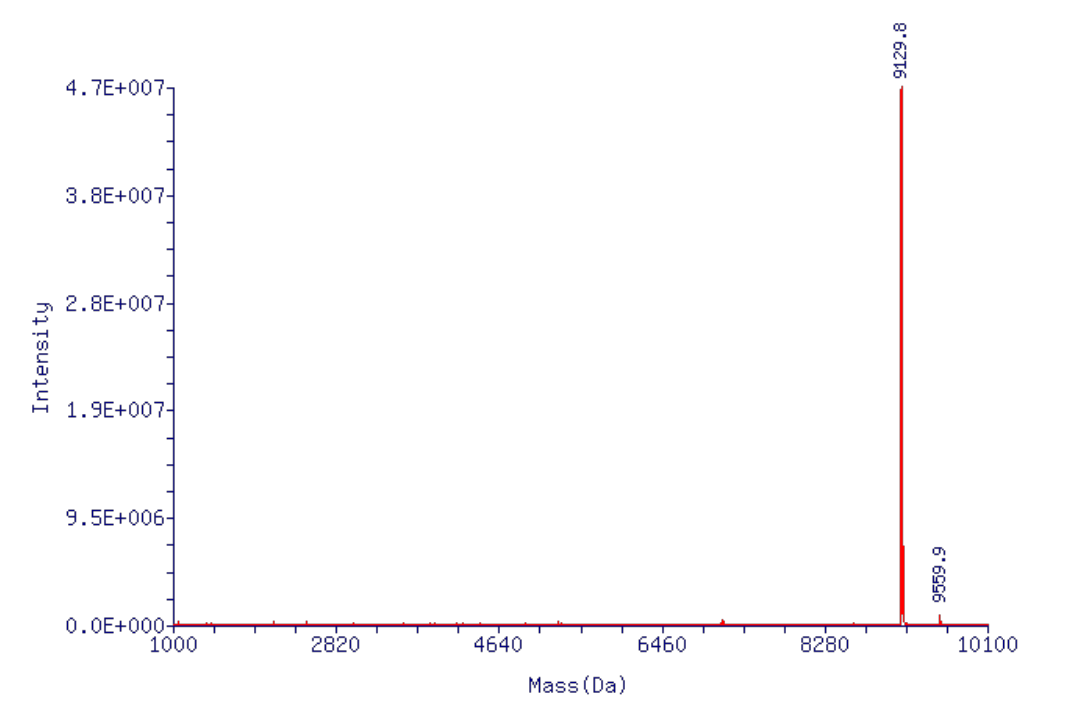


**Figure S12.** HPLC chromatogram and MS spectrum of 6d. A) The HPLC chromatogram of the FAM-CO-TP conjugate. B) MS (ESI): [M+H]+: calculated 9129.1, found 9129.8.

**Figure S13.** Synthesis of dipeptide bond-triptolide conjugate for validating the superiority of the innovative acid-sensitive linker. Note for reagents and conditions: A) [*N*-hydroxy succinimide](https://www.chembk.com/en/chem/N-Hydroxy%20succinimide), EDCI, CH_2_Cl_2_; B) L-citrulline, NaHCO_3_, THF, 50 ℃; C) 4-minobenzyl alcohol, EEDQ, CH_2_Cl_2_; (d) piperidine, DMF; (e) triptolide derivatives, HATU, DIPEA, DMSO. DIPEA = *N*, *N′*-diisopropylethylamine, HATU = 2-(7-Azabenzotriazol-1-yl)-*N*, *N, N*', *N'*-tetramethyluronium hexafluorophosphate, EEDQ = *N*-ethoxycarbonyl-2-ethoxy-1,2-dihydroquinoline.

A


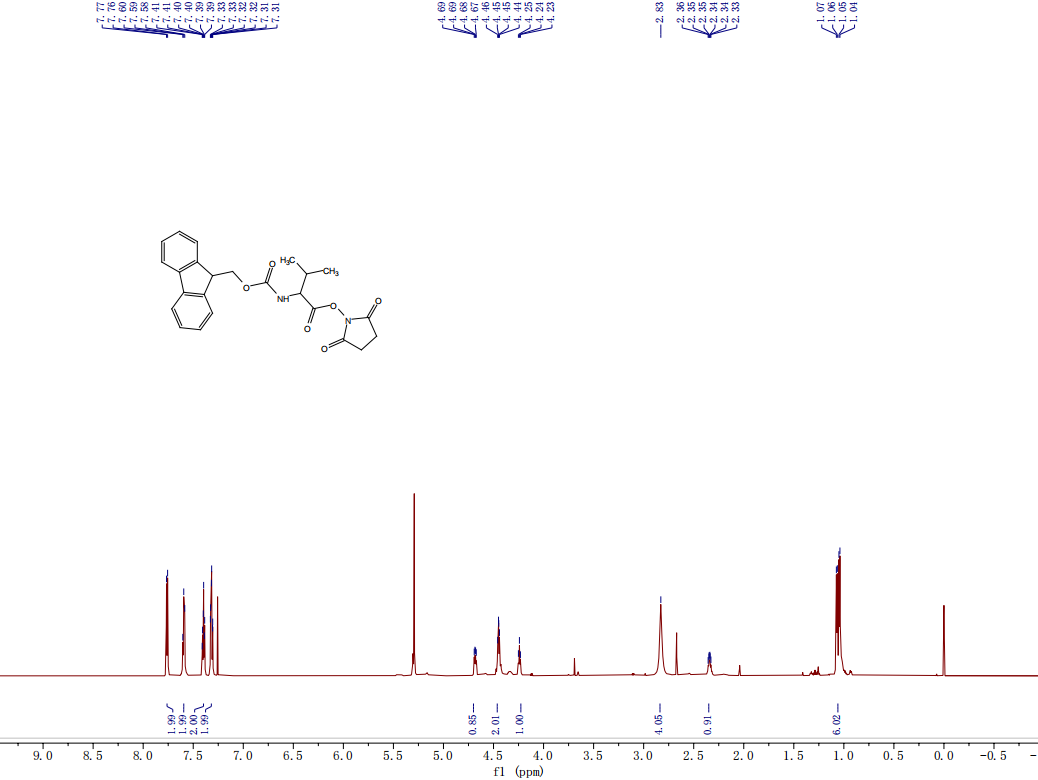


B


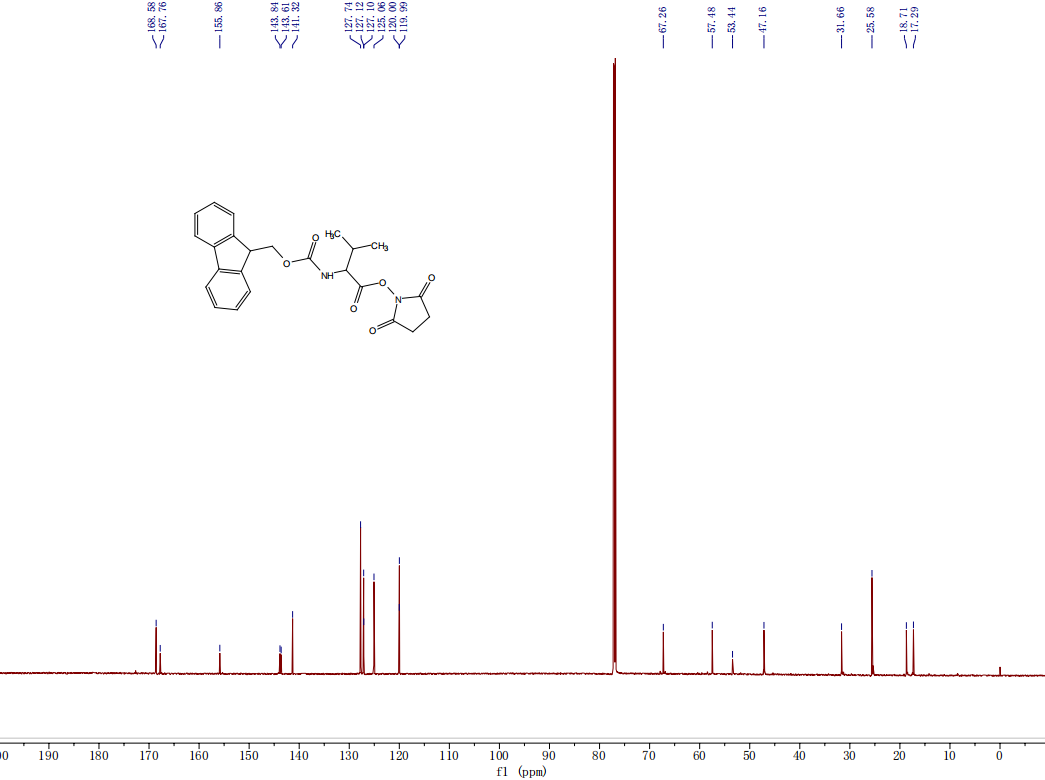


**Figure S14.** ^1^H and ^13^C-NMR spectra of compound 11. White solid, 94.5% yield. A) ^1^H NMR (700 MHz, CDCl_3_) δ 7.76 (d, *J* = 7.6 Hz, 2H), 7.59 (t, *J* = 6.7 Hz, 2H), 7.40 (td, *J* = 7.4, 2.3 Hz, 2H), 7.32 (td, *J* = 7.4, 1.2 Hz, 2H), 4.68 (dd, *J* = 9.3, 4.9 Hz, 1H), 4.44 (td, *J* = 10.8, 9.2, 6.3 Hz, 2H), 4.24 (t, *J* = 7.0 Hz, 1H), 2.83 (s, 4H), 2.34 (ddd, *J* = 13.8, 9.6, 6.0 Hz, 1H), 1.06 (dd, *J* = 18.0, 6.9 Hz, 6H). B) ^13^C NMR (175 MHz, CDCl_3_) δ 168.58, 167.76, 155.86, 143.84, 143.60, 141.32, 127.74, 127.11, 125.06, 120.00, 67.26, 57.48, 53.44, 47.16, 31.66, 25.58, 18.71, 17.29.

A


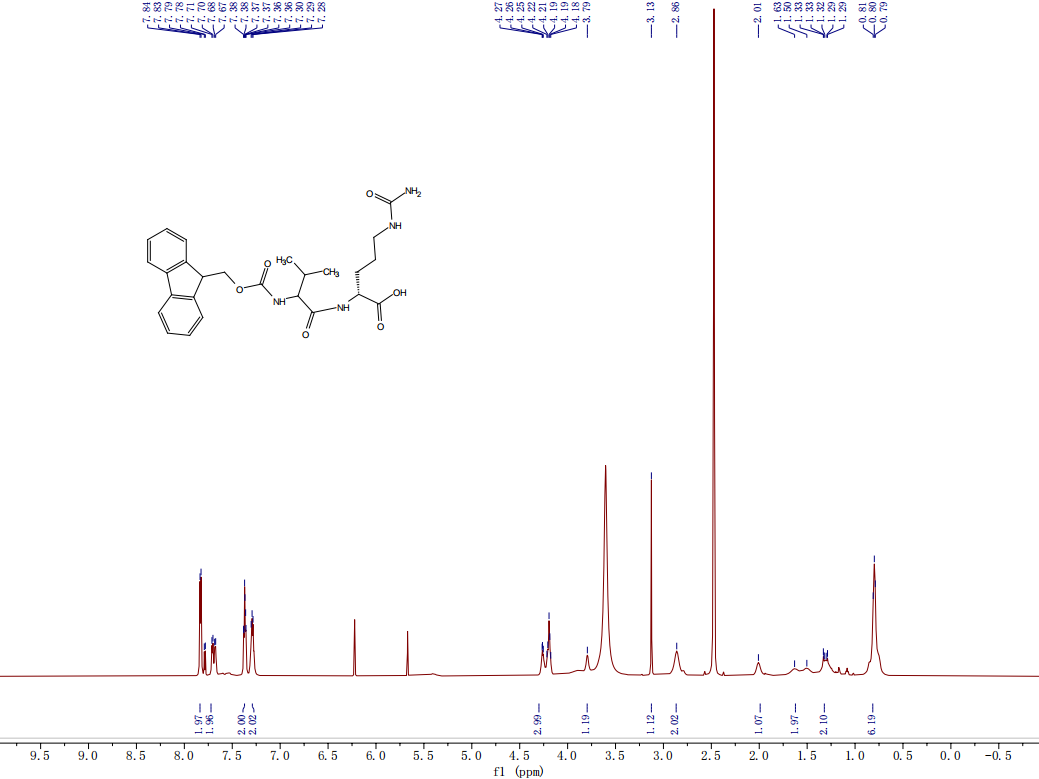


B


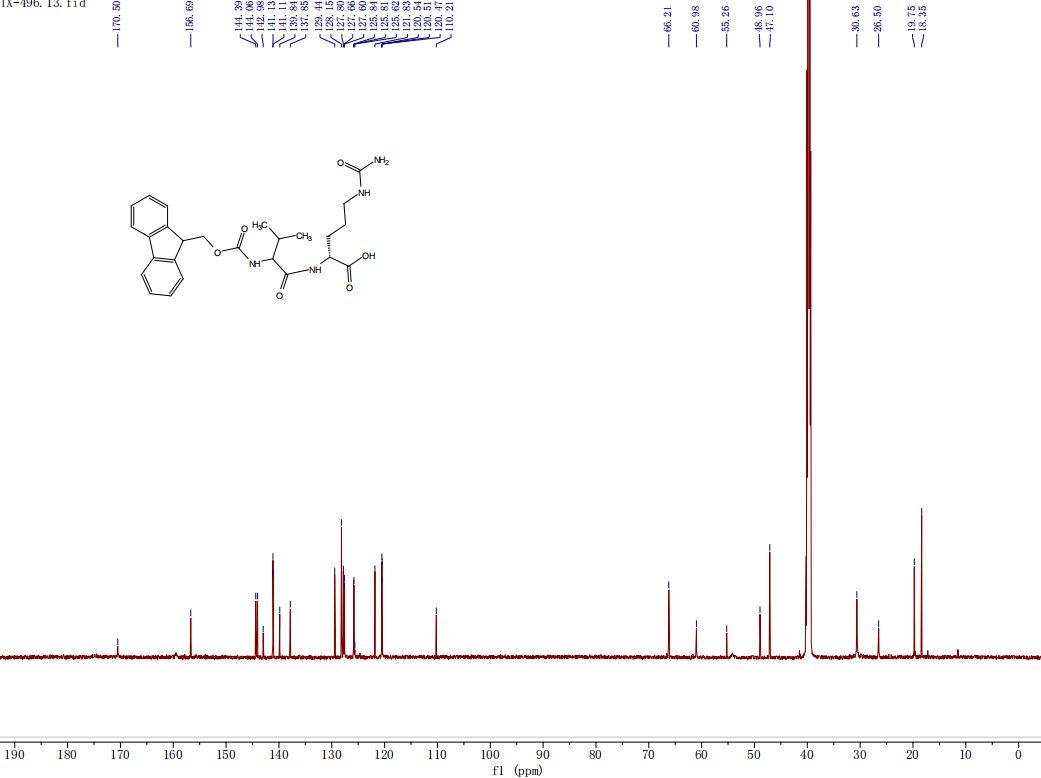


**Figure S15.** ^1^H and ^13^C-NMR spectra of compound 12. White solid, 85.4% yield. A) ^1^H NMR (700 MHz, *d*_6_-DMSO) δ 7.83 (d, *J* = 7.2 Hz, 2H), 7.69 (dd, *J* = 19.8, 7.4 Hz, 2H), 7.37 (td, *J* = 7.5, 2.4 Hz, 2H), 7.29 (dt, *J* = 14.3, 7.4 Hz, 2H), 4.36 – 4.13 (m, 3H), 3.79 (s, 1H), 3.13 (s, 1H), 2.86 (s, 2H), 2.01 (s, 1H), 1.57 (d, *J* = 89.6 Hz, 2H), 1.34 – 1.27 (m, 2H), 0.80 (t, *J* = 7.8 Hz, 6H). B) ^13^C NMR (175 MHz, *d*_6_-DMSO) δ 170.50, 156.69, 144.39, 144.06, 142.98, 141.12 (d, *J* = 4.4 Hz), 139.84, 137.85, 129.44, 128.15, 127.80, 127.66, 127.60, 125.82, 121.83, 120.49 (d, *J* = 7.5 Hz), 110.21, 66.21, 60.98, 55.26, 48.96, 47.10, 30.63, 26.50, 19.75, 18.35.

A


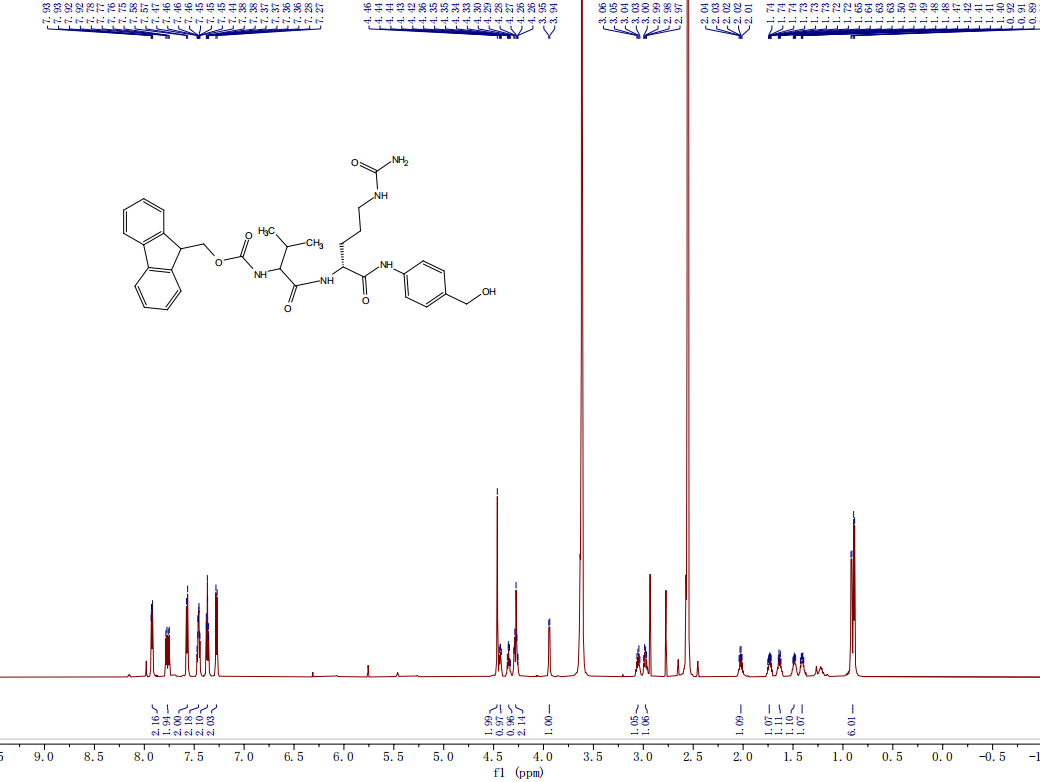


B


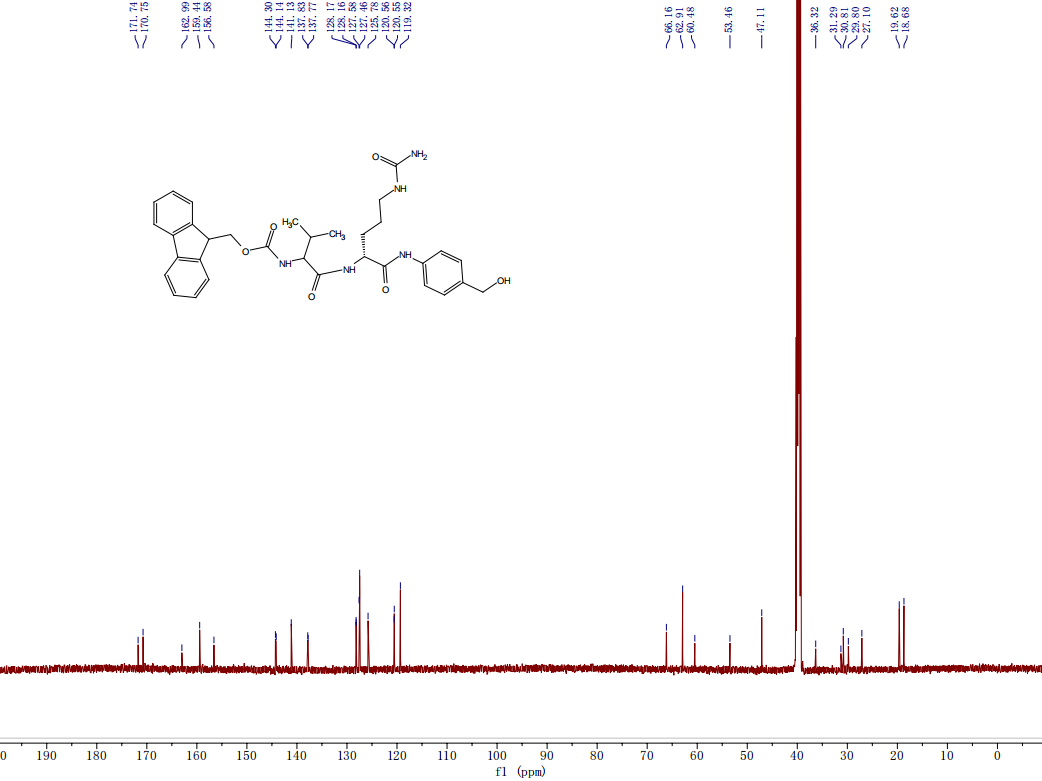


**Figure S16.** ^1^H and ^13^C-NMR spectra of compound 13. White solid, 76.5% yield. A) ^1^H NMR (700 MHz, *d*_6_-DMSO) δ 7.92 (dd, *J* = 7.6, 2.2 Hz, 2H), 7.77 (dd, *J* = 16.6, 7.5 Hz, 2H), 7.57 (d, *J* = 8.2 Hz, 2H), 7.48 – 7.42 (m, 2H), 7.37 (td, *J* = 7.5, 1.3 Hz, 2H), 7.28 (d, *J* = 8.2 Hz, 2H), 4.46 (s, 2H), 4.43 (dd, *J* = 8.7, 5.5 Hz, 1H), 4.35 (dt, *J* = 12.2, 6.1 Hz, 1H), 4.31 – 4.24 (m, 2H), 3.94 (d, *J* = 7.1 Hz, 1H), 3.05 (dt, *J* = 13.7, 6.9 Hz, 1H), 2.98 (dt, *J* = 13.4, 6.7 Hz, 1H), 2.10 – 1.96 (m, 1H), 1.78 – 1.72 (m, 1H), 1.64 (qq, *J* = 9.4, 4.7, 4.2 Hz, 1H), 1.48 (ddq, *J* = 17.4, 12.3, 6.8, 5.7 Hz, 1H), 1.40 (ddt, *J* = 18.3, 14.6, 7.5 Hz, 1H), 0.90 (dd, *J* = 18.9, 6.8 Hz, 6H). B) ^13^C NMR (175 MHz, *d*_6_-DMSO) δ 171.74, 170.75, 162.99, 159.44, 156.58, 144.30, 144.14, 141.13, 137.83, 137.77, 128.17, 128.16, 127.58, 127.46, 125.78, 120.55, 119.32, 66.16, 62.91, 60.48, 53.46, 47.11, 36.32, 31.29, 30.81, 29.80, 27.10, 19.62, 18.68.

A


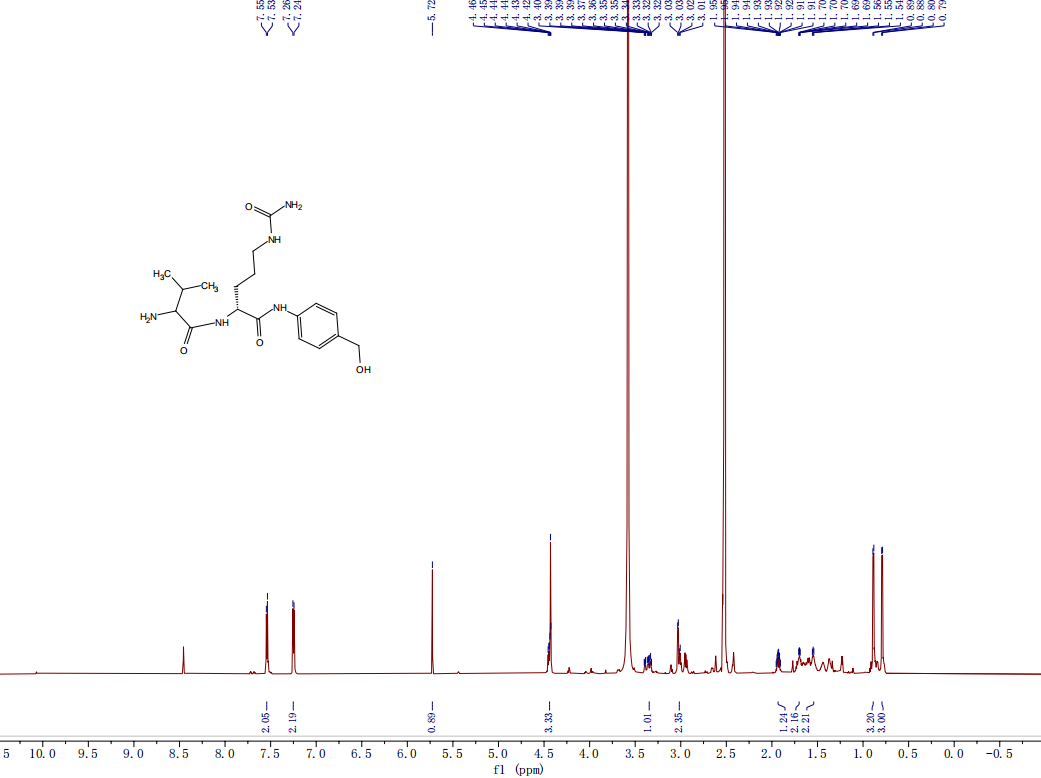


B


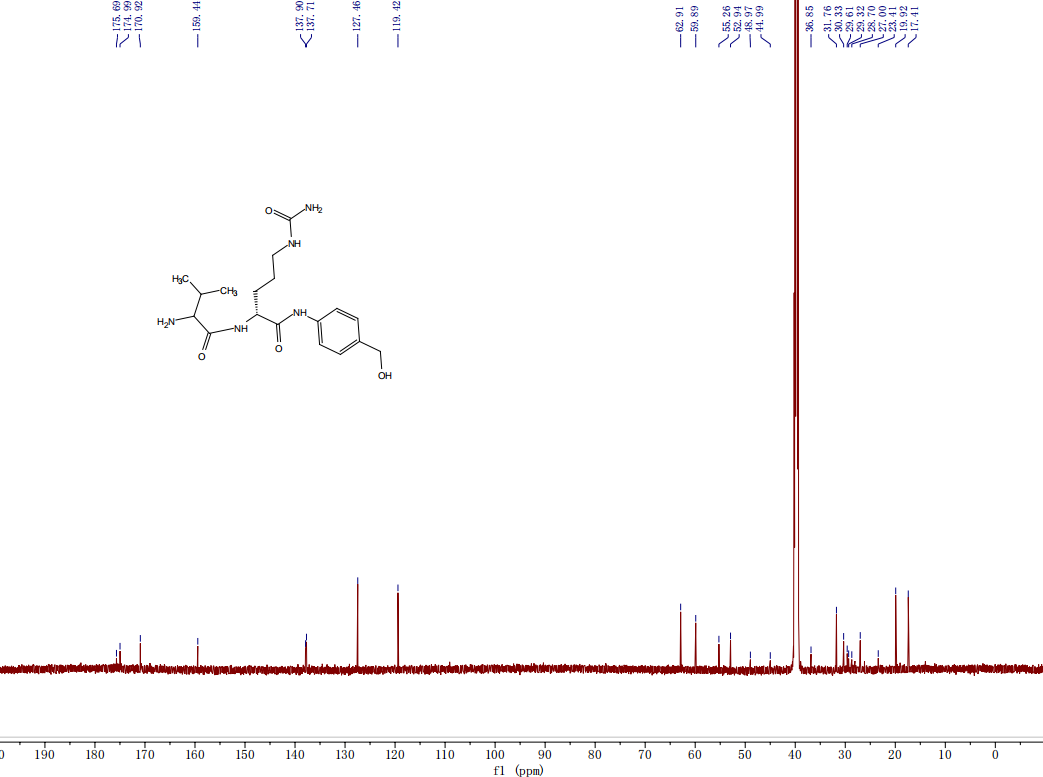


**Figure S17.** ^1^H and ^13^C-NMR spectra of compound 14. White solid, 4.2% yield. A) ^1^H NMR (700 MHz, *d*_6_-DMSO) δ 7.56 – 7.48 (m, 2H), 7.27 – 7.22 (m, 2H), 5.72 (s, 1H), 4.47 – 4.41 (m, 3H), 3.41 – 3.28 (m, 1H), 3.02 (dd, *J* = 13.8, 6.0 Hz, 2H), 1.93 (pd, *J* = 6.9, 4.9 Hz, 1H), 1.77 – 1.64 (m, 2H), 1.62 – 1.52 (m, 2H), 0.89 (d, *J* = 6.9 Hz, 3H), 0.81 – 0.75 (m, 3H). B) ^13^C NMR (175 MHz, *d*_6_-DMSO) δ 175.69, 174.99, 170.92, 159.44, 137.90, 137.71, 127.46, 119.42, 62.91, 59.89, 55.26, 52.94, 48.97, 44.99, 36.85, 31.76, 30.33, 29.32, 28.70, 28.06, 27.00, 23.41, 19.92, 17.41.

A


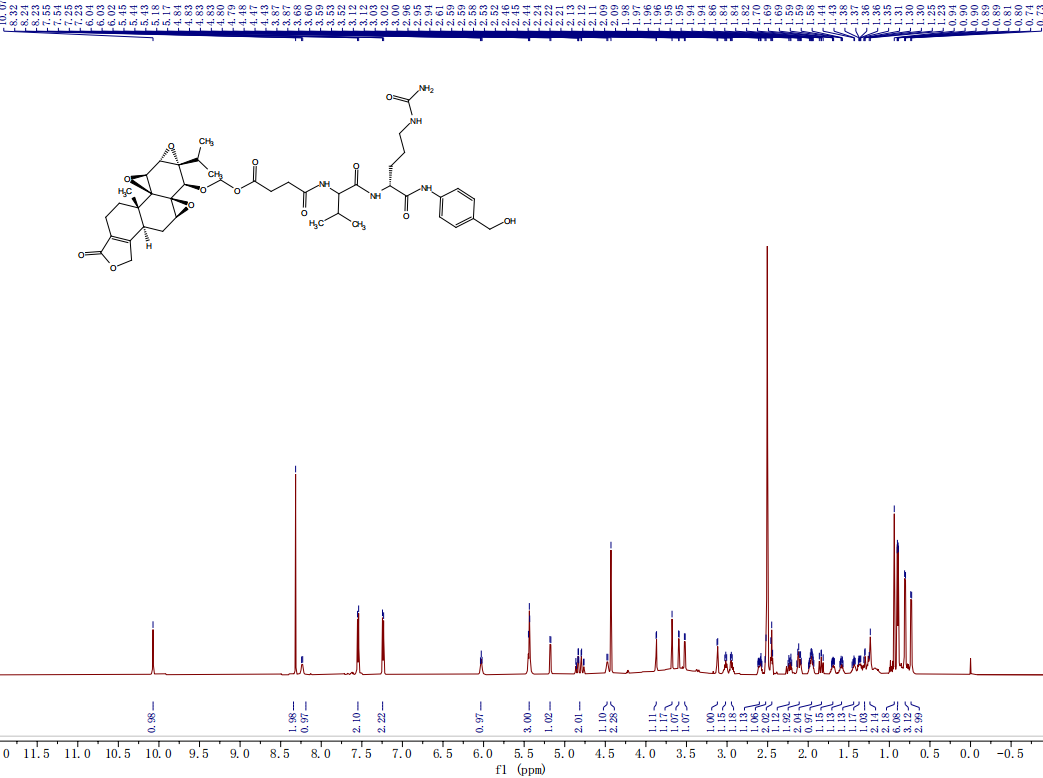


B


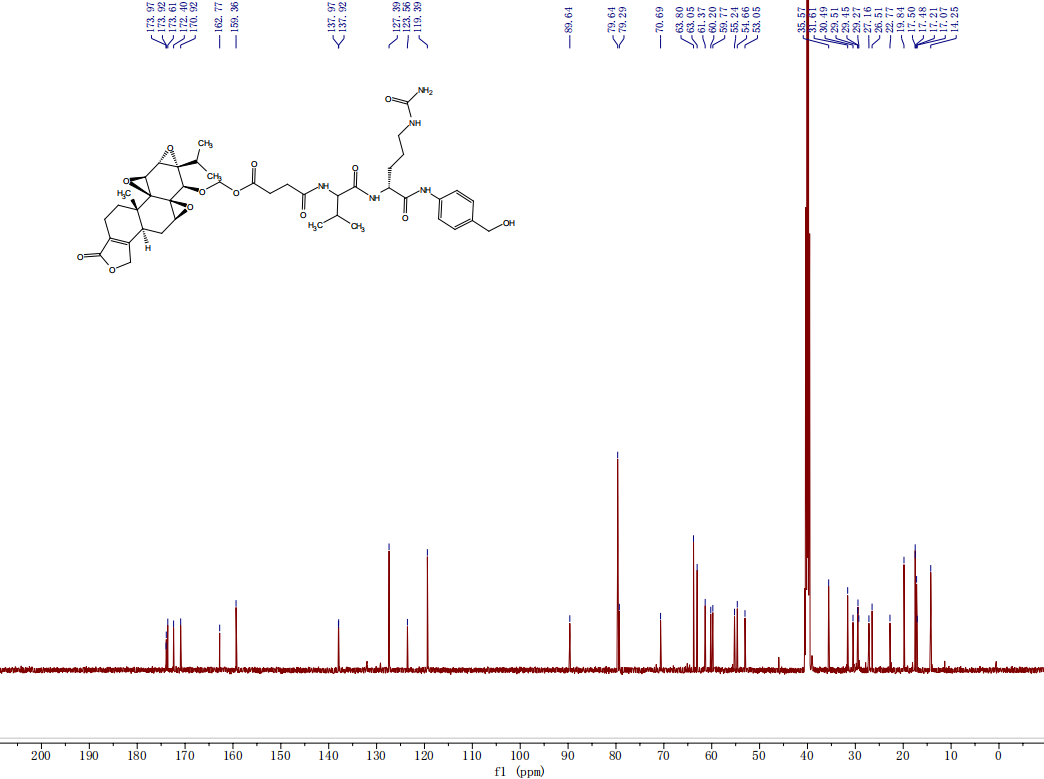


**Figure S18.** ^1^H and ^13^C-NMR spectra of compound 15. Yellow solid, 81.3% yield. A) ^1^H NMR (600 MHz, *d*_6_-DMSO) δ 10.07 (s, 1H), 8.32 (s, 2H), 8.24 (d, *J* = 8.2 Hz, 1H), 7.55 (d, *J* = 8.2 Hz, 2H), 7.24 (d, *J* = 8.3 Hz, 2H), 6.03 (t, *J* = 5.9 Hz, 1H), 5.48 – 5.42 (m, 3H), 5.18 (d, *J* = 6.5 Hz, 1H), 4.86 – 4.76 (m, 2H), 4.47 (d, *J* = 6.8 Hz, 1H), 4.43 (s, 3H), 3.87 (d, *J* = 3.2 Hz, 1H), 3.68 (s, 1H), 3.59 (d, *J* = 3.1 Hz, 1H), 3.52 (d, *J* = 5.7 Hz, 1H), 3.12 (d, *J* = 5.0 Hz, 1H), 3.05 – 2.98 (m, 1H), 2.98 – 2.90 (m, 1H), 2.62 – 2.56 (m, 1H), 2.53 (d, *J* = 7.3 Hz, 1H), 2.45 (t, *J* = 6.6 Hz, 2H), 2.23 (dt, *J* = 15.0, 5.9 Hz, 1H), 2.11 (dq, *J* = 11.8, 6.1, 5.1 Hz, 2H), 1.96 (dtd, *J* = 13.8, 8.1, 6.8, 3.9 Hz, 2H), 1.84 (dd, *J* = 15.1, 13.2 Hz, 1H), 1.74 – 1.65 (m, 1H), 1.59 (dq, *J* = 9.3, 4.5 Hz, 1H), 1.44 (dq, *J* = 9.1, 5.5, 4.8 Hz, 1H), 1.41 – 1.30 (m, 1H), 1.25 (s, 1H), 1.23 (s, 1H), 0.94 (s, 2H), 0.90 (dd, *J* = 6.9, 1.6 Hz, 6H), 0.81 (d, *J* = 6.8 Hz, 3H), 0.73 (d, *J* = 6.9 Hz, 3H). B) ^13^C NMR (150 MHz, *d*_6_-DMSO) δ 173.97, 173.92, 173.61, 172.40, 170.92, 162.77, 159.36, 137.94, 127.39, 123.56, 119.39, 89.64, 79.64, 79.29, 70.69, 63.80, 63.05, 61.37, 60.20, 59.77, 55.24, 54.66, 53.05, 35.57, 31.61, 30.49, 29.51, 29.45, 29.27, 27.16, 26.51, 22.77, 19.84, 17.49, 17.21, 17.07, 14.25.

A


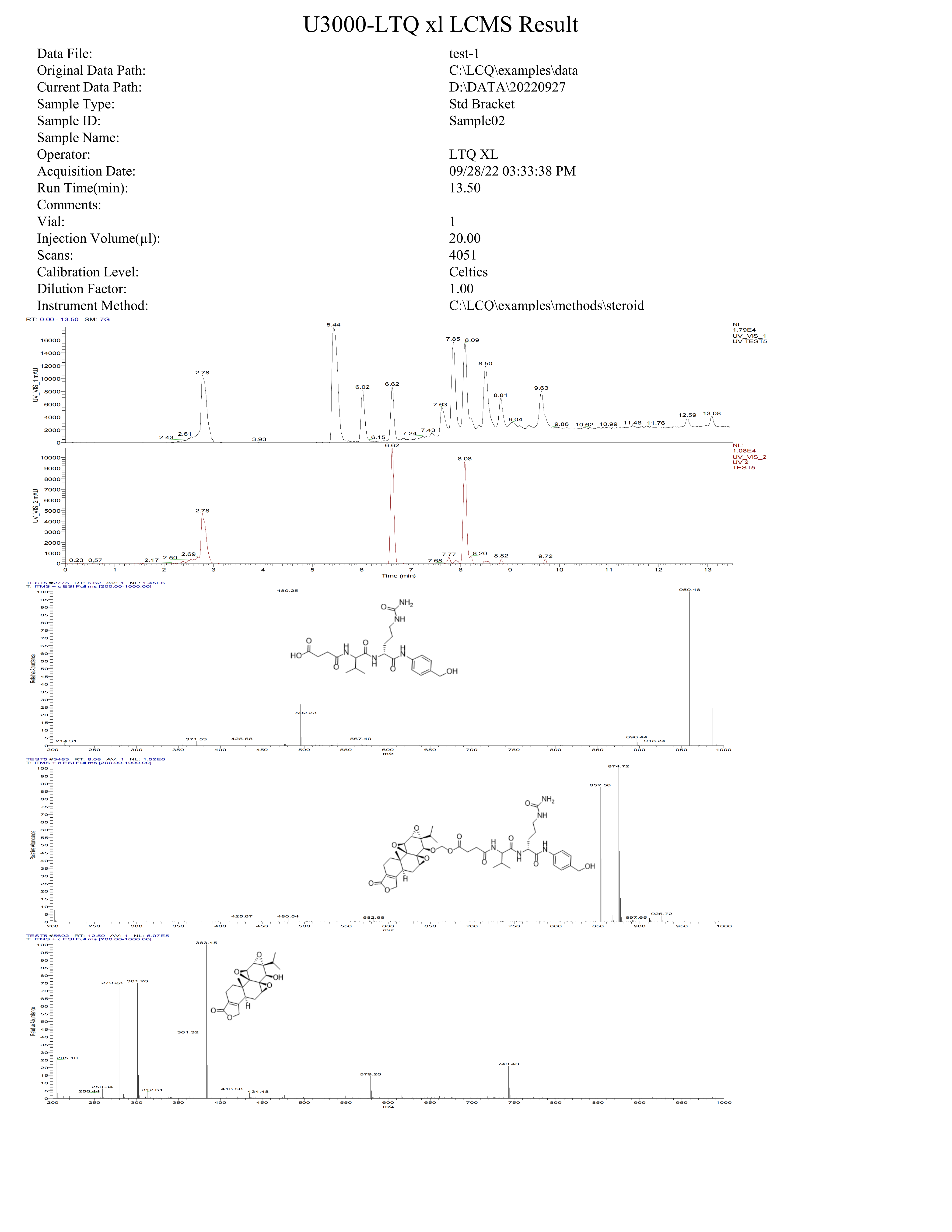


B


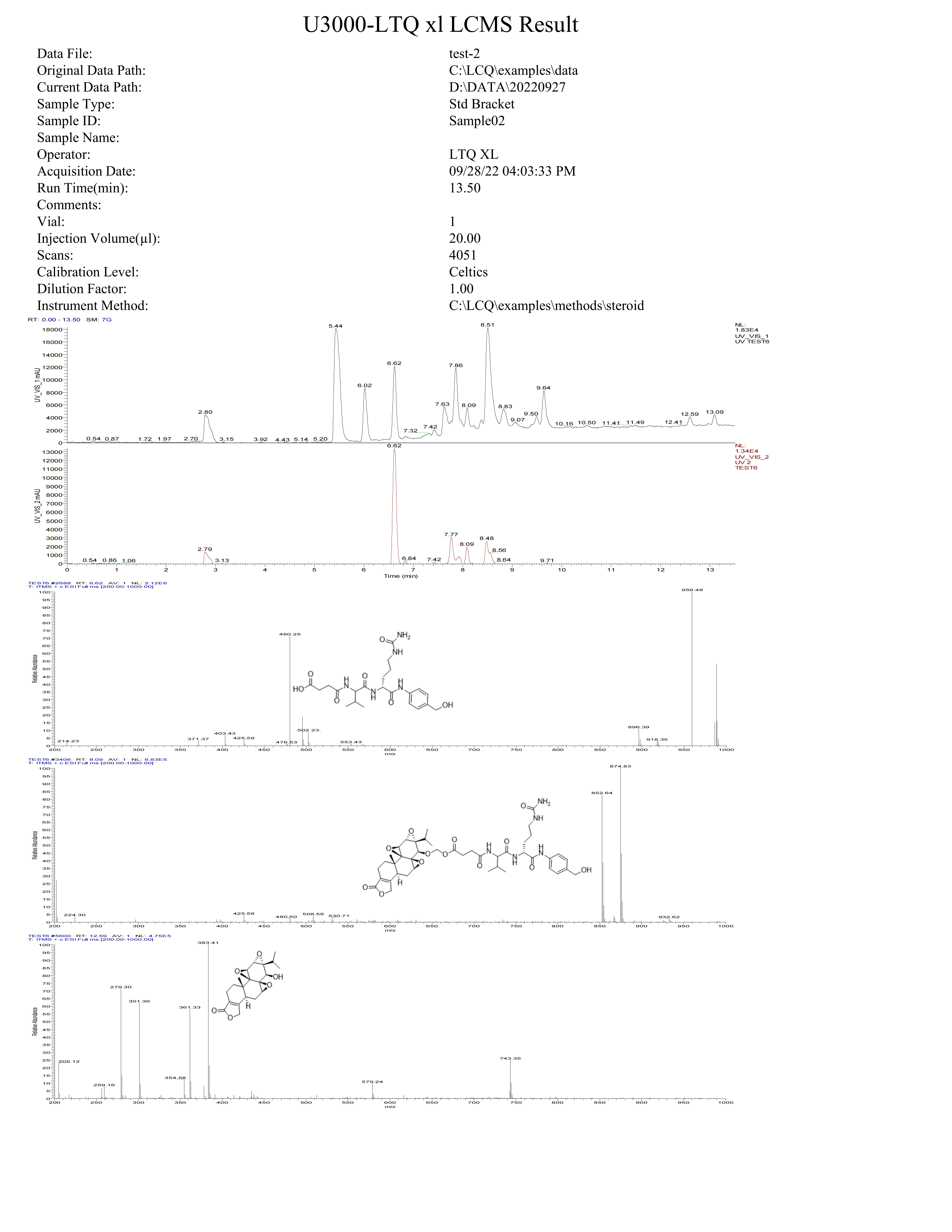


**Figure S19.** Decomposition of dipeptide bond-triptolide conjugate under acidic condition. A) Decomposition of dipeptide bond-triptolide conjugate under acidic condition for 5 min. B) Decomposition of dipeptide bond-triptolide conjugate under acidic condition for 20 min.

A


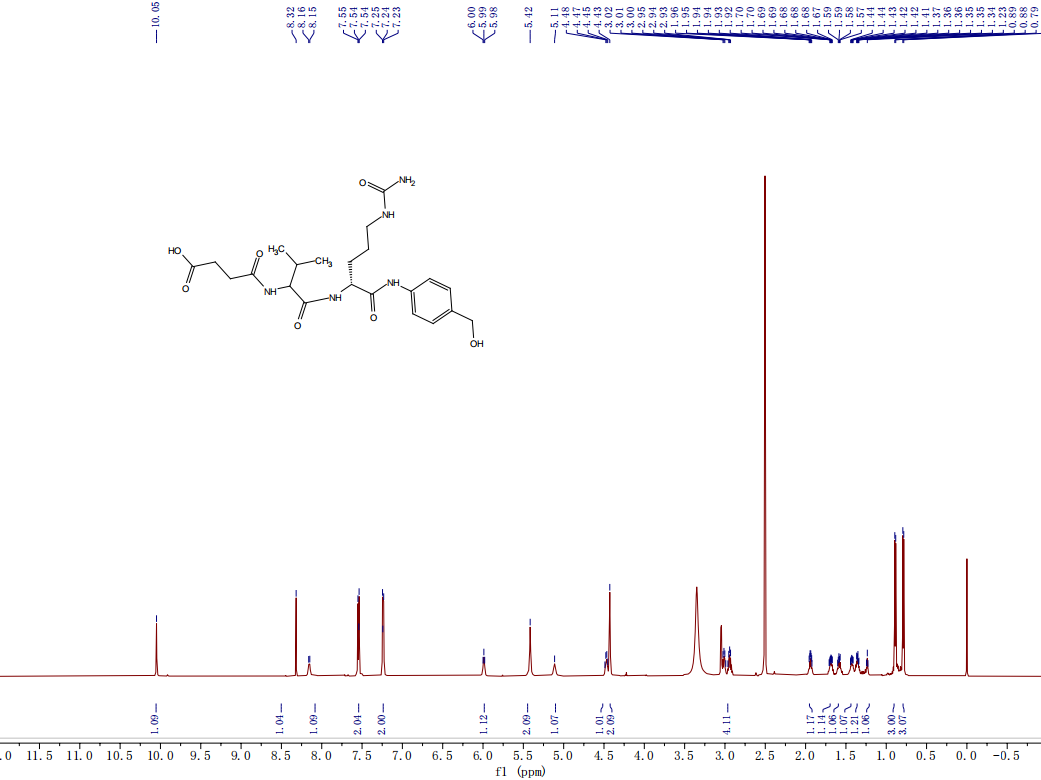


B


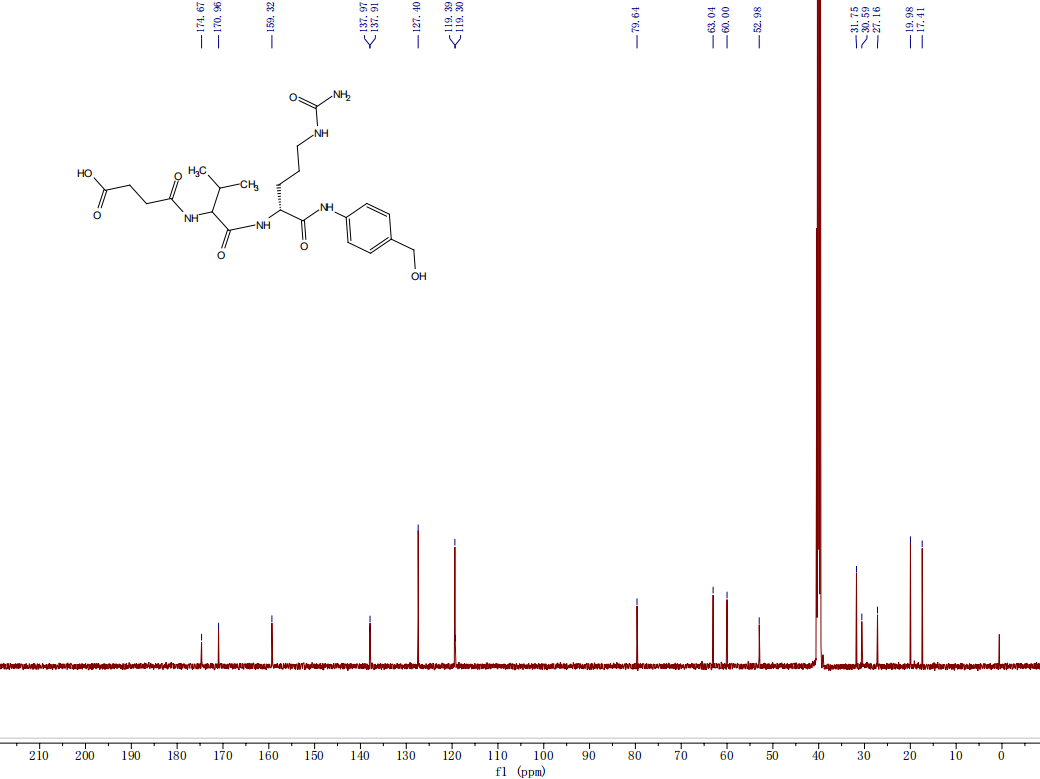


**Figure S20.** ^1^H and ^13^C-NMR spectra of dipeptide bond derivative. A) ^1^H NMR (600 MHz, *d*_6_-DMSO) δ 10.05 (s, 1H), 8.32 (s, 1H), 8.15 (d, *J* = 8.1 Hz, 1H), 7.64 – 7.49 (m, 2H), 7.24 (d, *J* = 8.5 Hz, 2H), 5.99 (t, *J* = 6.0 Hz, 1H), 5.42 (s, 2H), 5.11 (s, 1H), 4.47 (q, *J* = 6.8 Hz, 1H), 4.43 (s, 2H), 3.03 – 2.92 (m, 4H), 1.94 (td, *J* = 7.0, 5.0 Hz, 1H), 1.71 – 1.66 (m, 1H), 1.58 (dd, *J* = 9.2, 4.7 Hz, 1H), 1.43 (td, *J* = 6.1, 5.3, 2.5 Hz, 1H), 1.35 (td, *J* = 7.0, 3.6 Hz, 1H), 1.24 (d, *J* = 5.9 Hz, 1H), 0.89 (d, *J* = 6.9 Hz, 3H), 0.79 (d, *J* = 6.8 Hz, 3H). B) ^13^C NMR (150 MHz, *d*_6_-DMSO) δ 174.67, 170.96, 159.32, 137.97, 137.91, 127.40, 119.39, 119.30, 79.64, 63.04, 60.00, 52.98, 31.75, 30.59, 27.16, 19.98, 17.41.

**Figure S21.** Synthesis of the aptamer-triptolide conjugate with the vinyl ester linker. Note for reagents and conditions: (vii) allyl bromide, NaHCO_3_, DMF; (viii) triptolide, NMM, ACN; (ix) morpholine, Pd(PPh_3_)_4_, THF. NMM = *N*-methylmorpholine, ACN = Acetonitrile, THF = Tetrahydrofuran. Treated with allyl bromide, propiolic acid (**7**) was converted into **8** upon formation of the ester group with the NaHCO_3_ assistant. Michael addition of **8** with TP under the catalysis of N-methylmorpholine afforded exclusively TP derivative **9** containing an acid-labile vinyl linker. The free carboxyl group of **10** was obtained after deprotection of the allyl group via utilizing Pd(PPh_3_)_4_ and morpholine.

A


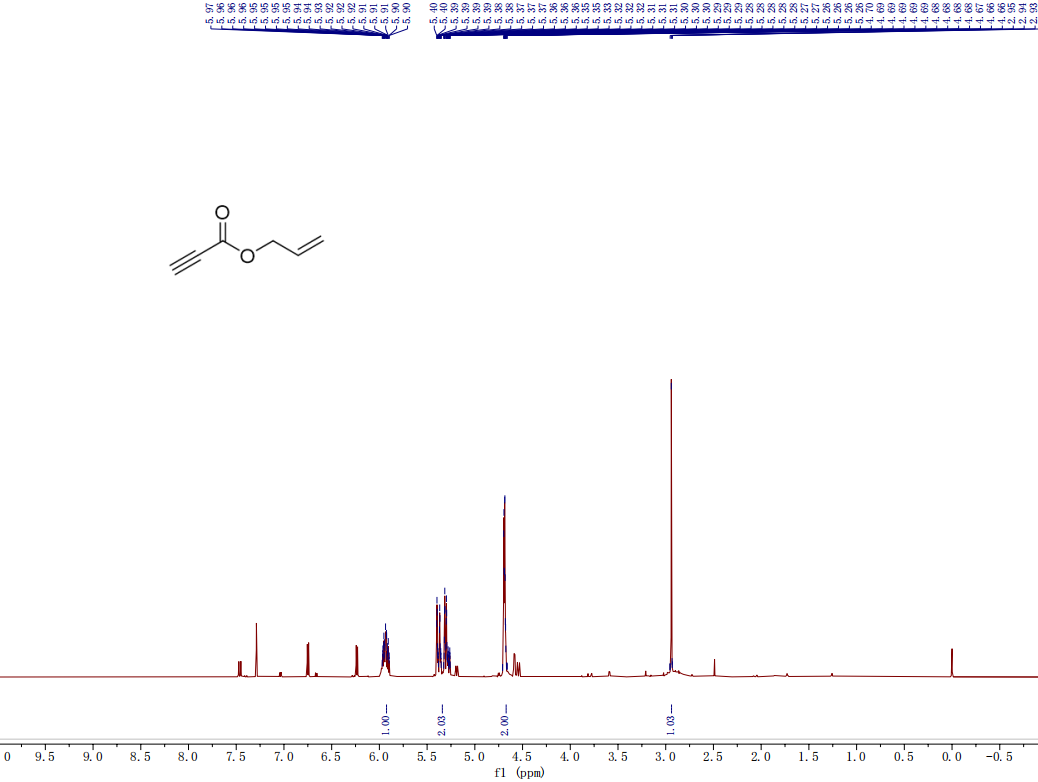


B


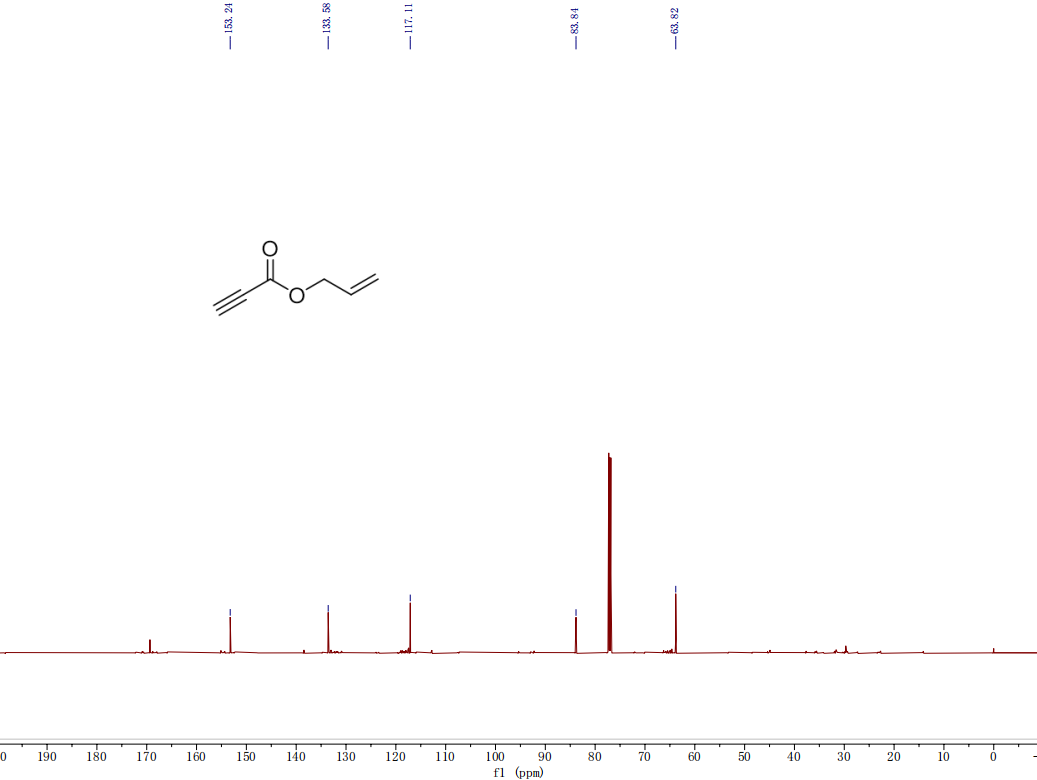


**Figure 22.** ^1^H and ^13^C-NMR spectra of compound 8. Yellow oil, 90.8% yield. A) ^1^H NMR (600 MHz, CDCl_3_) δ 5.97 – 5.88 (m, 1H), 5.43 – 5.25 (m, 2H), 4.69 (ddd, *J* = 7.1, 3.6, 2.2 Hz, 2H), 2.94 (s, 1H). B) ^13^C NMR (150 MHz, CDCl_3_) δ 153.24, 133.58, 117.11, 83.84, 63.82.

A


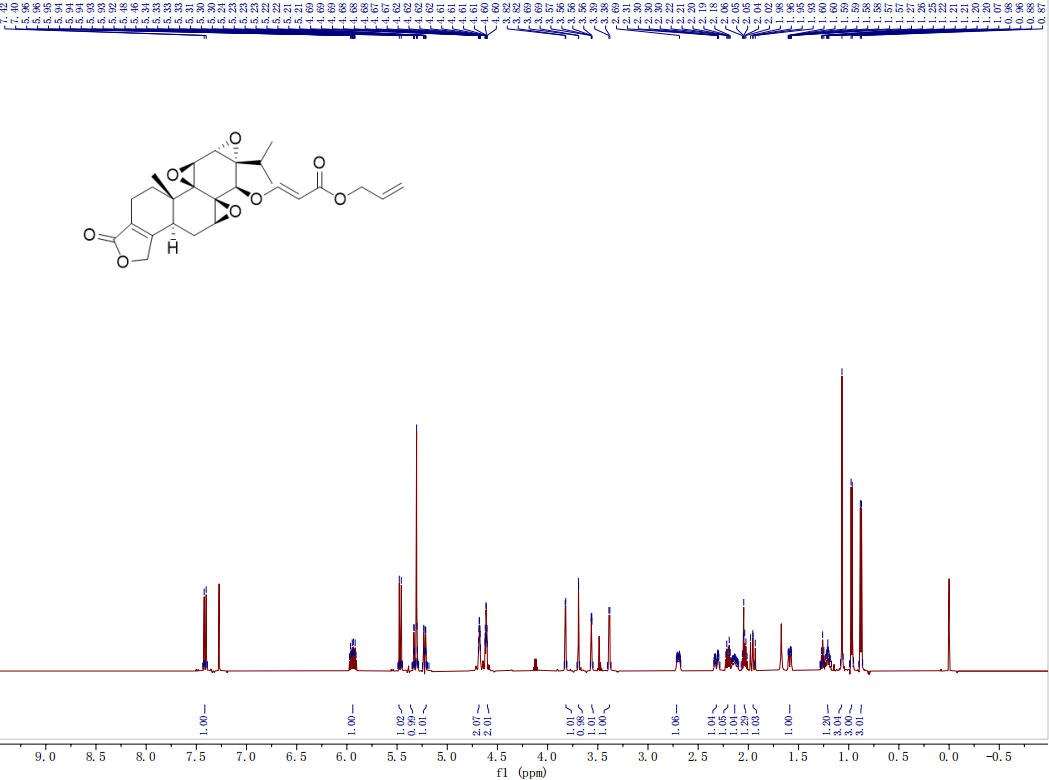


B


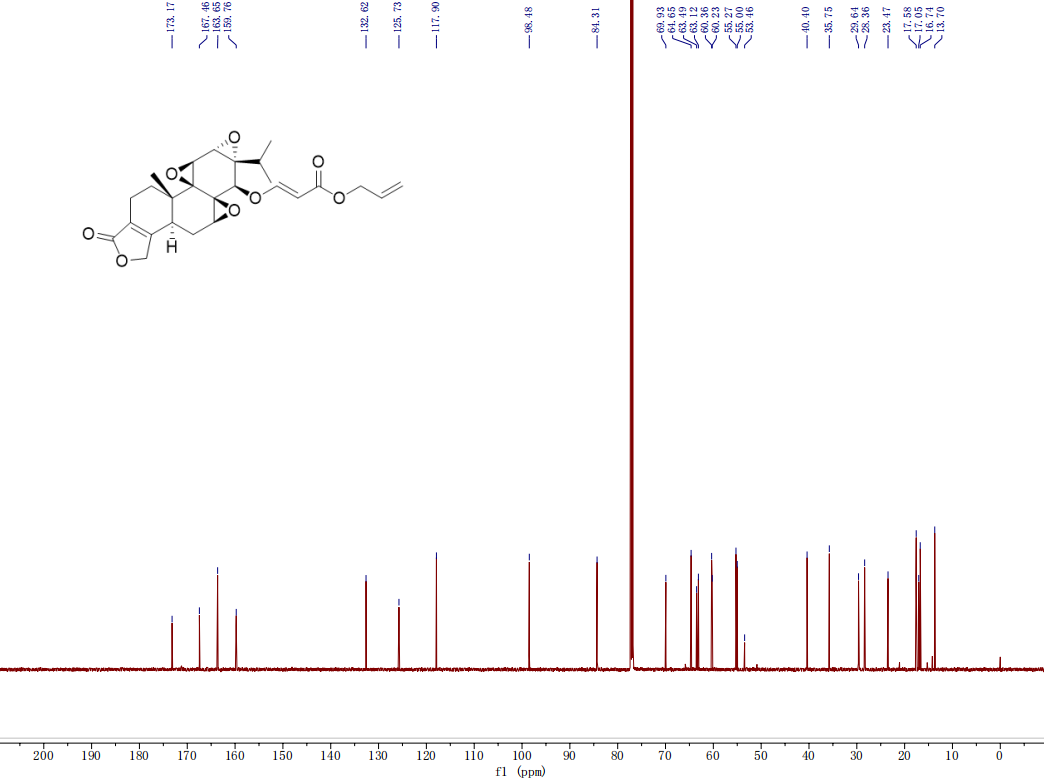


**Figure S23.** ^1^H and ^13^C-NMR spectra of compound 9. White solid, 51.1% yield. A) ^1^H NMR (600 MHz, CDCl_3_) δ 7.41 (d, *J* = 12.1 Hz, 1H), 5.94 (ddt, *J* = 17.2, 10.4, 5.7 Hz, 1H), 5.47 (d, *J* = 12.2 Hz, 1H), 5.35 – 5.28 (m, 1H), 5.22 (dq, *J* = 10.4, 1.3 Hz, 1H), 4.68 (ddt, *J* = 5.2, 3.3, 1.6 Hz, 2H), 4.61 (ddt, *J* = 5.9, 4.5, 1.4 Hz, 2H), 3.82 (d, *J* = 3.2 Hz, 1H), 3.69 (d, *J* = 0.8 Hz, 1H), 3.56 (dd, *J* = 3.2, 0.9 Hz, 1H), 3.38 (d, *J* = 5.6 Hz, 1H), 2.75 – 2.63 (m, 1H), 2.32 (dtd, *J* = 18.0, 3.8, 1.9 Hz, 1H), 2.20 (dt, *J* = 14.8, 5.8 Hz, 1H), 2.17 – 2.09 (m, 1H), 2.07 – 2.01 (m, 1H), 1.95 (dd, *J* = 14.8, 13.4 Hz, 1H), 1.59 (ddd, *J* = 12.4, 5.6, 1.6 Hz, 1H), 1.29 – 1.17 (m, 1H), 1.07 (s, 3H), 0.97 (d, *J* = 7.0 Hz, 3H), 0.88 (d, *J* = 6.9 Hz, 3H). B) ^13^C NMR (150 MHz, CDCl_3_) δ 173.17, 167.46, 163.65, 159.76, 132.62, 125.73, 117.90, 98.48, 84.31, 69.93, 64.65, 63.49, 63.12, 60.36, 60.23, 55.27, 55.00, 53.46, 40.40, 35.75, 29.64, 28.36, 23.47, 17.58, 17.05, 16.74, 13.70.

A


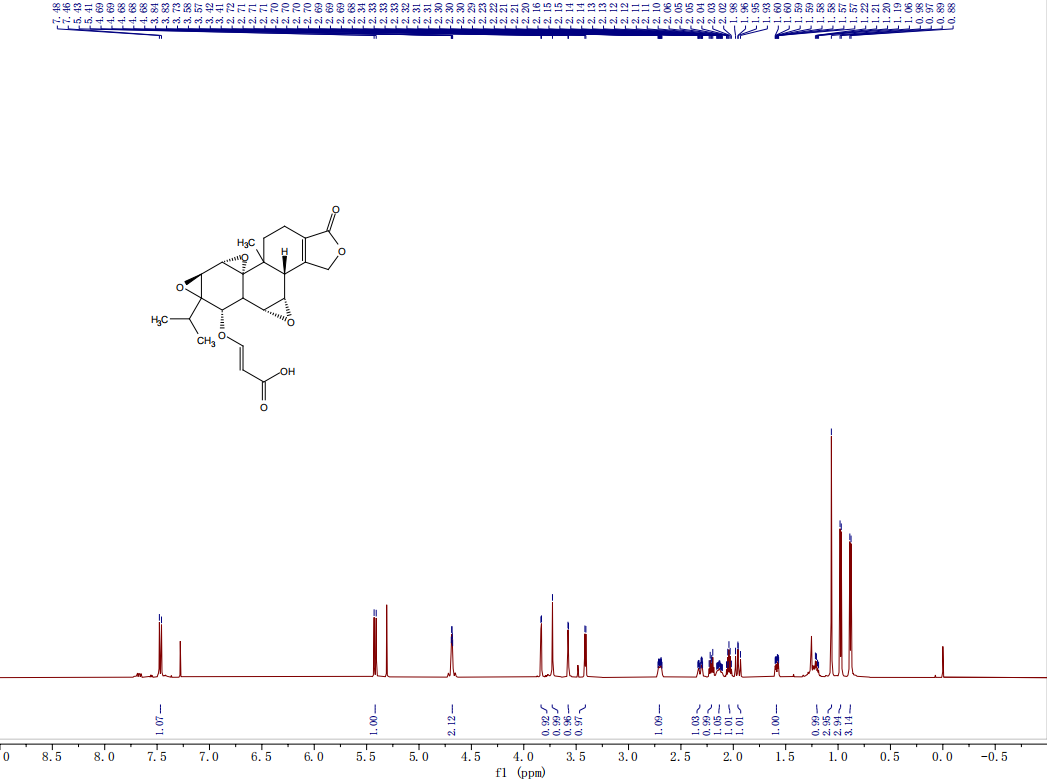


B


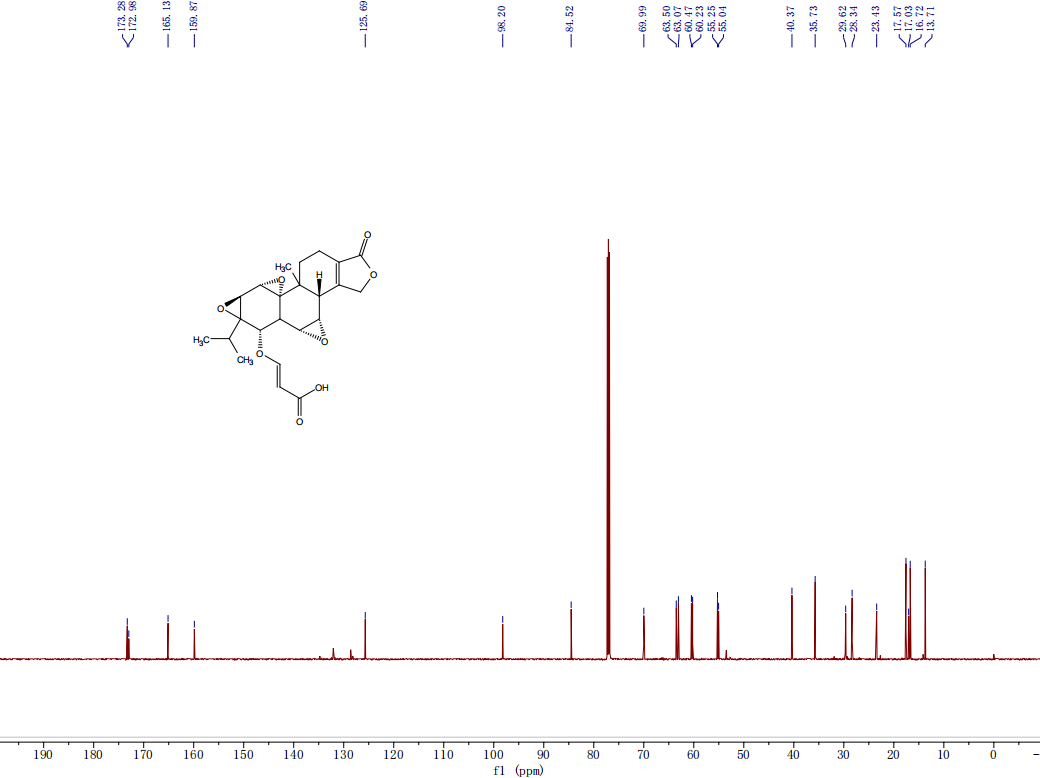


**Figure S24.** ^1^H and ^13^C-NMR spectra of compound 10. White solid, 32.8% yield. A) ^1^H NMR (600 MHz, CDCl_3_): δ = 7.47 (d, *J* = 12.1 Hz, 1H), 5.42 (d, *J* = 12.1 Hz, 1H), 4.75 – 4.60 (m, 2H), 3.83 (d, *J* = 3.2 Hz, 1H), 3.73 (s, 1H), 3.58 (d, *J* = 3.1 Hz, 1H), 3.41 (d, *J* = 5.6 Hz, 1H), 2.74 – 2.66 (m, 1H), 2.32 (ddt, *J* = 18.2, 6.2, 2.1 Hz, 1H), 2.21 (dt, *J* = 14.8, 5.8 Hz, 1H), 2.14 (ttd, *J* = 11.1, 6.2, 5.3, 2.8 Hz, 1H), 2.04 (p, *J* = 6.9 Hz, 1H), 1.96 (dd, *J* = 14.8, 13.5 Hz, 1H), 1.58 (ddd, *J* = 12.6, 5.5, 1.5 Hz, 1H), 1.24 – 1.17 (m, 1H), 1.06 (s, 3H), 0.98 (d, *J* = 7.0 Hz, 3H), 0.88 (d, *J* = 6.9 Hz, 3H). B) ^13^C NMR (150 MHz, CDCl_3_): δ = 173.28, 172.99, 165.13, 159.87, 125.69, 98.21, 84.52, 69.99, 63.50, 63.07, 60.47, 60.23, 55.25, 55.04, 40.37, 35.73, 29.66, 28.33, 23.42, 17.57, 17.03, 16.72, 13.71.

A


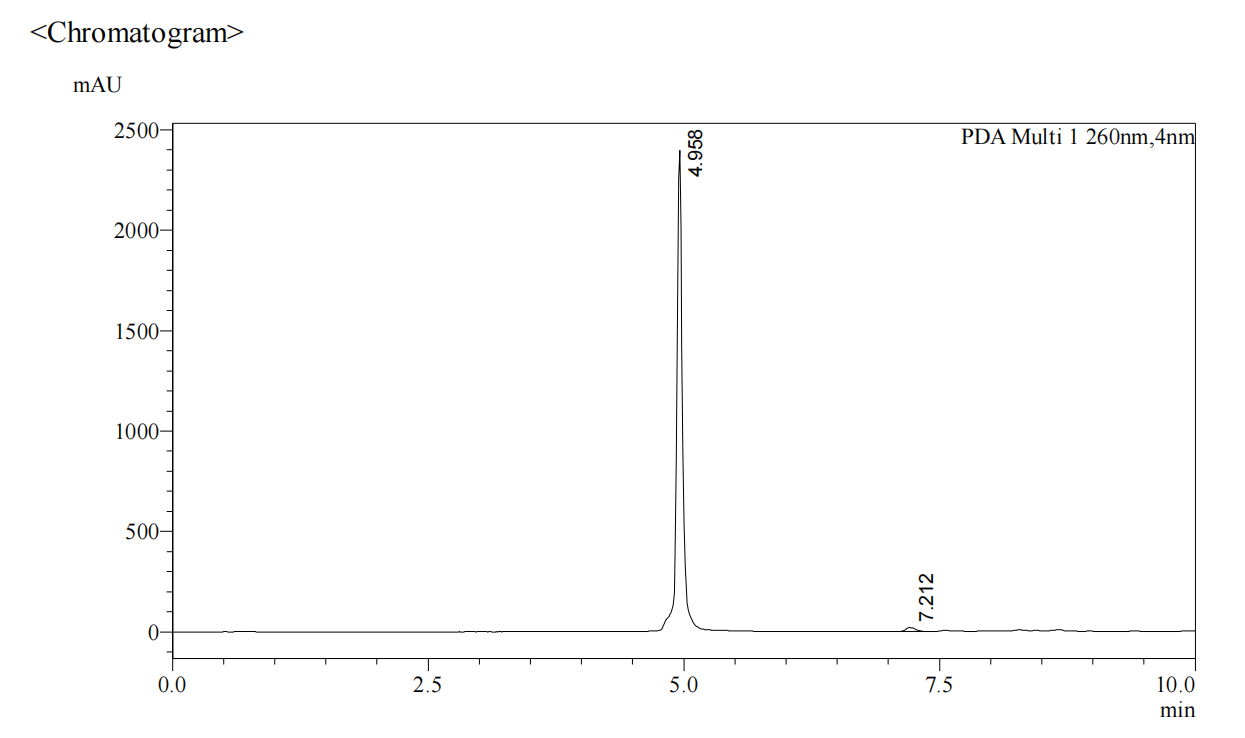


B


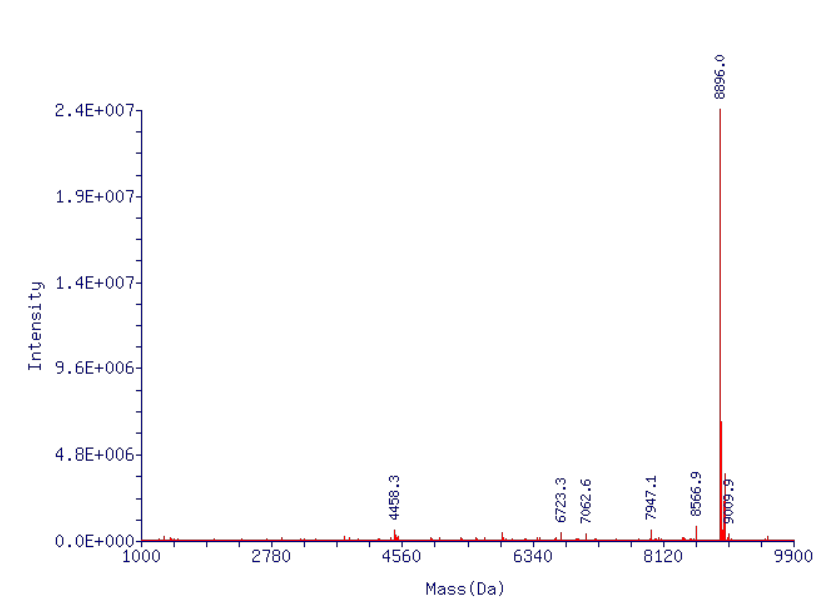


**Figure S25.** HPLC chromatogram and MS spectrum of 6e. A) The HPLC chromatogram of the AS-TP(V) conjugate. B) MS (ESI): [M+H]+: calculated 8896.5, found 8896.0.


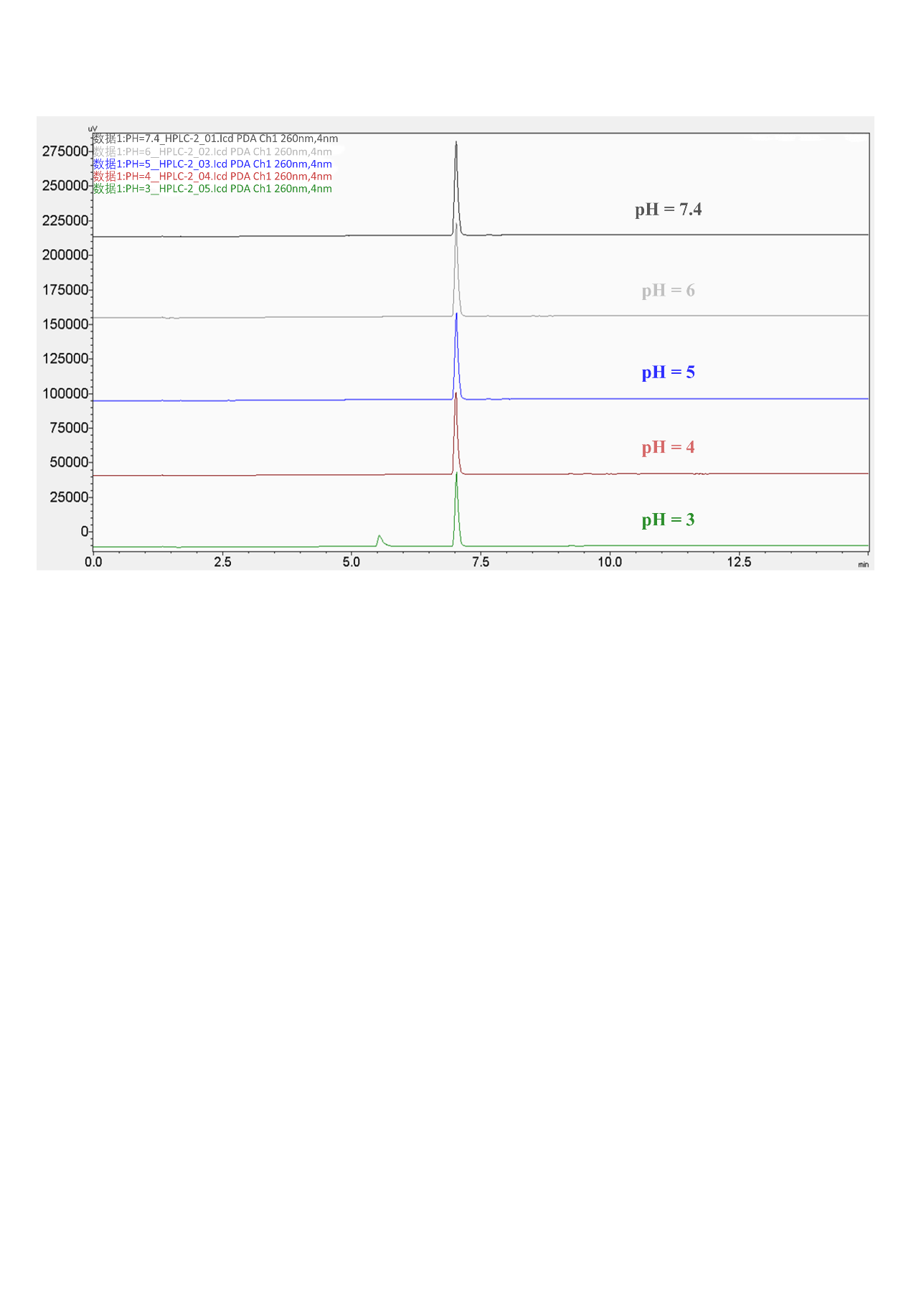
**Figure S26.** The cleavage of the vinyl ester linker in AS-TP(V) was determined by HPLC at different pH (pH = 3, 4, 5, 6 and 7.4)


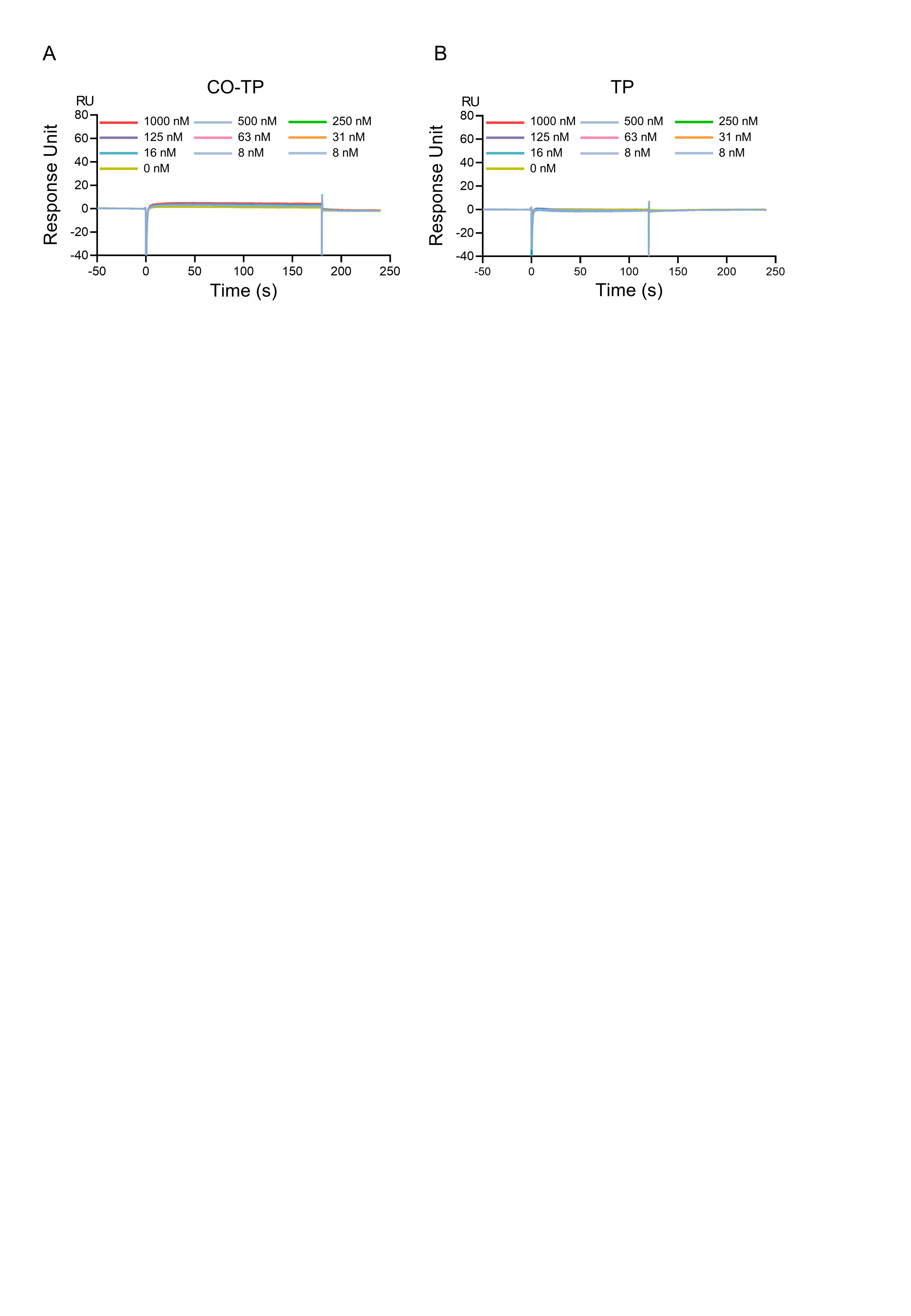


**Figure S**27. The affinity between a variety of compounds and the NuP. A-B) SPR determination of the affinity of (A) CO-TP and (B) TP to the NuP.


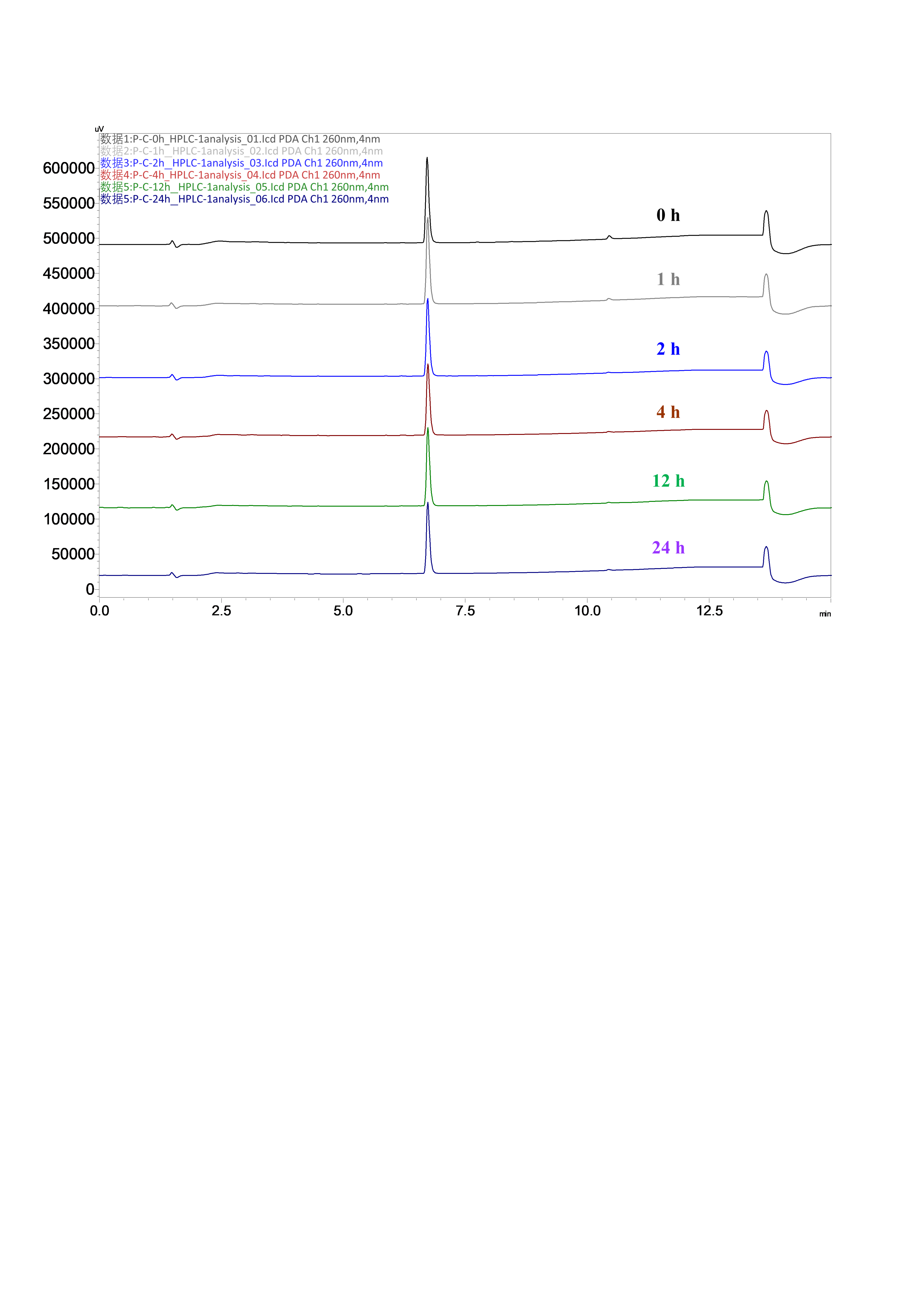


**Figure S28.** Serum stability of AS-TP measured by HPLC. The stability of AS-TP structure in PBS containing 50% serum at different time points (t = 0 h, 1 h, 2 h, 4 h, 12 h and 24 h).


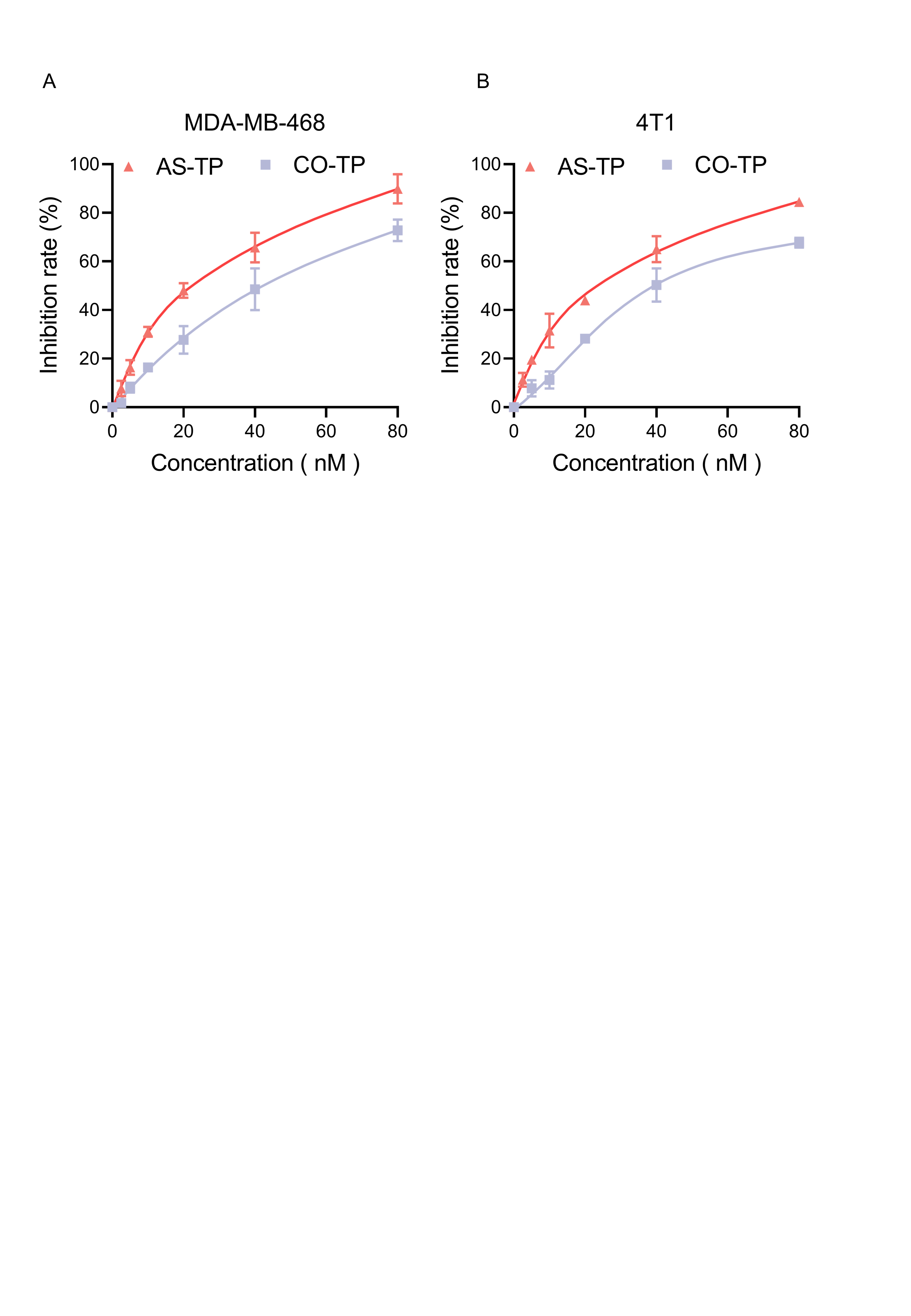


**Figure S29.** Cytotoxicity of AS-TP and CO-TP on TNBC cells. A) MTT proliferation assay was used to determine the effect of AS-TP and CO-TP on MDA-MB-468 cells. B) MTT proliferation assay was used to determine the effect of AS-TP and CO-TP on 4T1 cells.


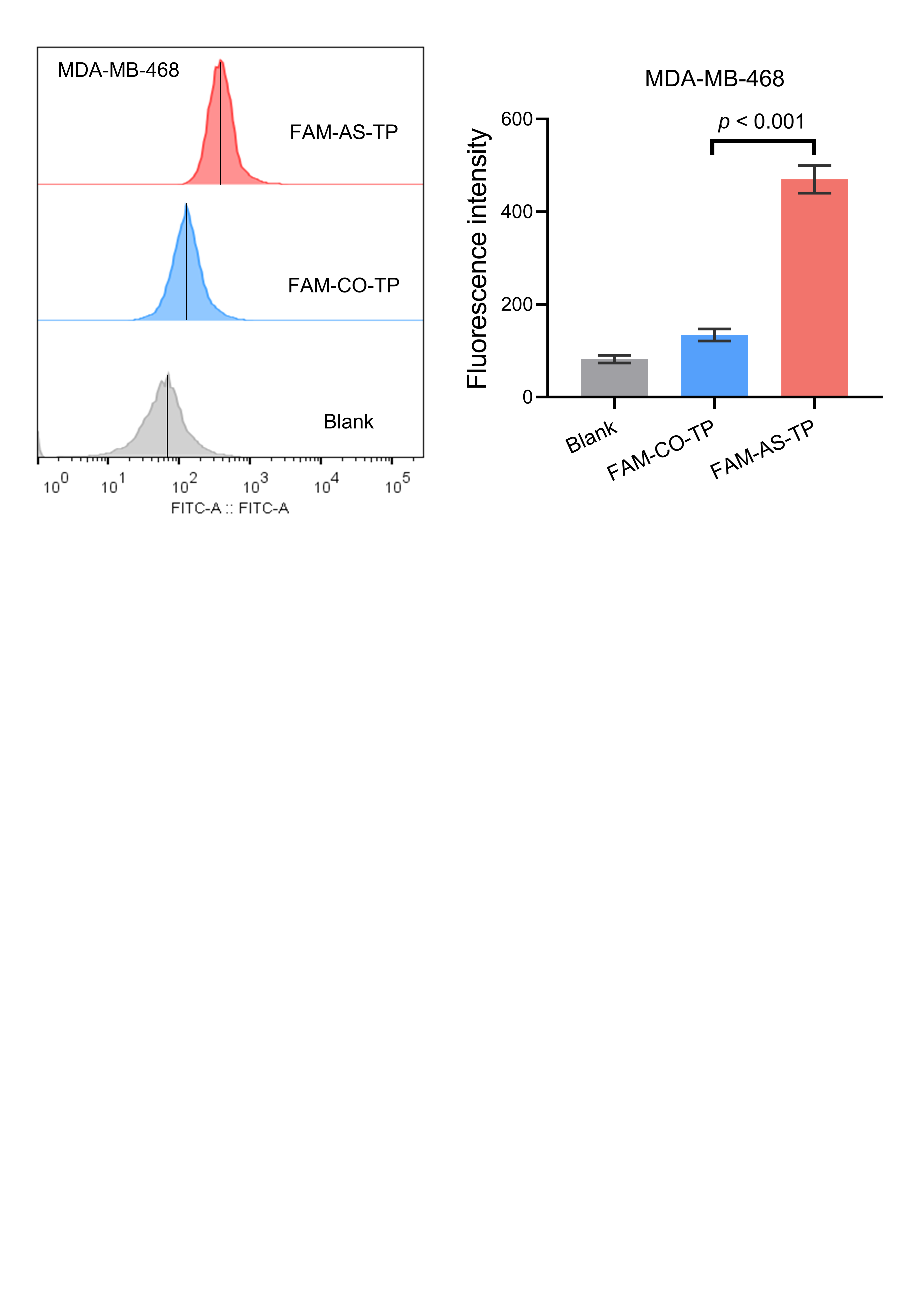


**Figure S30.** The uptake of FAM-AS-TP and FAM-CO-TP by MDA-MB-468 cells was determined by flow cytometry.


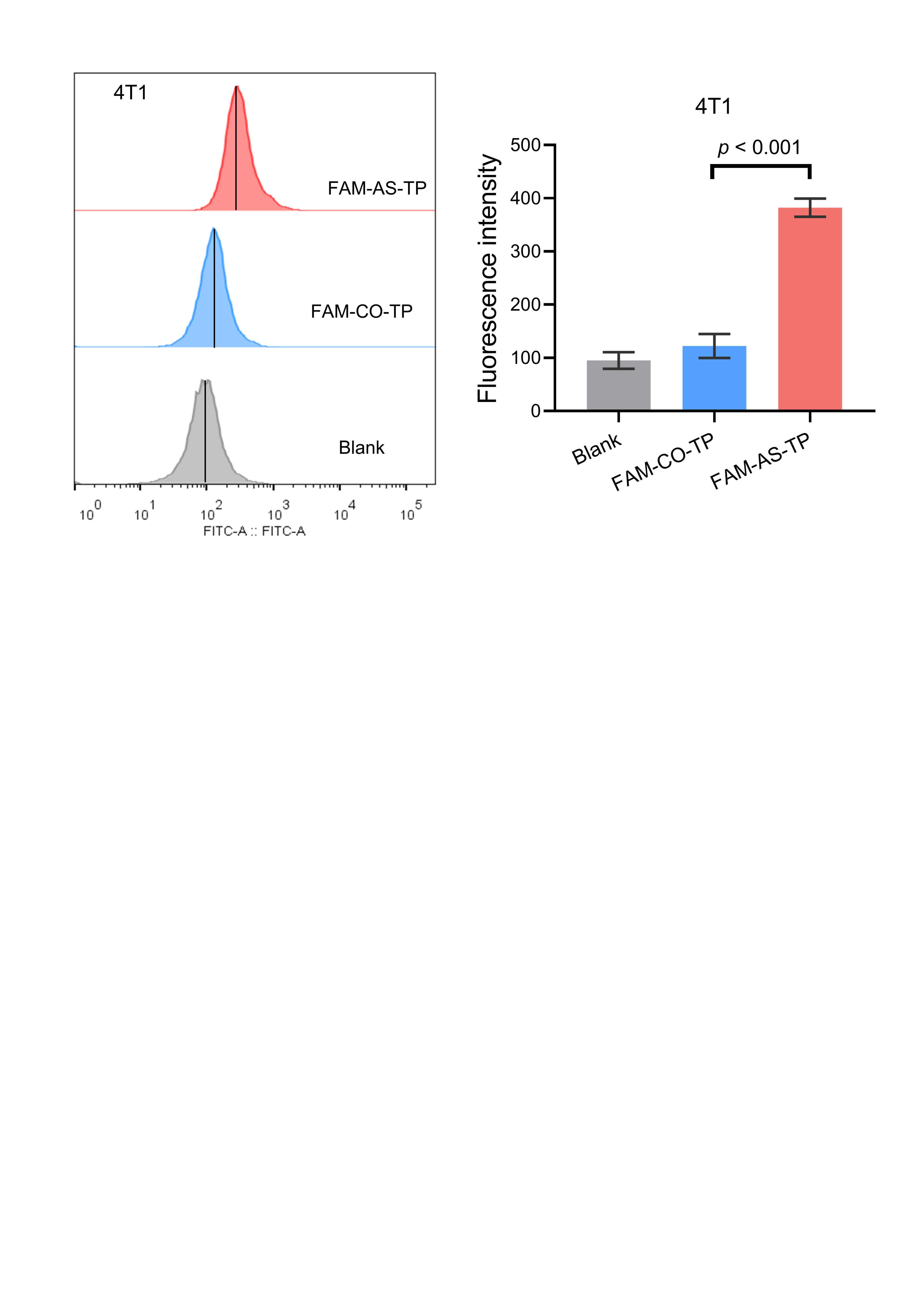


**Figure S31.** The uptake of FAM-AS-TP and FAM-CO-TP by 4T1 cells was determined by flow cytometry.


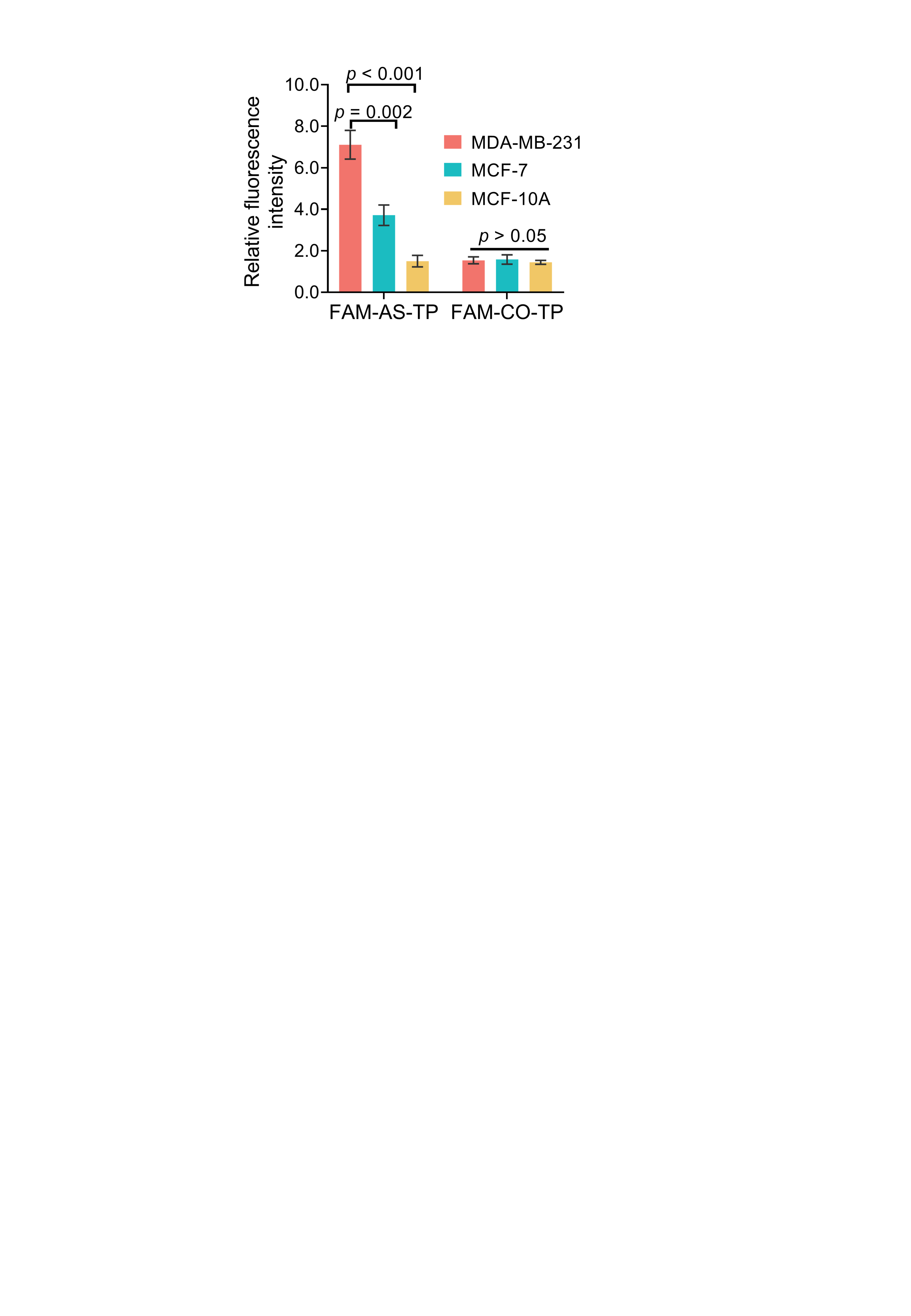


**Figure S32.** Uptake quantization of FAM-AS-TP and FAM-CO-TP in MDA-MB-231, MCF-7 and MCF-10A cell lines.


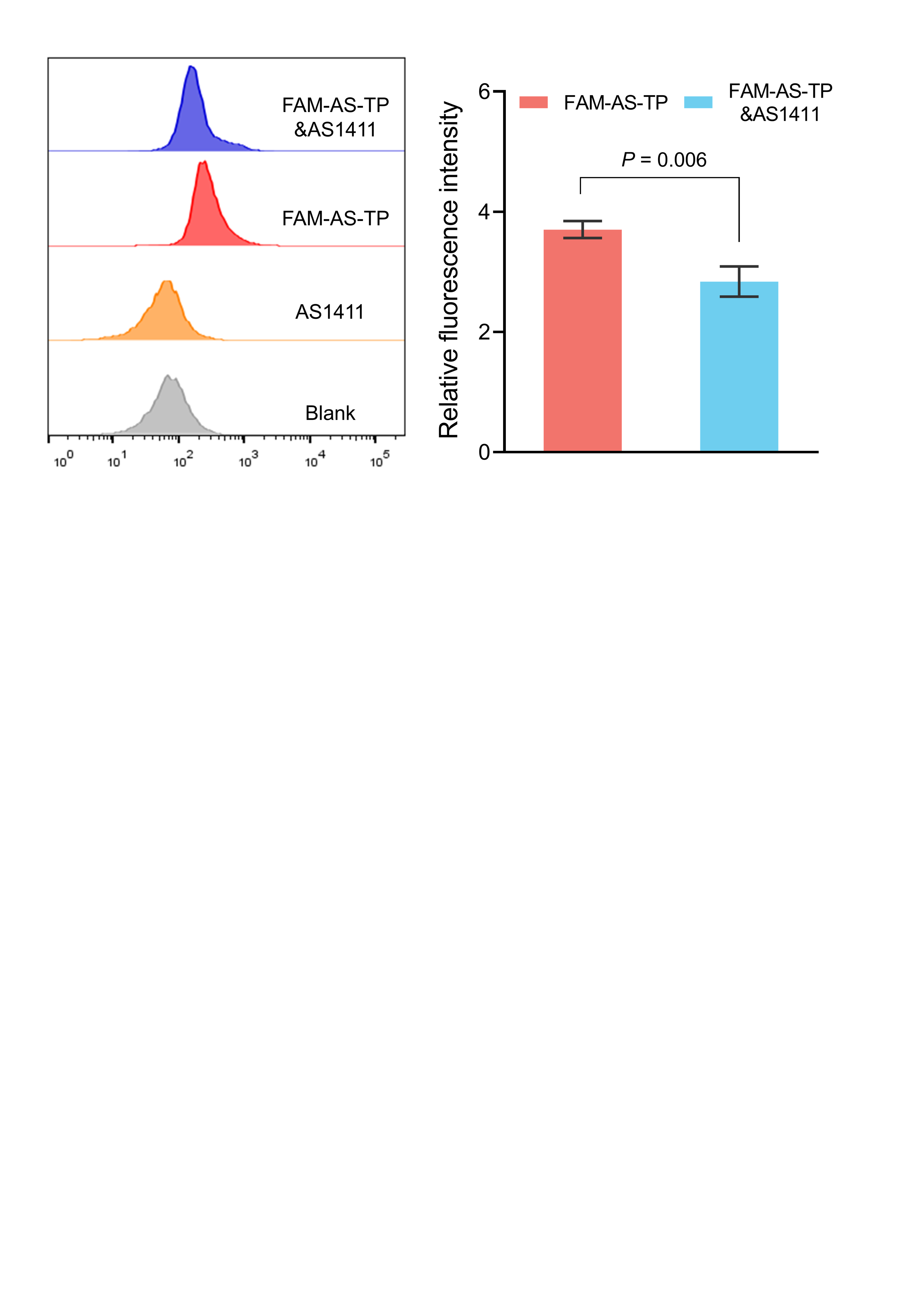


**Figure S33.** AS1411 competitively inhibited AS-TP uptake by MDA-MB-231 cells.


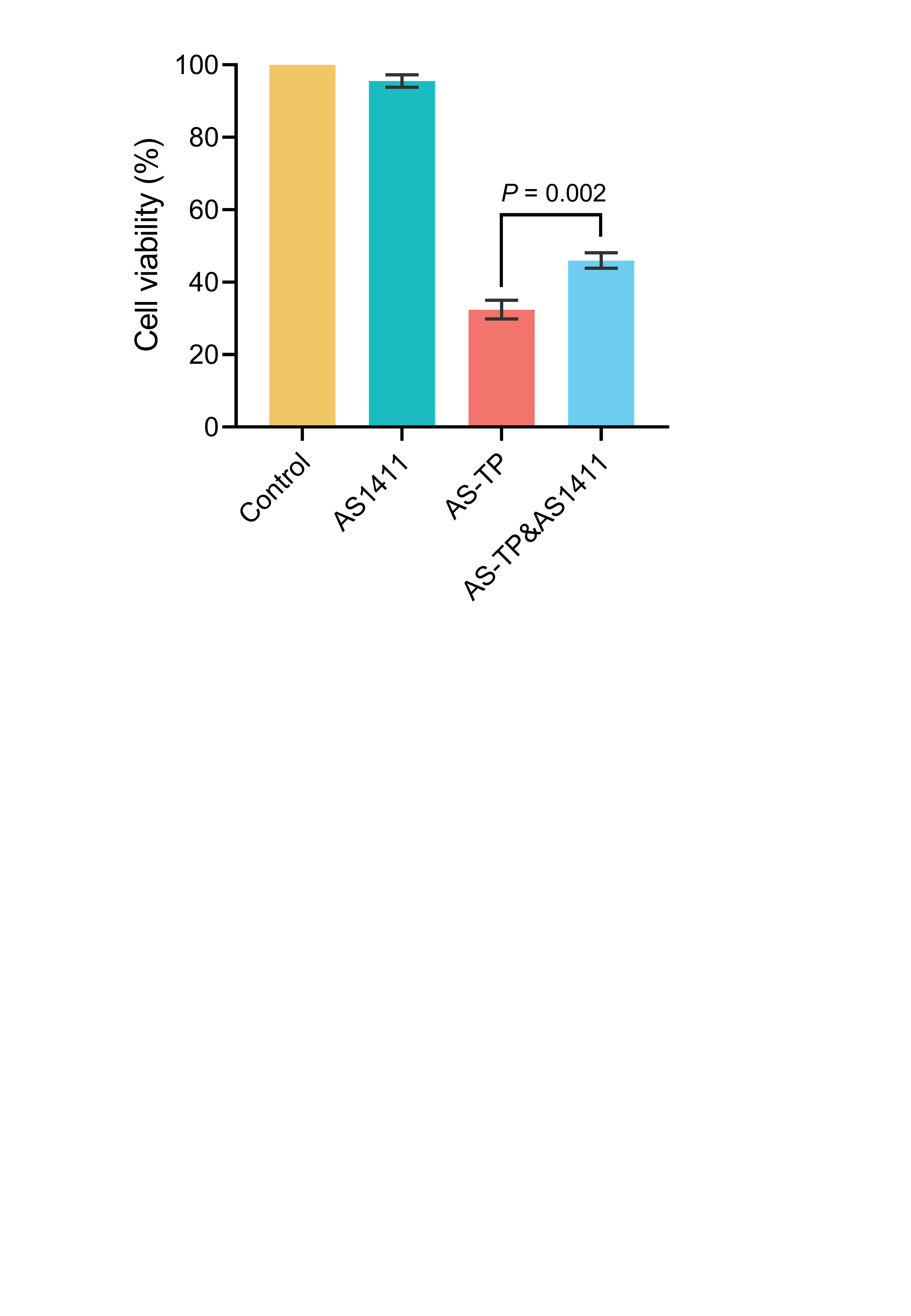


**Figure S34.** Co-cultured AS1411 attenuated the cytotoxic effect of AS-TP on MDA-MB-231 cells.


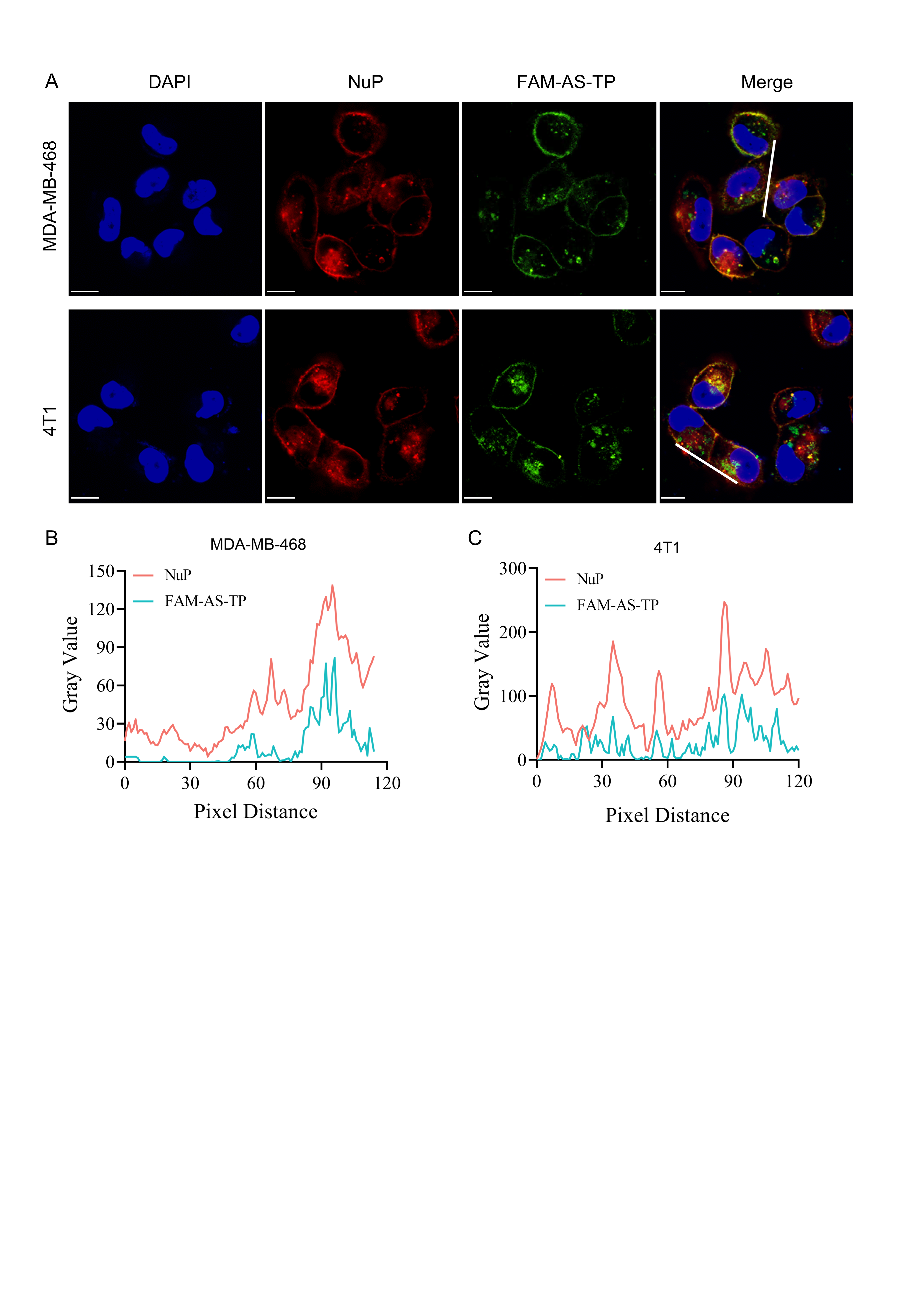


**Figure S35.** (A) Co-localization of FAM-AS-TP with NuP on the surface of MDA-MB-468 and 4T1 cell membrane. (NuP: red; FAM-labeled drugs: green; cell nucleus: blue). Bar = 10 μm. Magnification: 600X. (B-C) Quantification of co-localization in was analyzed by Image J in MDA-MB-468 cells (B) and 4T1 cells (C) .


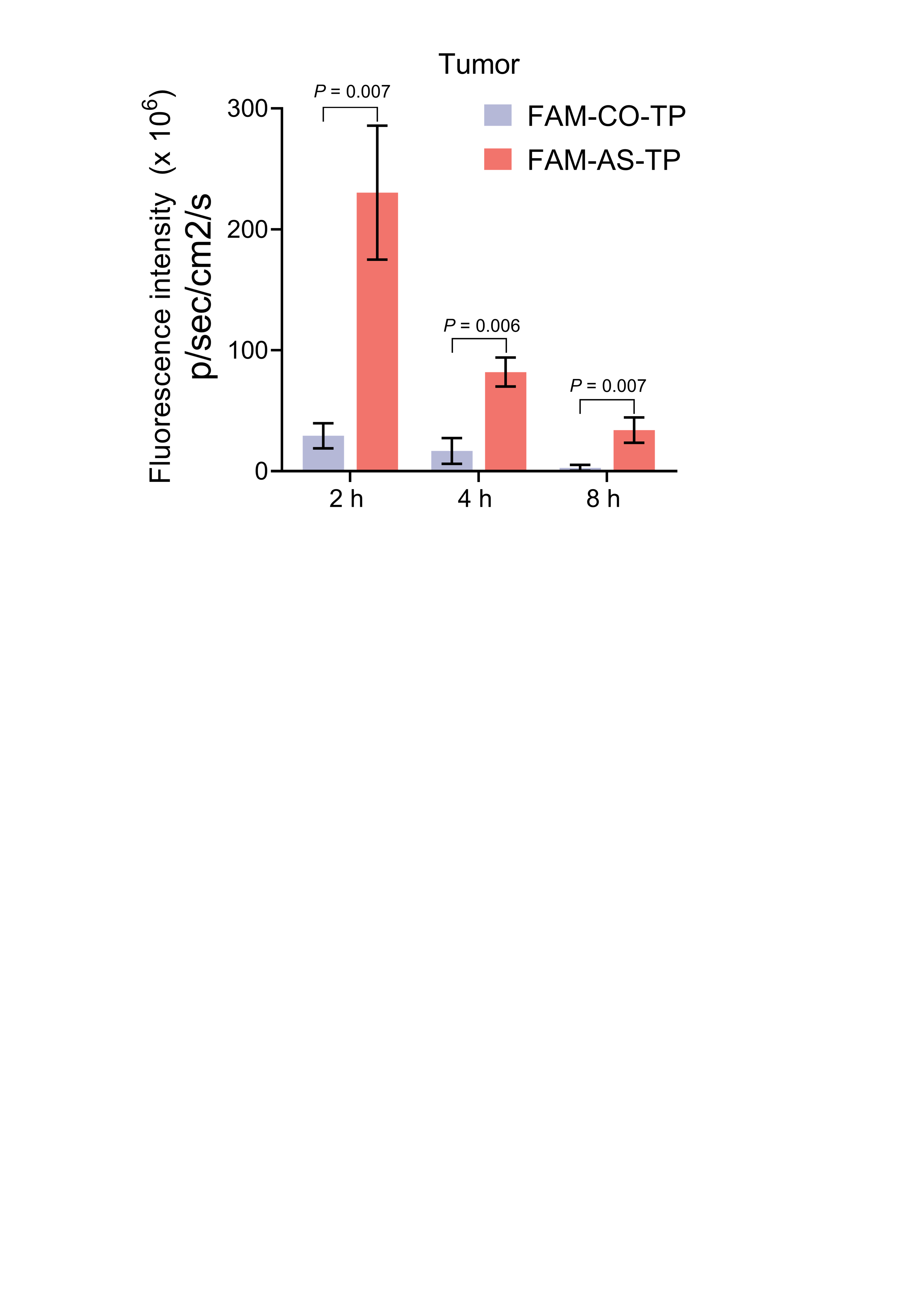


**Figure S36.** Semiquantitative analysis of fluorescence intensity in tumor tissues *in vivo* at different administration times.


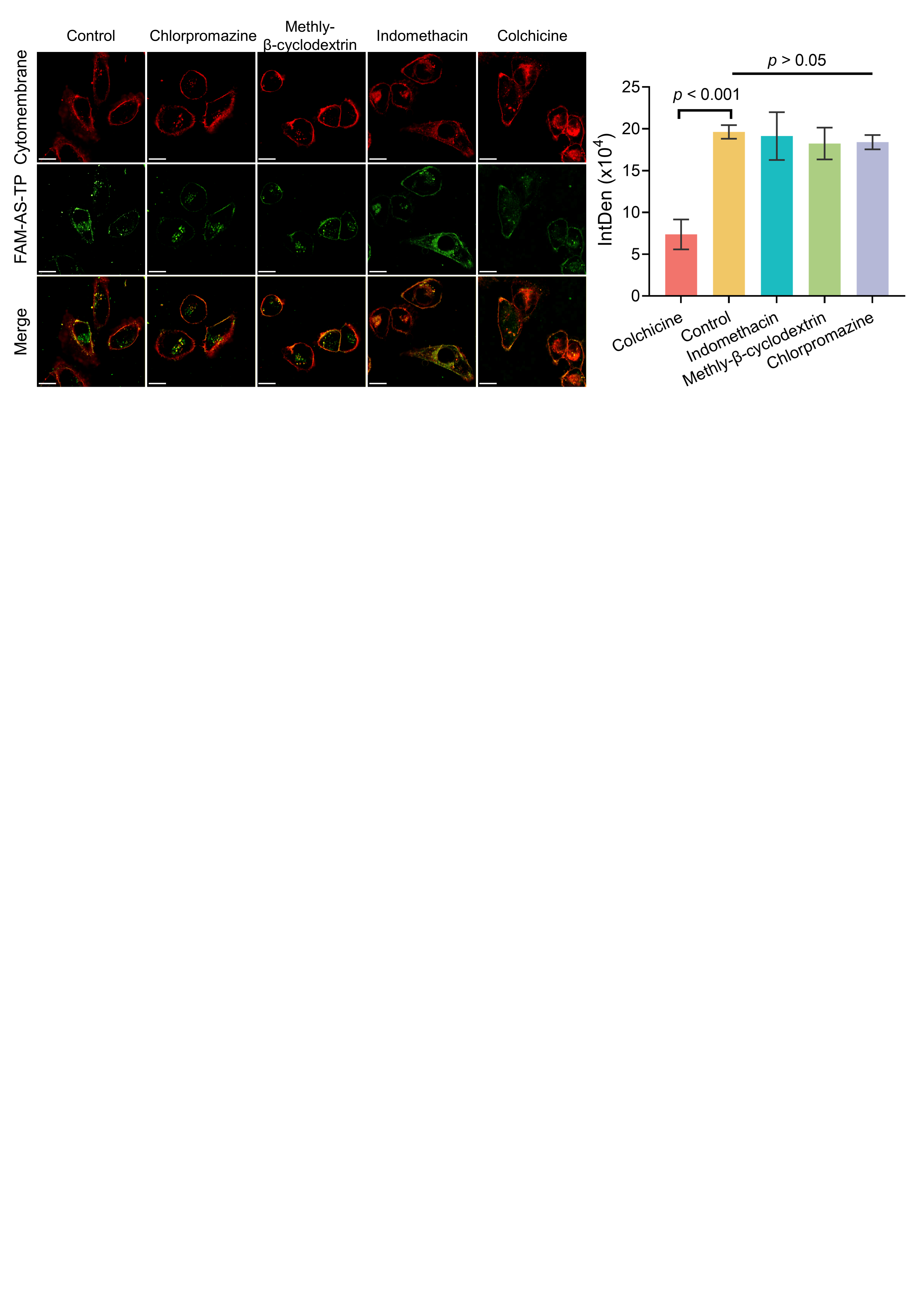


**Figure S**37. The laser confocal microscopy determination of the endocytosis pathways for FAM-AS-TP. The fluorescent intensity of the uptaken FAM-AS-TP in MDA-MB-231 cells after pre-incubation with different endocytic pathway inhibitors for 2 h. (Membrane: red; FAM-AS-TP: green). Bar = 10 μm. Magnification: 600X.


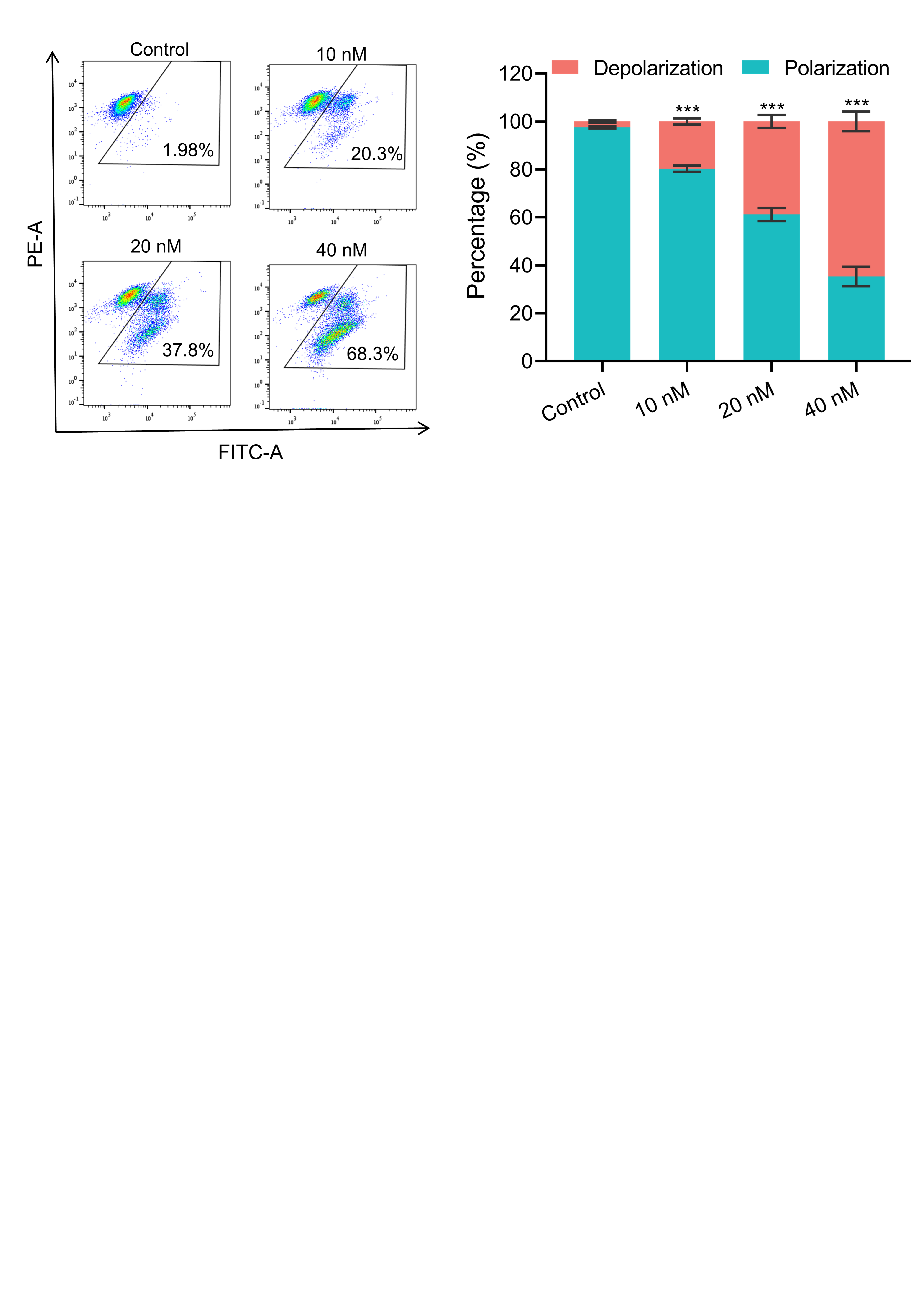


**Figure S**38. The variation of mitochondrial membrane potential after the treatment of AS-TP with different concentrations on MDA-MB-231 cells. Compared to the control group, **p* < 0.05, ***p* < 0.01, ****p* < 0.001, ns *p* > 0.05.


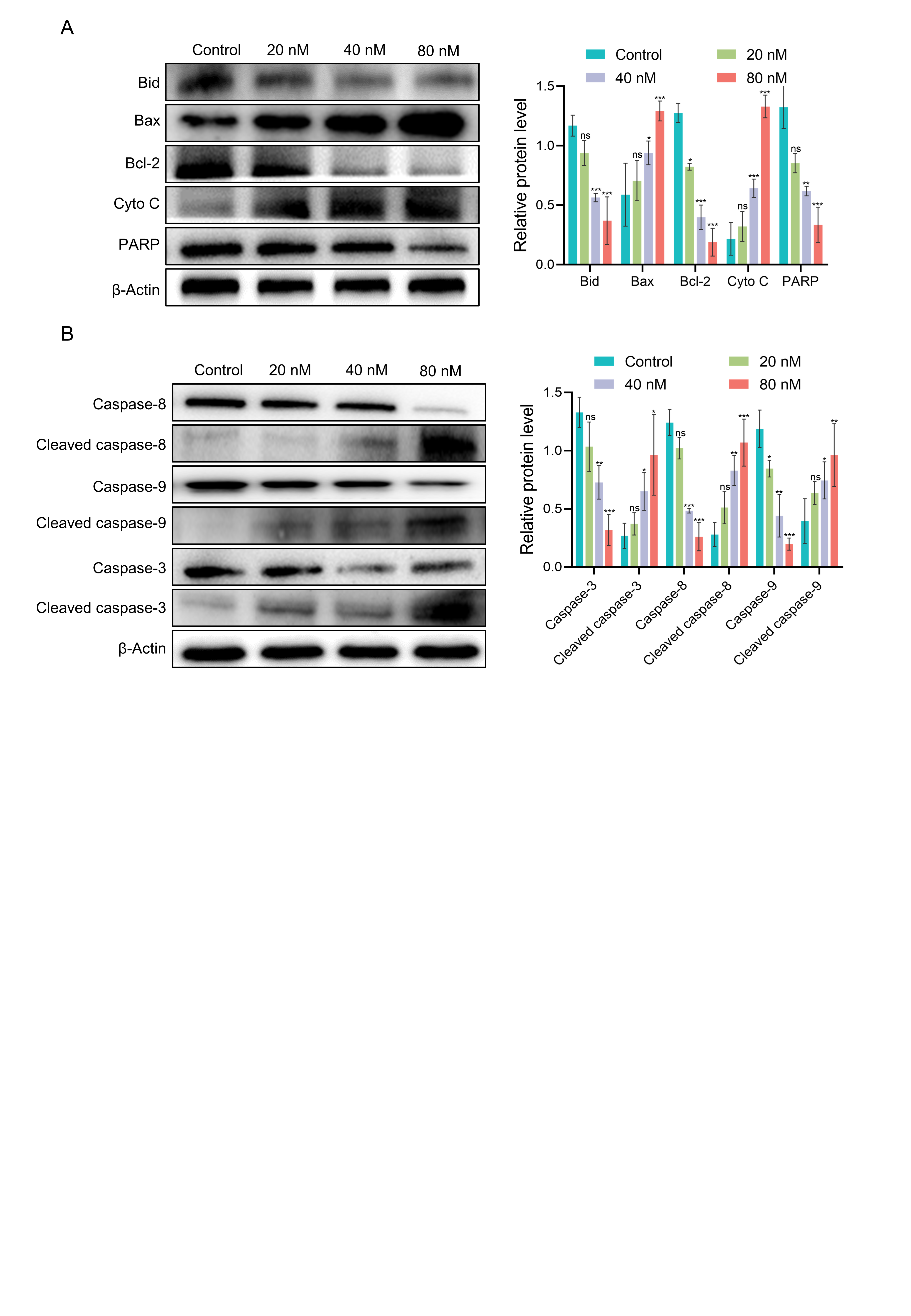


**Figure S**39. The effects of AS-TP on the mitochondrial apoptotic pathway in MDA-MB-231 cells. A) The changes of Bid, Bax, Bcl-2, Cyto C and PARP protein levels after AS-TP administration on MDA-MB-231 cells. B) The changes of caspase-3/8/9 and cleaved caspase-3/8/9 protein levels after AS-TP administration on MDA-MB-231 cells. All protein levels were quantified by standardizing β-Actin. Compared to the control group, **p* < 0.05, ***p* < 0.01, ****p* < 0.001, ns *p* > 0.05.

Table S1. Sequence data for DNA

| **DNA** | **Sequence** |
| --- | --- |
| AS1411 | 5’CH_2_CH_2_-(OCH_2_CH_2_)3-O-GGTGGTGGTGGTTGTGGTGGTGGTGG3’ |
| CRO | 5’CH_2_CH_2_-(OCH_2_CH_2_)_3_-O-CCTCCTCCTCCTTCTCCTCCTCC3’ |
| Amino-AS1411 | 5’ amino-CH_2_CH_2_-(OCH_2_CH_2_)_3_-O-GGTGGTGGTGGTTGTGGTGGTGGTGG3’ |
| Amino-CRO | 5’ amino-CH_2_CH_2_-(OCH2CH2)_3_-O-CCTCCTCCTCCTTCTCCTCCTCC3’ |
| Amino-AS1411-FAM | 5’ amino-CH_2_CH_2_-(OCH_2_CH_2_)_3_-O-GGTGGTGGTGGTTGTGGTGGTGGTGG-FAM 3’ |
| Amino-CRO-FAM | 5’ amino-CH_2_CH_2_-(OCH_2_CH_2_)_3_-O-CCTCCTCCTCCTTCTCCTCCTCC-FAM 3’ |
| TP-amino-AS1411 | 5’ TP-amino-CH_2_CH_2_-(OCH_2_CH_2_)_3_-O-GGTGGTGGTGGTTGTGGTGGTGGTGG3’ |
| TP-amino-CRO | 5’ TP-amino-CH_2_CH_2_-(OCH_2_CH_2_)_3_-O-CCTCCTCCTCCTTCTCCTCCTCC3’ |
| TP-amino-AS1411-FAM | 5’ TP-amino-CH_2_CH_2_-(OCH_2_CH_2_)_3_-O-GGTGGTGGTGGTTGTGGTGGTGGTGG-FAM 3’ |
| TP-amino-CRO-FAM | 5’ TP-amino-CH_2_CH_2_-(OCH_2_CH_2_)_3_-O-CCTCCTCCTCCTTCTCCTCCTCC-FAM 3’ |

**Table S2.** The pharmacokinetic parameters of AS-TP in mice. AUC: area under curve; Vd: apparent volume of distribution; T_1/2α_: distribution half-life time; T_1/2β_: elimination half-life time; CL: elimination clearance; Ke: elimination rate constant; K_12_: rate constants for AS-TP distribution from the central compartment to the peripheral compartment; K_21_: rate constants of AS-TP from the peripheral to the central compartment distribution rate constant.

| Parameter | Units | Value |
| --- | --- | --- |
| AUC | mg/L*h | 84.87 ± 13.57 |
| Vd | L/kg | 0.54 ± 0.24 |
| T_1/2α_ | h | 0.55 ± 0.23 |
| T_1/2β_ | h | 7.32 ± 0.90 |
| CL | L/h/kg | 0.17 ± 0.08 |
| Ke | 1/h | 0.31 ± 0.02 |
| K_12_ | 1/h | 0.83 ± 0.37 |
| K_21_ | 1/h | 0.76 ± 0.35 |
| C_max_ | h | 26.32 ± 1.87 |

Table S3. Blood routine report of mice.

| Projects | Units | Control | Elibulin | nab-PTX | AS-TP |
| --- | --- | --- | --- | --- | --- |
| WBC | 10^9/L | 4.97 ± 4.39 | 1.6 ± 2.03 | 3.27 ± 1.44 | 3.33 ± 1.21 |
| Neu# | 10^9/L | 1.66 ± 1.19 | 0.0 ± 0.03 * | 0.26 ± 0.31 | 1.70 ± 0.81 |
| Lym# | 10^9/L | 2.95 ± 2.38 | 1.5 ± 2.02 | 2.69 ± 1.27 | 1.63 ± 0.69 |
| Mon# | 10^9/L | 0.1 ± 0.00 | 0.1 ± 0.00 | 0.01 ± 0.00 | 0.03 ± 0.00 |
| Eos# | 10^9/L | 0.01 ± 0.00 | 0.1 ± 0.00 | 0.01 ± 0.00 | 0.01 ± 0.00 |
| Neu% | % | 41.33 ± 27.22 | 5.8 ± 0.88 * | 6.83 ± 5.85 * | 46.70 ± 9.15 |
| Lym% | % | 58.30 ± 26.97 | 93. ± 0.41 * | 92.63 ± 5.84 * | 53.15 ± 9.23 |
| Mon% | % | 0.40 ± 0.00 | 0.8 ± 0.57 | 0.55 ± 0.26 | 0.35 ± 0.21 |
| Eos% | % | 0.40 ± 0.52 | 0.4 ± 0.26 | 0.30 ± 0.00 | 0.15 ± 0.07 |
| RBC | 10^12/L | 10.51 ± 0.74 | 8.4 ± 0.94 | 8.96 ± 0.79 | 8.84 ± 0.84 |
| HGB | g/L | 161.00 ± 9.02 | 128 ± 13.05 | 136.25 ± 11.32 | 134.75 ± 14.80 |
| HCT | % | 48.13 ± 2.35 | 38. ± 3.90 | 41.28 ± 3.10 | 40.98 ± 4.34 |
| MCV | fL | 45.98 ± 1.09 | 46. ± 0.71 | 45.90 ± 0.98 | 46.93 ± 0.77 |
| MCH | pg | 15.33 ± 0.22 | 15. ± 0.20 | 15.18 ± 0.05 | 15.35 ± 0.24 |
| MCHC | g/L | 334.50 ± 2.38 | 331 ± 1.26 | 329.50 ± 3.87 | 329.25 ± 1.89 |
| RDW-CV | % | 14.48 ± 0.42 | 15. ± 0.40 | 14.78 ± 0.29 | 15.10 ± 0.14 |
| RDW-SD | fL | 26.80 ± 1.07 | 29. ± 0.63 | 27.50 ± 0.80 | 28.40 ± 0.43 |
| PLT | 10^9/L | 279.75 ± 239.79 | 46. ± 15.03 * | 155.25 ± 125.86 | 266.75 ± 208.73 |
| MPV | fL | 6.90 ± 0.41 | 7.3 ± 0.15 | 6.65 ± 0.26 | 6.40 ± 0.27 |
| PDW | *** | 15.90 ± 0.75 | 14. ± 0.10 | 15.55 ± 0.19 | 15.43 ± 0.38 |
| PCT | % | 0.20 ± 0.19 | 0.0 ± 0.01 | 0.10 ± 0.08 | 0.17 ± 0.13 |
| P-LCC | 10^9/L | 28.00 ± 19.80 | 18. ± 17.68 | 14.50 ± 15.20 | 45.00 ± 33.94 |
| P-LCR | % | 15.40 ± 2.26 | 9.8 ± 0.28 | 9.78 ± 1.98 * | 8.80 ± 2.83 |

**Experimental Section**

***Materials and reagents:*** All the starting chemical materials were obtained from commercial sources (Shanghai Aladdin Biochemical Co., Ltd, Chengdu Pukang Biological Technology Co., Ltd, and Shanghai Macklin Biochemical Co., Ltd) and used without further purification unless otherwise stated. The consumables (Sodium chloride, anhydrous sodium sulfate, etc.) and solvents (Dichloromethane, methanol, ethyl acetate, petroleum ether, etc.) were obtained from Jinshan Chemical Test (Chengdu, China). The progress of the reactions was monitored by thin layer chromatography (TLC) or liquid chromatography-mass spectrometry (Thermo Fisher Scientific, USA), and flash column chromatography was performed using silica gel (200 - 300 mesh). Conjugate reactions were monitored by high-performance liquid chromatography (Shimadzu, Japan), and purified by reversed-phase high-performance liquid chromatography (Shimadzu, Japan). ^1^H NMR and ^13^C NMR spectra were recorded on Bruker-400 MHz spectrometer, Bruker-600 MHz spectrometer or Bruker-700 MHz spectrometer (BRUKER, Germany). Chemical shifts (δ) are given in parts per million (ppm), and the residual solvent peaks were used as an internal reference (CDCl_3_: δ 7.26 ppm ^1^H; δ 77.2 ppm ^13^C; *d*_4_-MeOD: δ 3.31 ppm ^1^H; δ 49.00 ppm ^13^C; *d_6_*-DMSO: δ 2.50 ppm ^1^H; δ 39.5 ppm ^13^C). Accurate mass measurements were performed on an electrospray ionization (ESI) apparatus using Agilent 1260-Bruker tims TOF mass. Recombinant Human Nucleolin (pro-1508) was obtained from ProSpec (Ness Ziona, Israel); CM5 chip (29104988) and Amino Coupling Kit (BR-1000-50) were from Cytvia (Marlborough, MA); Alexa Fluor 555-labeled donkey anti-mouse IgG (A0460), Annexin V-FITC Apoptosis Detection Kit (C1062L-2), Cell Cycle and Apoptosis Analysis Kit (C1052-1), Mitochondrial membrane potential assay kit with JC-1 (C2006-2), One Step TUNEL Apoptosis Assay Kit (C1086) were purchased Beyotime (Shanghai, China); C23 (MS-3) (sc-8031), from Santa Cruz Biotechnology (Santa Cruz, CA); CytoMBriteTM (D4019), DAPI (D4054) were provided by US EVERBRIGHT® Inc. (Suzhou, China); Lyso-Tracker Red (L8010), Oxygen Species Assay Kit (CA1410) were supplied by Solarbio (Beijing, China); QuantiCyto Mouse IL-6 ELISA kit (High Sensitivity) (EMC004 (H).96.10), QuantiCyto Mouse TNF-α ELISA kit (High Sensitivity) (EMC102a (H).96.10 were purchased from NEOBOSCIENCE (Shenzhen, China).

***Synthesis of compound 1:*** A mixture of triptolide (1.00 g, 2.78 mmol) and AcOH (1.33 g, 22.23 mmol) in DMSO (15.00 mL) was stirred at 0 ℃, then Ac_2_O was added (0.57 g, 5.56 mmol) in 40 minutes, which was stirred at 0 ℃ for 1 hour, then warmed to room temperature and stirred for 24 hours, monitored by TLC until triptolide was consumed. The mixture was diluted with DCM (50.00 mL). The organic layer was washed with water (20.00 mL × 3) and brine (20.00 mL), dried with anhydrous sodium sulfate and evaporated under reduced pressure. The residue was purified by flash chromatography on silica gel (PE : EA = 1 : 1) to afford compound **1** as a yellow solid (1.02 g, 87.4% yield).

***Synthesis of compound 2:*** A mixture of succinic anhydride (1.00 g, 10.00 mmol) and DMAP (1.46 g, 12.00 mmol) in dry toluene (10.00 mL) was stirred at room temperature, then allyl alcohol (0.29 g, 5.00 mmol) was added. The mixture was stirred at 40 ℃ in an oil bath and monitored by LC-MS until allyl alcohol was consumed. Concentrated under reduced pressure and then diluted with EA (50.00 mL). The organic layer was washed with water (20.00 mL × 3) and brine (20.00 mL), dried with anhydrous sodium sulfate, evaporated under reduced pressure, and the residue was purified by flash chromatography on silica gel (PE : EA = 1 : 1) to afford compound **2** as a white solid (1.40 g, 88.6% yield).

***Synthesis of compound 3:*** NaHCO_3_ (0.19 g, 2.30 mmol) was added to a solution of compound **2** (0.70 g, 4.43 mmol) in acetone (15.00 mL). The solution was stirred at room temperature for 1 hour, monitored by TLC, filtered, and concentrated in vacuo to give compound **3** (0.73 g, 92.1% yield) as a white solid which was employed without further purification in the next step.

***Synthesis of compound 4:*** To a solution of compound **1** (1.00 g, 2.38 mmol) in dry DCM (20.00 mL) was added dropwise, under argon, a 1.00 M sulfuryl chloride solution in DCM (2.40 mL, 2.40 mmol). The reaction mixture was stirred at room temperature for 2 h. After completion of the reaction, the chloromethyl ether derivative was obtained as a yellow foam after evaporation of the solvent and was directly used in the next step. A solution of the chloromethyl ether derivative in dry DCM (20.00 mL) was added dropwise to a solution of compound **3** (0.74 g, 4.09 mmol) and 15-crown-5 (0.48 g, 1.79 mmol) in dry DCM (20.00 mL). After stirring at room temperature for 1 hour. Then the organic layer was washed with water (20.00 mL × 3) and brine (20.00 mL), dried with anhydrous sodium sulfate, evaporated under reduced pressure, and the residue was purified by flash chromatography on silica gel (PE : EA = 2 : 1) to afford compound **4** as a yellow solid (0.97 g, 77.4% yield).

***Synthesis of compound 5:*** To a solution of compound **4** (0.97 g, 1.84 mmol) in dry THF (20.00 mL) was added morpholine (1.60 g, 18.40 mmol) and Pd(PPh_3_)_4_ (0.21 g, 0.18 mmol). The mixture was stirred in the dark at 25 ℃ for 2 h. The organic layer was washed with water (20.00 mL × 3), dried with anhydrous sodium sulfate, and evaporated under reduced pressure. The residue was purified by flash chromatography on silica gel (DCM : MeOH = 15 : 1) to afford compound **5** as a yellow solid (0.48 g, 54.5% yield).

***Synthesis of AS-TP:*** Sulfo-NHS (7.80 mg, 36.00 μmol) dissolved in dd-H_2_O (100.00 μL) was added to the solution of compound **5** (14.70 mg, 30.00 μmol) and EDCI (7.50 mg, 390.0 μmol) in DMF (300.00 μL). The mixture was stirred at 37 ℃ for 2 h. Activated compound **5** was then incubated with amino-modified nucleolin aptamer. Nucleolin aptamer (1.00 mg, 0.12 μmol) was dissolved in 0.5 M Na_2_CO_3_/NaHCO_3_ buffer (100.00 μL, pH 8.4) in a 2.00 mL centrifuge tube (20.00 tubes total), and 200.00 μL freshly prepared compound **5** N-hydroxysulfosuccinimide ester reaction solution was added. After 2 h, an additional 200.00 μL of the active ester reaction solution was added (400.00 μL, 30.00 μmol total). And then, the reaction solution was mixed at 37 ℃ overnight, monitored by HPLC until nucleolin aptamer was consumed, then it was centrifugated and the residue was purified by RP-HPLC. The desired fraction was collected and lyophilized to give AS-TP (18.00 mg, 85.3% yield) as a white solid.

*CO-TP, FAM-AS-TP and FAM-CO-TP were synthesized according to the above procedure.*

***Synthesis of compound 8:*** To a solution of propiolic acid (0.91 g, 13.00 mmol) in DMF (10.00 mL) was added NaHCO_3_ (2.18 g, 26.00 mmol) portion-wise. After 1 hour of stirring at 25 ℃, allyl bromide (2.28 g, 16.90 mmol) was added, and the reaction was stirred at room temperature overnight, monitored by LC-MS until propiolic acid was consumed. The reaction was diluted with EA (50.00 mL) and washed with water (20.00 mL × 3). The organic layer was dried over Na_2_SO_4_, filtered, and concentrated in vacuo, then the residue was purified by flash chromatography on silica gel (PE : EA = 1 : 1) to afford compound **8** (1.30 g, 90.8% yield) as a yellow oil.

***Synthesis of compound 9:*** To a solution of triptolide (50.00 mg, 139.00 μmol) and compound **8** (23.00 mg, 208.00 μmol) in ACN (10.00 mL) was added NMM (15.00 μL, 69.50 μmol). The mixture was stirred at room temperature for 12 hours and monitored by LC-MS until triptolide was consumed. Then the solution was concentrated and the residue was purified by flash chromatography on silica gel (PE : EA = 2 : 1) to afford compound **9** (33.00 mg, 51.1% yield) as a white solid.

***Synthesis of compound 10:*** To a solution of compound **9** (33.00 mg, 70.20 μmol) in THF (10.00 mL) was added morpholine (50.00 μL, 0.71 mmol) and Pd (PPh_3_)_4_ (14.00 mg, 10.50 μmol) at room temperature. After the consumption of compound **9** (monitored by LC-MS), the mixture was evaporated to give a crude compound, and the residue was purified by flash chromatography on silica gel (DCM : MeOH = 15 : 1) to give compound **10** (10.00 mg, 32.8% yield) as a white solid.

***Synthesis of AS-TP(V):*** Sulfo-NHS (13.00 mg, 58.00 μmol) dissolved in dd-H_2_O (100.00 μL) was added to the solution of compound **10** (21.50 mg, 50.00 μmol) and EDCI (12.50 mg, 651.0 μmol) in DMF (300.00 μL). The mixture was stirred at 37 ℃ for 2 hours. Activated compound **9** was then incubated with amino-modified nucleolin aptamer. Nucleolin aptamer (1.68 mg, 200.00 nmol) was dissolved in 0.5 M Na_2_CO_3_/NaHCO_3_ buffer (100.00 μL, pH 8.4) in a 2.00 mL centrifuge tube, and 200.00 μL freshly prepared compound **10** N-hydroxysulfosuccinimide ester reaction solution was added. After 2 hours, an additional 200.00 μL of the active ester reaction solution was added (400.00 μL, 50.00 μmol total), and then, the reaction solution was mixed at 37 ℃ overnight, monitored by HPLC until nucleolin aptamer was consumed, then it was centrifugated and the residue was purified by RP-HPLC. The desired fraction was collected and lyophilized to give AS-TP(V) (1.58 mg, 88.7% yield) as a white solid.

***Synthesis of compound 11:*** To a solution of Fmoc-valine (1.00 g, 2.94 mmol) in DCM (50.00 mL) was added NHS (0.51 g, 4.41 mmol) and EDCI (0.85 g, 4.41 mmol). The mixture was stirred at 25 ℃ for 8 hours, then the organic layer was washed with water (20.00 mL × 3), dried with anhydrous sodium sulfate, and evaporated under reduced pressure. The residue was purified by flash chromatography on silica gel (PE : EA = 1 : 1) to afford compound **11** as a white solid (1.26 g, 94.5% yield).

***Synthesis of compound 12:*** To a solution of compound **11** (1.20 g, 2.75 mmol) in THF (50.00 mL) was added L-citrulline (0.56 g, 3.30 mmol), then a solution of NaHCO_3_ (0.35 g, 4.12 mmol) in H_2_O (50.00 mL) was added. The mixture was stirred at 50 ℃ in an oil bath and monitored by TLC until compound **11** was consumed, then hydrochloric acid was added. The organic layer was washed with water (20.00 mL × 3), dried with anhydrous sodium sulfate, and evaporated under reduced pressure. The residue was purified by flash chromatography on silica gel (PE : EA = 1 : 1) to afford compound **12** as a white solid (1.16 g, 85.4% yield).

***Synthesis of compound 13:*** To a solution of compound **12** (1.10 g, 2.22 mmol) in dry DCM (50.00 mL) was added 4-aminobenzyl alcohol (0.55 g, 4.44 mmol) and EEDQ (1.10 g, 4.44 mmol). The mixture was stirred in the dark at room temperature until compound **12** was consumed. The organic layer was washed with water (20.00 mL × 3) and brine (20.00 mL), dried with anhydrous sodium sulfate, and evaporated under reduced pressure. The residue was purified by flash chromatography on silica gel (DCM : MeOH = 15 : 1) to afford compound **13** as a white solid (1.02 g, 76.5% yield).

***Synthesis of compound 14:*** To a solution of compound **13** (1.00 g, 1.66 mmol) in dry DMF (20.00 mL) was added piperidine (471.00 μL, 5.81 mmol). Then the mixture was stirred at room temperature for 1 hour, monitored by TLC until compound **13** was consumed, and then diluted with DCM (50.00 mL). The organic layer was washed with water (20.00 mL × 3) and brine (20.00 mL), dried with anhydrous sodium sulfate, and evaporated under reduced pressure. The residue was purified by flash chromatography on silica gel (DCM : MeOH = 3 : 1) to afford compound **14** as a white solid (0.53 g, 84.2% yield).

***Synthesis of compound 15:*** To a solution of compound **14** (100.00 mg, 0.26 mmol) in DMSO (20.00 mL) was added triptolide derivatives (137.20 mg, 0.28 mmol) and HATU (127.68 mg, 0.34 mmol) / DIPEA (73.00 μL, 0.42 mmol). The mixture was stirred at room temperature for 6 hours, and monitored by TLC until compound **14** was consumed. The organic layer was washed with water (20.00 mL × 5), dried with anhydrous sodium sulfate, and evaporated under reduced pressure. The residue was purified by flash chromatography on silica gel (DCM : MeOH = 5 : 1) to afford compound **15** as a yellow solid (179.88 mg, 81.3% yield).

***Data Collection:*** The unified and standardized pan-cancer dataset, TCGA TARGET GTEx, was downloaded from the UCSC database (https://xenabrowser.net/). We specifically extracted the expression data of the ENSG00000115053 (NCL) gene from breast cancer samples. Subsequently, a log2(x+0.001) transformation was applied to each expression value. Finally, cancer types with less than 3 samples were excluded from the analysis.

***Molecular modeling:*** The three-dimensional prediction models of AS1411 and AS-TP were generated by docking calculation based on a G-quadruplex structure (PDB ID: 2N3M). The structure of the RNA binding domain (RBD) of nucleolin was downloaded from the RCSB PDB database (PDB ID: 1RKJ). The molecular dynamic for the coarse structures was implemented for energy minimization and optimization in the amber force field^1^. Molecular docking was performed to generate the initial complex of AS1411 (or AS-TP) and nucleolin by using HADDOCK 2.4^2^. The binding free energy was calculated with the MM-PBSA algorithm.

***Surface plasmon resonance (SPR)******:*** The Biacore X100 system (Biacore T200, GE Healthcare) was employed to detect the affinity and interaction between nucleolin and drugs *in vitro*. CM5 sensor chips were immobilized with His-tagged nucleolin proteins via the amino-coupling method at pH = 4.5, resulting in immobilization levels of 1000-1500 RU. Different drugs (AS-TP, CO-TP, AS1411, and TP) in selected concentrations were passed across the chip surface at a flow rate of 30 µL/min, and the binding between nucleolin and drugs was recorded in real time. The CM5 chip surface was regenerated with 50 mM NaOH after each run. Kinetic parameters were obtained by kinetic curve fitting using the BIA evaluation software.

***Cell culture:*** MDA-MB-231 (human triple-negative breast cancer cells), MDA-MB-468(human triple-negative breast cancer cells), 4T1(mouse triple negative breast cancer cells), MCF-7 (human breast cancer cells), and MCF-10A (non-malignant breast epithelial cells) were purchased from American Type Culture Collection or The Cell Bank of Type Culture Collection of Chinese Academy of Sciences (Shanghai, China). All cell culture procedures were performed in standard conditions specified by the manufacturer.

***Cellular immunofluorescence assay:*** The expression of nucleolin in different cells was assessed by an immunofluorescence experiment. MDA-MB-231, MDA-MB-468, MCF-10A, MCF-7 and 4T1 cells were seeded in a 12-well plate with sterile glass slides or in 6-well plates. After adherence, cells were incubated with the primary anti-C23 antibody for 1 h in a 5% CO_2_ incubator. Subsequently, cells were incubated with Alexa Fluor 555-labeled donkey anti-mouse IgG and fixed. Fluorescence intensity was detected by flow cytometer (BD FACSVerse, BD Biosciences) and confocal laser microscopy (FV-OSR, Olympus).

***Uptake assay:*** After overnight incubation, MDA-MB-231, MDA-MB-468,4T1, MCF-10A, and MCF-7 cells were treated with FAM-AS-TP or FAM-CO-TP at a final concentration of 400 nM for 2 h. The treated cells were subsequently collected on a FACSVevse flow cytometer and analyzed using the FlowJo software.

MDA-MB-231 cells were allowed to seed onto 6-well plates and adhere overnight, then add 1 mL medium containing 400 nmol/L FAM-labeled AS1411-TP and incubate at 37 ℃ for different times (2 h, 4 h, 8 h, 16 h, and 24 h). Additionally, for the effect of concentrations on the uptake, solutions with different FAM-AS-TP concentrations (200 nM, 400 nM, 600 nM, and 800 nM) were employed to incubate with MDA-MB-231 cells 4 h. Cells were run on a FACSVerse flow cytometer and then analyzed by FlowJo software. To further investigate the effect of time on cellular uptake, we observed the cell uptake of FAM-AS-TP under a confocal laser scanning microscope (CLSM) at different times.

***Imaging colocalization experiment:*** Colocalization of nucleolin and FAM-label drugs was assessed by confocal imaging experiments. Firmly adherent MDA-MB-231 cells were allowed to incubate with FAM-labeled AS-TP, AS1411, or CO-TP for 1 h. MDA-MB-468 and 4T1 cells were pretreated with FAM-AS-TP only. Subsequent steps of immunofluorescence microscopy were described above. To determine the colocalization of lysosomal and FAM-label drugs, we carried out confocal fluorescence imaging experiments. MDA-MB-231 cells were infected with FAM-label AS-TP for 3 h. Cells were washed with PBS buffer, and then incubated with Lyso-Tracker Red working solution for 1 h at 37 ℃. After being fixed with 4% paraformaldehyde, cells were counterstained with DAPI staining and observed with a fluorescence microscope.

***Competitive binding experiment:*** Competitive inhibition tests were conducted to evaluate the selectivity of AS-TP. Specifically, MDA-MB-231 cells were pre-incubated with AS1411 prior to treatment with AS-TP for a specified duration. Subsequently, samples were collected for flow cytometry analysis to quantify the fluorescence intensity of cells that internalized FAM. Furthermore, the MTT method was employed to assess the cytotoxicity of AS-TP on cells in the presence of AS1411 interference.

***Endocytosis pathway study:*** To elucidate the endocytic pathway of AS-TP, we employed different endocytic inhibitors to inhibit specific pathways and measured the uptake of FAM-AS-TP in MDA-MB-231 cells by flow cytometry and confocal microscopy (Colchicine: a micropinocytosis pathway inhibitor, 10 nM; chlorpromazine: clathrin-mediated endocytosis inhibitor, 5 μM; indomethacin: caveolin-mediated endocytosis inhibitor, 10 μM; methyl-β-cyclodextrin: cholesterol-mediated endocytosis inhibitor, 10 μΜ). Briefly, MDA-MB-231 cells were incubated in the presence of different endocytic inhibitors for 2 h, and then FAM-AS-TP (200 nM) was added and cells were treated for 4 h. Subsequently, the fluorescent intensity was analyzed by flow cytometry (FITC channel). For imaging experiments, after drug incubation, cells were visualized using the DID (membrane red fluorescent dye), whereas the nucleus was identified by DAPI staining. Confocal laser scanning microscopy was performed to estimate the uptake of FAM-AS-TP.

***Cytotoxicity assay:*** The cell viability of MDA-MB-231,MDA-MB-468, MCF-7, MCF-10A and 4T1 cells in different drugs was evaluated by MTT assay. Briefly, cells were cultured on 96-well plates and exposed to different concentrations of AS-TP, and CO-TP for 48 h. After treatment, cells were incubated with 10 μL MTT solution for 2 h at 37 ℃. MTT formazan was dissolved in 100 μL DMSO and absorbance was measured immediately at 490 nm in the microplate reader (Thermo Fisher Scientific).

***Cell apoptosis assay:*** After treatment with 40 nM of TP, AS-TP and CO-TP , detection of apoptosis was performed by an Annexin V-FITC Apoptosis Detection Kit. MDA-MB-231 cells were seeded into 6-well plates and cultured with AS-TP, CO-TP or TP for 48 h. Then cells were collected and resuspended in 195 μL of binding buffer. 5 μL of Annexin V-FITC and 10 μL propidium iodide stock solution was added to each sample for 20 min and the cell apoptosis rate was analyzed by a flow cytometer.

***Cell cycle experiments:*** After treatment with 20 nM of TP, AS-TP and CO-TP, MDA-MB-231 cells were incubated with trypsin to prepare for single-cell suspension and then fixed in 70% ethanol at 4 ℃ overnight. Subsequently, cell cycle staining was performed according to the supplier manual. Samples were run on flow cytometry, and DNA content and cell-cycle distribution were analyzed by the ModFit software.

***Reactive oxygen species (ROS) detection:*** Intracellular reactive oxygen species (ROS) level was monitored as previously described. In short, MDA-MB-231 cells were seeded in a 6-well plate and adhered overnight. After 24 h treatment, cells were collected and suspended in diluted DCFH-DA solution for 30 min. The changes in the FCM of the ROS probe were determined using a flow cytometry in the FITC channel.

***Mitochondrial membrane potential (MMP) determination:*** After co-cultivation with AS-TP, MDA-MB-231 cells were stained with JC-1 dye at 37 ℃ for 30 min. Fluorescence in situ for the detection, membrane potential change was assessed by confocal laser scanning microscopy. For FCM detection, cells were harvested for the analysis of mitochondrial membrane potential change using a flow cytometer in FITC and PE channels.

***Weston blot assay:*** The protein expression levels were analyzed by Weston blot. MDA-MB-231 cells were lysed in RIPA buffer supplemented with protease and phosphatase inhibitors and protein content was quantitated using the BCA Protein Assay. Equal amounts of protein were separated on SDS PAGE gel, transferred to PVDF membrane, and blocked using 0.5% skim milk for 2 h. Membranes were left to incubate overnight at 4 ℃ with primary antibodies and followed by the secondary antibody for 1 h. The bands were exposed using ECL Western blotting Kit, visualized by the ChemiDoc Touch imaging system (Bio-Rad, Hercules) and quantified densitometry by the Image J software.

***Tumor model:*** Specific pathogen-free (SPF) female BALB/c nude mice (6-7 weeks old, 18-20g) were purchased from Beijing Huafukang Bioscience Co., Inc. Animal protocols and procedures were approved by the Animal Research Ethics Committee of Chengdu University of Traditional Chinese Medicine (National Animal Use License number: SYXK (Chuan) 2020-124, Chengdu, China). All mice were housed individually under the SPF animal facility of the Institute of Chengdu University of Traditional Chinese Medicine in a temperature-controlled (22-26 ℃) and light-controlled (12-hour light/12-hour dark cycle) room. The nude mice were subcutaneously inoculated with 1 × 10^6^ MDA-MB-231 cells on their right armpit. After the tumor size reached about 50-100 mm^3^, all mice were randomly divided into several groups.

***In vivo distribution experiment:*** To evaluate the bio-distribution of AS-TP conjugate, equal molar quantities of FAM-labeled AS-TP and CO-TP was injected into balb/c nu mice or the MDA-MB-231 xenograft mouse model through the tail vein. *In vivo* bioluminescence imaging was performed using a small animal live imaging system (IVIS Spectrum, Perkin Elmer) at specified time points (2 h, 4 h and 8 h). At the same time, after administration, the nude mice were sacrificed at different times (2 h, 4 h and 8 h). The tumors or organs were collected to evaluate the biodistribution of AS-TP conjugate by detecting the fluorescence intensity using a small-animal imaging system.

***Pharmacokinetic study:*** Seven-week-old Balb/c normal mice were used to evaluate the pharmacokinetics (PK) of AS-TP conjugate. AS-TP of 2.5 mg/kg was intravenously injected into BALB/c mice. Blood samples (100 μL) were collected by retro-orbital venous at different time points (3 min, 5 min, 15 min, 30 min, 1 h, 2 h, 4 h, 8 h, 12 h, 16 h and 24 h). Add 1 mL water into each sample, completely mix and centrifuge for 5 min at 12000 rpm. Then the supernatant was concentrated by vacuum freezing and dissolved in 50 μL water. The AS-TP content in each sample was analyzed by UPLC. The pharmacokinetic properties of AS-TP conjugate were performed using the DAS2.0 software.

***In vivo efficacy evaluation:*** In order to better assess the efficacy of AS-TP on solid tumor models, different drugs (AS1411, AS-TP, CO-TP and TP) were injected intravenously through the tail vein into nude mice once every fourth day for 7 times. The injection dose of each drug contained an equal concentration. As a comparison, saline solution was injected in the control group. For the second pharmacodynamics experiment, a total of 24 tumor-bearing mice were randomly assigned to 4 groups. The control group received a tail vein injection of saline. The Eribulin group received a tail vein injection of Eribulin (0.12 mg/kg; HY-13442; Merck). The nab-PTX group received a tail vein injection of Nab-Paclitaxel (30 mg/kg; HY-P99974; Merck). The AS-TP group received a tail vein injection of AS-TP (27 mg/kg). All samples were dissolved in normal saline and all animals were injected for 8 times, with a vehicle dosage of 10 mL/kg. During the experiment, tumor size were measured every four days, and tumor volume was calculated (volume (mm^3^) = length×width^2×0.5). The relative tumor volume (RTV) is Vt/V0, where V0 is the volume before treatment and Vt is the volume after treatment. The *in vivo* anticancer activity of each treatment was evaluated by T/C% value. T/C% is calculated as (T_RTV_/C_RTV_) ×100%. Among them, T_RTV_ and C_RTV_ represent the RTV of the treatment group and the control group respectively. After the last treatment, mice were euthanized, and the tumors were collected and weighed. The calculation formula of tumor growth inhibition (TGI): $TGI=(1-{T_{tumor weight}}/{C_{tumor weight}})\times100$. Throughout the experiment, the mice's weight was recorded every 4 days, and the percentage change in weight was calculated using the formula: Weight percentage of mice = (M_t_/M_0_) * 100%, where M_t_ represents the weight of the mice during the experiment and M_0_ is their initial weight. After the last treatment, mice were euthanized, the blood and major organs were collected and weighed.

***Hematoxylin and eosin (H&E) and immunohistochemistry:*** The tumors and major organs (heart, kidney, liver, lung and spleen) were fixed in 4% paraformaldehyde and then paraffin-embedded. The paraffin sections were dewaxed in xylene and stained with hematoxylin and eosin, followed by xylene transparent and sealed with neutral gum. For Ki67 immunohistochemical studies, tumor paraffin sections were incubated with antibodies against Ki67. Slides were washed with PBS, incubated in DAB reagent, and then counterstained with hematoxylin. For TUNEL immunohistochemistry staining, paraffin sections washed in PBS were incubated proteinase K for 30 min, followed treated with TUNEL dye liquor and the nuclei were counterstained with DAPI. Images of all slices were captured using Digital Pathology Scanning System (NanoZoomer S60, Hamamatsu Photonics).

***Blood biochemical index analysis:*** Blood samples were left to stand for 30 min and centrifugated at 4500 rpm for 15 min, and the supernatant was transferred into the centrifuge tube. The index changes of the liver (ALT, AST) and renal (UA, UREA) function were analyzed using an automatic biochemical analyzer (Mindray, BS-360S).

***Routine blood tests:*** Blood was collected for periodic blood testing to assess the potential toxicity of nab-PTX, Eribulin and AS-TP. Blood cell counts were performed using an automated veterinary hematology analyzer (Hemavet 950FS; Drew Scientific) and a preprogrammed mouse calibration mode.

***Statistics:*** In general, results were confirmed in n ≥ 3 independent experiments with technical repeats. Data analysis was performed with GraphPad Prism Software (GraphPad Software Inc., version 8.0). As indicated in the figure legends, all values were presented as means ± SD of individual samples, one-sided t-test was used for the single comparison between two groups. If *p* < 0.05, there was a significant difference; if *p* < 0.01, difference was significant; if *p* < 0.001, difference was extremely significant; if *p* > 0.05, there was no significant difference. Unless otherwise specified, n = 3 independent experiments were performed.

**Reference**

1 Marie, Z. *et al.* Toward Improved Description of DNA Backbone: Revisiting Epsilon and Zeta Torsion Force Field Parameters. *Journal of Chemical Theory and Computation*, doi:10.1021/ct400154j (2013).

2 Gydo, C. P. v. Z. *et al.* The HADDOCK2.2 Web Server: User-Friendly Integrative Modeling of Biomolecular Complexes. *Journal of Molecular Biology*, doi:10.1016/j.jmb.2015.09.014 (2016).
