# Supplementary material for: Aptamer-functionalized triptolide with release controllability as a promising targeted therapy against triple-negative breast cancer
Source: J Exp Clin Cancer Res. 2024 Jul 25;43:207. doi: 10.1186/s13046-024-03133-5 (PMC11270970; doi:10.1186/s13046-024-03133-5)
Supplement: Supplementary file 2 — Supplementary Material 2 [file 13046_2024_3133_MOESM2_ESM.docx]

**Point-by-point response to the comments from reviewer #2 of Journal of Experimental & Clinical Cancer Research**

| **Items** | **Comments** | | **Response** | **Location in updated manuscript or supporting information** |
| --- | --- | --- | --- | --- |
| Overall remarks | In the paper entitled "Aptamer-functionalized triptolide with release controllability as a promising targeted therapy against triple-negative breast cancer" the authors describe a novel modification to the already known AS1411, an aptamer of Nucleolin protein, which consists in a pH-sensitive conjunction with the triptolide (TP) molecule. The compound (AS-TP) obtained by the authors was able to maintain the targeting selectivity of AS1411 towards tumor cells expressing high levels of nucleolin, poorly expressed in normal cells, and the cytotoxic properties of triptolide drug (TP). Additionally, the authors improved the water solubility and the bioavailability of AS-TP compared to the original compounds. Finally, according to the good antitumor efficacy of AS-TP obtained in preclinical in vitro and in vivo models of human breast cancer, the compound seems to be very promising and deserves to be taken into consideration for possible therapeutic developments.  Overall, the experiments are reasonably well-done, and the manuscript is well written. However, the work will be suited for publication once the following issues have been addressed. | | Thanks for your kindly suggestion. We totally agree with the reviewer's suggestion. | N/A |
| Detailed remarks | 1 | In the introduction section I suggest specifying that nucleolin is differentially expressed in normal compared to cancer cells as it is localized primarily in the nucleus, while on cancer cells it is also present in the cytoplasm and at the cell surface (Mongelard et al., 2007). | Thank you for your kind suggestion. We have supplemented the description of the differential expression of NuP in normal and cancer cells in the Introduction section. | Page 4 Line 4-7 |
|  | 2 | At this regard, I have some perplexities about the IF experiments reported in Figure S1 which were conducted by directly incubating viable cells with the primary Ab anti-nucleolin. The authors are encouraged to perform a classical IF protocol by incubating the primary Ab on already fixed and permeabilized cells, as it should allow to also obtain a nuclear staining of the nucleolin protein. | Thank you for your kind suggestion. In this study, in order to verify the expression of NuP in different cell lines, we employed cellular immunofluorescence to quantify NuP on cell membranes. Furthermore, in order to avoid the interference of NuP expression in the cell nucleus, the membrane permeability reagent was not adopted in this study to permeate the cell membrane, meanwhile, the antibody concentration and the length of antibody incubation were strictly controlled, so that only the NuP on the surface of the cell membrane was finally investigated. | Page 6 Line 11-17  Supporting Information Page 48 Line 30-34 |
|  | 3 | The authors could make the results section easier to follow by describing all in vivo experiments after the in vitro ones and by moving the Figure 8 to the supplementary figures. Moreover, the authors overlooked a fundamental recommendation of submission guidelines, that is "Journal of Experimental & Clinical Cancer Research will not consider any new submissions that contain images of animals bearing tumors. Tumors should be excised and pictured next to a ruler for size comparison". Therefore, Figure 9F must be modified accordingly. | Thank you for your kind suggestion. The biological evaluation in this paper was divided into five parts, including the NuP targeting of AS-TP, the endocytosis mechanism of AS-TP, the antitumor effect and mechanism of AS-TP, the pharmacokinetic profile and in vivo safety of AS-TP, and the comparation of AS-TP with clinical antitumor drugs. To make the paper accessible, we elucidated the in vivo antitumor effects of AS-TP in Figure 7 and explained the in vitro antitumor mechanism of AS-TP in Figure 8, respectively. Meanwhile, Figure 9 has been revised in this paper in accordance with the journal submission guidelines. | Page 15 Line 1-22  Page 16 Line 1-4  Page 35 Figure 7  Page 36 Figure 8 Page 40 Figure 10 F |
|  | 4 | In order to strengthen the conclusions of this study, at least the main in vitro experiments performed in MDA-MB-231 cells should be repeated by using an additional TNBC cell line. | Thank you for your kind suggestion. We added additional TNBC cell lines and supplemented the corresponding experiments, including (1) The expression of nuclein receptor was measured in TNBC cell lines MDA-MB-468 and 4T1; (2) The cytotoxic effects of AS-TP and CO-TP on TNBC cell lines MDA-MB-468 and 4T1; (3) The endocytosis of FAM-AS-TP and FAM-CO-TP by TNBC cell line MDA-MB-468 and 4T1; (4) The co-localization of nuclein receptors and FAM-AS-TP in TNBC cell lines MDA-MB-468 and 4T1. | Page 6 Line 17-20  Page 10 Line 17-22,  Page 11 Line 1-8,  Page 12 Line 2-3,  Page 31 Figure 5D;  Supporting Information  Page 3 Figure S2  Page 4 Figure S3  Page 32 Figure S29  Page 33 Figure S30  Page 34 Figure S31  Page 38 Figure S35 |
| Minor comments | 1 | In the legends of various figures (i.e. Figure 6 and 7) the doses and times of administration of the drugs are missing, they should be indicated where omitted. | Thank you for your kind suggestion. The missing doses and times of administration information in the figures have been supplemented. | Page 35 Figure 7  Page 36 Figure 8;  Supporting Information  Page 48 Line 35-43  Page 49 Line 9-16 |
|  | 2 | In the Figure 1D blank cells are represented in the legend with a different color than that showed in the relative chart. | Thank you for your kind suggestion. The color representing the blank cell in Figure 1D has been corrected. | Page 26 Figure 1D |
